# Interaction- and asymmetry-aware facial blendshape analysis for objective quantification of Parkinsonian hypomimia

**DOI:** 10.1038/s41531-026-01579-2

**Published:** 2026-09-23

**Authors:** Berkan Koyak, Timo Menzel, Martin Rodemann, Guido Hennes, Annika Spottke, Eva Saxler, Janis Bedarf, Jennifer Faber, Michael Sommerauer, Patrick Weydt, Martin Reuter, Mario Botsch, Ullrich Wuellner, N. Ahmad Aziz

**Affiliations:** 1 University of Bonn, University Hospital Bonn, Clinic for Parkinson’s Disease, Sleep Disorders, and Movement Disorders, Bonn, Germany; 2 German Center for Neurodegenerative Diseases (DZNE), Bonn, Germany; 3 Faculty of Computer Science, TU Dortmund University, Dortmund, Germany; 4 University of Bonn, University Hospital Bonn, Clinic for Neuroradiology, Bonn, Germany; 5 A.A. Martinos Center for Biomedical Imaging, Massachusetts General Hospital, Boston, MA USA; 6 Department of Radiology, Harvard Medical School, Boston, MA USA; 7 University of Bonn, University Hospital Bonn, Institute for Medical Biometry, Informatics, and Epidemiology (IMBIE), Bonn, Germany

**Keywords:** Biomarkers, Diseases, Medical research, Neurology, Neuroscience

## Abstract

Reduced facial expressivity (hypomimia) is an early motor feature of Parkinson’s disease (PD), yet its assessment still relies on subjective, coarse-grained clinical scales. Using ExpressionTracker, a consumer-grade application built on Apple’s ARKit framework, we derived 261 facial expression features during standardised cued expression tasks in 34 people with PD and 36 healthy controls (HC). Beyond the expected reduction in movement amplitude, PD was characterised by increased facial asymmetry and attenuated activity of ipsilateral mouth and eye regions. Interaction and asymmetry features were prominent among the between-group differences and explained 38 to 42 percent of the variance in clinician-rated hypomimia. For diagnostic classification (PD versus HC) under nested cross-validation, the best model (gradient boosting machine, GBM) reached an area under the receiver operating characteristic curve (AUC) of 0.834 (95% CI 0.733 to 0.923), followed by XGBoost (0.810, 95% CI 0.702 to 0.906) and light gradient boosting machine (LightGBM; 0.804, 95% CI 0.697 to 0.899). Engineered ARKit features outperformed an amplitude-only baseline (maximum AUC 0.75, 95% CI 0.63 to 0.86) and a demographic confounder-only baseline using age and sex (maximum AUC 0.68, 95% CI 0.55 to 0.80). These results indicate that automated facial blendshape analysis captures multidimensional motor dysfunction beyond amplitude reduction alone, positioning ExpressionTracker as a candidate digital biomarker for community screening, disease monitoring, and drug-efficacy assessment in future clinical trials.

## Introduction

Hypomimia, or reduced facial expressivity, is one of the earliest motor hallmarks of Parkinson’s disease (PD) and affects both spontaneous and voluntary facial movements^1^. Hypomimia can precede the clinical diagnosis of PD by up to ten years in individuals with idiopathic REM sleep behaviour disorder^2,3^. Current assessment of hypomimia relies on semiquantitative clinical judgement using item 3.2 (facial expression) of the Movement Disorder Society Unified Parkinson’s Disease Rating Scale Part III (MDS-UPDRS III), which grades hypomimia from reduced blink frequency, diminished perioral movement including spontaneous smiling, and parted lips at rest^4^. Although valuable and extensively validated in routine practice, the MDS-UPDRS III was not developed for early-stage PD and remains subject to substantial interrater variability and to floor and ceiling effects^5–7^. Moreover, it is a nonlinear ordinal scale rather than an interval measure, so that differences between adjacent scores are not necessarily equivalent. As disease-modifying therapies advance toward clinical trials yet repeatedly fail to demonstrate efficacy, there is a pressing need to characterise and quantify all phenomenological aspects of PD, including hypomimia, in an objective and standardised manner.

Cumulative evidence supports computer vision for automated hypomimia detection, but the approaches applied to date have been heterogeneous. A recent systematic review groups them into method families that span task-based paradigms such as reading aloud, emotion-elicitation tasks such as posed smiling, anger, or disgust, and data-mining of spontaneous expressions extracted from internet images and videos^8–12^. Within these families, methods range from facial-landmark and distance metrics, through action-unit pipelines such as OpenFace coupled with logistic regression, to deep-learning classifiers trained on video, often with data augmentation to offset small samples, and to studies of spontaneous expressivity^13–15^. Two limitations recur across these families. First, many paradigms rely predominantly on visual emotion-recognition cues without accounting for the impaired emotion recognition frequently observed in PD, which can confound measurements of voluntary facial expressiveness^16–19^. Second, although facial expressions are inherently synergistic, requiring distinct combinations of activated and inactivated muscle groups, the asymmetry and interaction properties of facial musculature and their role in hypomimia remain largely unexplored^20–22^. A standardised assessment that captures cued facial movements while explicitly modelling asymmetry and interaction therefore remains a missing component in the quantitative characterisation of hypomimia.

To address these gaps, we used the smartphone and tablet application ExpressionTracker, which performs automated facial expression analysis within five to ten minutes^23^. ExpressionTracker is built on Apple’s ARKit framework and adapted from an application originally developed by Menzel et al. for photorealistic three-dimensional avatar creation, enabling high-resolution, personalised facial motion tracking on consumer-grade devices^23^. ARKit quantifies facial blendshapes, that is, the degree of transformation applied to a neutral facial mesh that is personalised to each individual’s facial geometry through the device TrueDepth sensor, providing person-specific calibration of baseline anatomy. Our aim was to quantify hypomimia objectively and to test whether asymmetry and interaction features, in addition to movement amplitude, improve the characterisation and classification of PD. Our contributions are threefold: (i) an engineered facial-expression feature space that augments movement amplitude with aggregation, interaction, and asymmetry features; (ii) a nested cross-validated machine-learning analysis, with all data-dependent steps fitted within the training folds, that discriminates PD from healthy controls (HC) and is benchmarked against amplitude-only, demographic, and OpenFace baselines; and (iii) group-level and correlation analyses that link specific blendshape features to clinician-rated motor severity.

## Results

### Descriptive statistics

The study included 36 HC (17 male, 19 female) and 34 people with PD (24 male, 10 female). Mean age was 64.2 years (standard deviation [SD] 14.9; range 23–84) in HC and 61.2 years (SD 8.4; range 46–78) in PD, with a mean motor symptom duration of 6.4 years (SD 5.1; range 1–19) in PD. The mean MDS-UPDRS III sum score was 17.0 (SD 10.4; range 5–47). The facial expression subitem (MDS-UPDRS III item 3.2) had a mean of 1.2 (SD 1.0; range 0–3), with 12 participants scoring 0, four scoring 1, 17 scoring 2, and one scoring 3. Hoehn and Yahr (H&Y) stage was distributed as Stage 1 in 17, Stage 2 in 15, and Stage 3 in two participants. Demographic and clinical characteristics are summarised in (Table 1).

**Table 1:** Demographics and clinical characteristics

Characteristic	HC	PD
*N* (subjects)	36	34
Age, years (mean ± SD)	64.17 ± 14.92	61.2 ± 8.4
Sex (M/F)	17 (47.2%) / 19 (52.8%)	24 (70.6%) / 10 (29.4%)
Motor disease duration, years (mean ± SD)	-	6.4 ± 5.1
PD with DBS, *n* (%)	-	3 (8.8%)
PD with HIFU, *n* (%)	-	2 (5.9%)
PD with both DBS and HIFU, *n* (%)	-	2 (5.9%)
PD phenotype, *n* (%)	-	Tremor-dominant 15 (44.1%); Akinetic-rigid 10 (29.4%); Equivalent 7 (20.6%); Accompanying ET 2 (5.9%)
Hoehn and Yahr stage, *n* (%)	-	Stage 1: 17 (50.0%); Stage 2: 15 (44.1%); Stage 3: 2 (5.9%)
UPDRS III item 3.2 “Facial Expression”, *n* (%)	0.00 ± 0.00	0: 12 (35.3%); 1: 4 (11.8%); 2: 17 (50.0%); 3: 1 (2.9%)
UPDRS III sum (mean ± SD)	0.54 ± 1.10	17.0 ± 10.4
UPDRS IV sum (mean ± SD)	-	2.4 ± 3.9
LED (mg, mean ± SD)	-	443.2 ± 280.4

Continuous variables are presented as mean ± SD (range) where available; categorical variables as *n* (%). Abbreviations: PD, Parkinson’s disease; HC, healthy controls; SD, standard deviation; DBS, deep brain stimulation; HIFU, high-intensity focused ultrasound; ET, essential tremor; LED, levodopa equivalent dose.

*PD* Parkinson’s disease, *HC* healthy controls, *SD* standard deviation, *DBS* deep brain stimulation, *HIFU* high-intensity focused ultrasound, *ET* essential tremor, *LED* levodopa equivalent dose.

### Between-group comparisons

**Fig. 1:**
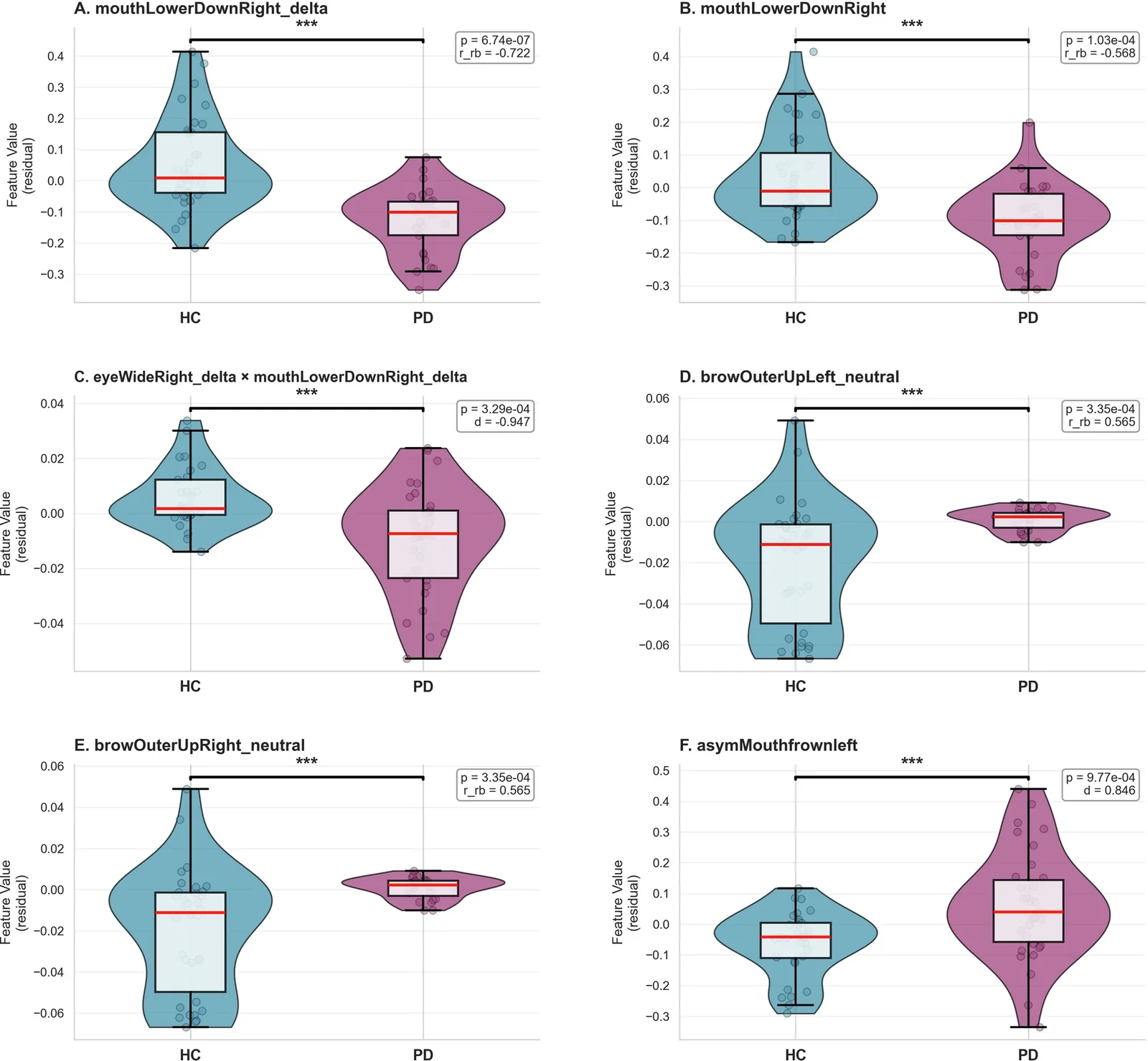
Between-group differences in facial blendshape features (PD versus HC). Violin plots show the distribution density (coloured areas); boxplots show the median and quartiles (white boxes with a red median line); and individual data points are overlaid (semi-transparent dots). Significance bars indicate *p* < 0.001 after Meff-adjusted Bonferroni correction. The symbols **A–F** denote the facial blendshape features surviving the Meff-adjusted Bonferroni correction.  Effect sizes: d, Cohen’s d (t-test); r_rb, rank-biserial correlation (Mann–Whitney U test). Abbreviations: Meff effective number of independent tests, IQR interquartile range, HC healthy controls, PD Parkinson’s disease.

Six facial blendshape features differed significantly between the PD and HC groups with moderate-to-large effect sizes. ***mouthLowerDownRight*** differed significantly between groups in both the delta condition (*p* = 6.74 × 10^−7^; rank-biserial *r* = −0.722) and the activated condition (*p* = 1.03 × 10^−4^; rank-biserial *r* = −0.568). Compared with HC, PD showed a markedly reduced dynamic range of lower lip depression (PD: −0.126 ± 0.10 versus HC: 0.049 ± 0.14) as well as reduced voluntary activation of the lower lip depressor muscles (PD: −0.100 ± 0.11 versus HC: 0.034 ± 0.14) (Fig. 1A, B).

***browOuterUpRight*** and ***browOuterUpLeft*** both differed significantly in the neutral condition (*p* = 3.35 × 10^−4^; rank-biserial *r* = 0.565 for both), with PD exhibiting a bilaterally elevated resting brow position relative to HC (PD: 0.001 ± 0.005 versus HC: −0.020 ± 0.03). The identical *p* values and effect sizes for both sides indicate a symmetric alteration of upper facial resting tone (Fig. 1D, E).

***asym_mouthFrownLeft*** in the activated condition was significantly greater in PD (*p* = 9.77 × 10^−4^; Cohen’s *d* = 0.846), indicating greater leftward asymmetry of the mouth corner depressor muscles (PD: 0.060 ± 0.17 versus HC: −0.063 ± 0.11) (Fig. 1F). The ***eyeWideRight*** by ***mouthLowerDownRight*** interaction feature in the delta condition also differed significantly between groups (*p* = 3.29 × 10^−4^; Cohen’s *d* = −0.947) (PD: −0.010 ± 0.020 versus HC: 0.006 ± 0.011) (Fig. 1C).

Asymmetry and interaction features were prominent among the significant between-group differences, whereas no aggregation feature survived the effective-number-of-tests (*Meff*) adjusted Bonferroni correction. A complete overview of all between-group comparisons is provided in (Supplementary Table 4).

### Correlation analysis

**Fig. 2:**
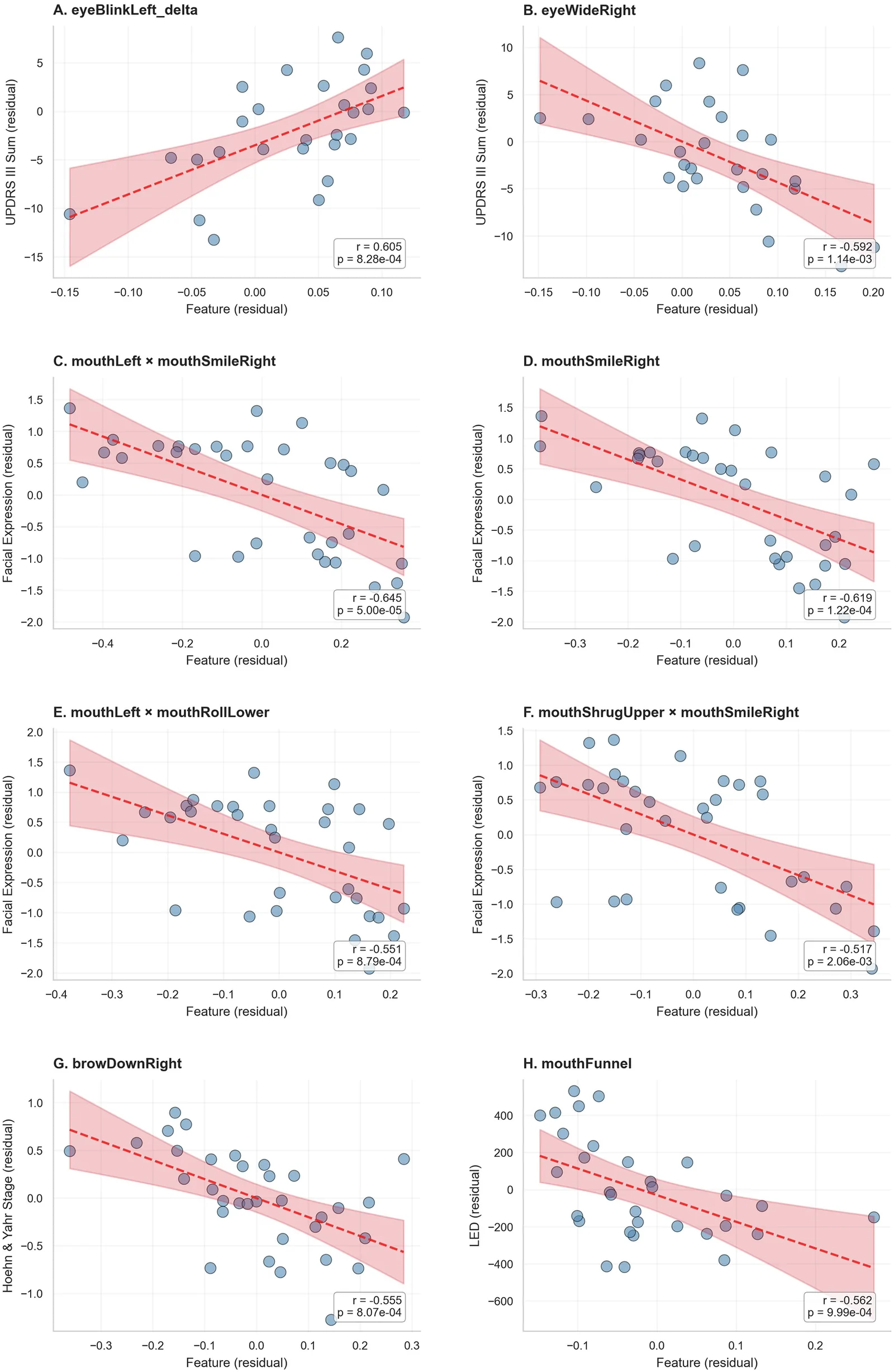
Facial blendshape correlations with motor dysfunction in Parkinson’s disease. Features exhibiting the highest correlation coefficients are plotted against the respective clinical scale and medication load. Data are residualised for age, sex, and LED (and, for the LED panel, for age, sex, and H&Y stage). Regression lines (red dashed) are shown with their 95 percent CI (red shaded area). Interaction features (shown as feature1 by feature2) are pairwise products of top-ranking base blendshapes. The symbols **A–H** denote all correlations that survived Meff-adjusted Bonferroni correction. Abbreviations: LED, levodopa equivalent dose (mg/day); H&Y, Hoehn and Yahr; Meff, effective number of independent tests; CI, confidence interval.

Eight features survived Bonferroni correction within their respective feature families. Seven associations were negative, with greater feature activation corresponding to lower clinical severity, and one was positive. An overview is provided Fig. 2. ***eyeBlinkLeft*** in the delta condition correlated positively with the MDS-UPDRS III sum score (*r* = 0.605; *p* = 8.28 × 10^−4^), such that a greater dynamic range of left eyelid closure was associated with higher overall motor impairment, whereas ***eyeWideRight*** in the activated condition correlated negatively (*r* = −0.592; *p* = 1.14×10^−3^) (Fig. 2A, B). For clinician-rated hypomimia (MDS-UPDRS III item 3.2), ***mouthSmileRight*** in the activated condition was negatively correlated (*r* = −0.619; *p* = 1.22 × 10^−4^), and the ***mouthLeft*** by ***mouthSmileRight*** interaction yielded the strongest association in the entire correlation analysis (*r* = −0.645; *p* = 5.00 × 10^−5^), explaining 42 percent of the variance in clinician-rated hypomimia (Fig. 2C, D). The ***mouthLeft*** by ***mouthRollLower*** interaction (*r* = −0.551; *p* = 8.79 × 10^−4^) and the ***mouthShrugUpper*** by ***mouthSmileRight*** interaction (*r* = −0.517; *p* = 2.06 × 10^−3^) corroborated this pattern, all involving the lower perioral musculature in the activated condition (Fig. 2E, F).

***browDownRight*** in the activated condition was negatively correlated with H&Y stage (*r* = −0.555; *p* = 8.07 × 10^−4^), indicating that reduced voluntary eyebrow depression was associated with more advanced disease stage (Fig. 2G). ***mouthFunnel*** in the activated condition was negatively correlated with the levodopa equivalent dose (LED) (*r* = −0.562; *p* = 9.99 × 10^−4^), such that individuals with higher dopaminergic medication load showed a reduced capacity for voluntary lip pursing (Fig. 2H). A complete list of tested correlations is provided in (Supplementary Tables 5– 9).

### Machine-learning classification of PD versus HC

**Fig. 3:**
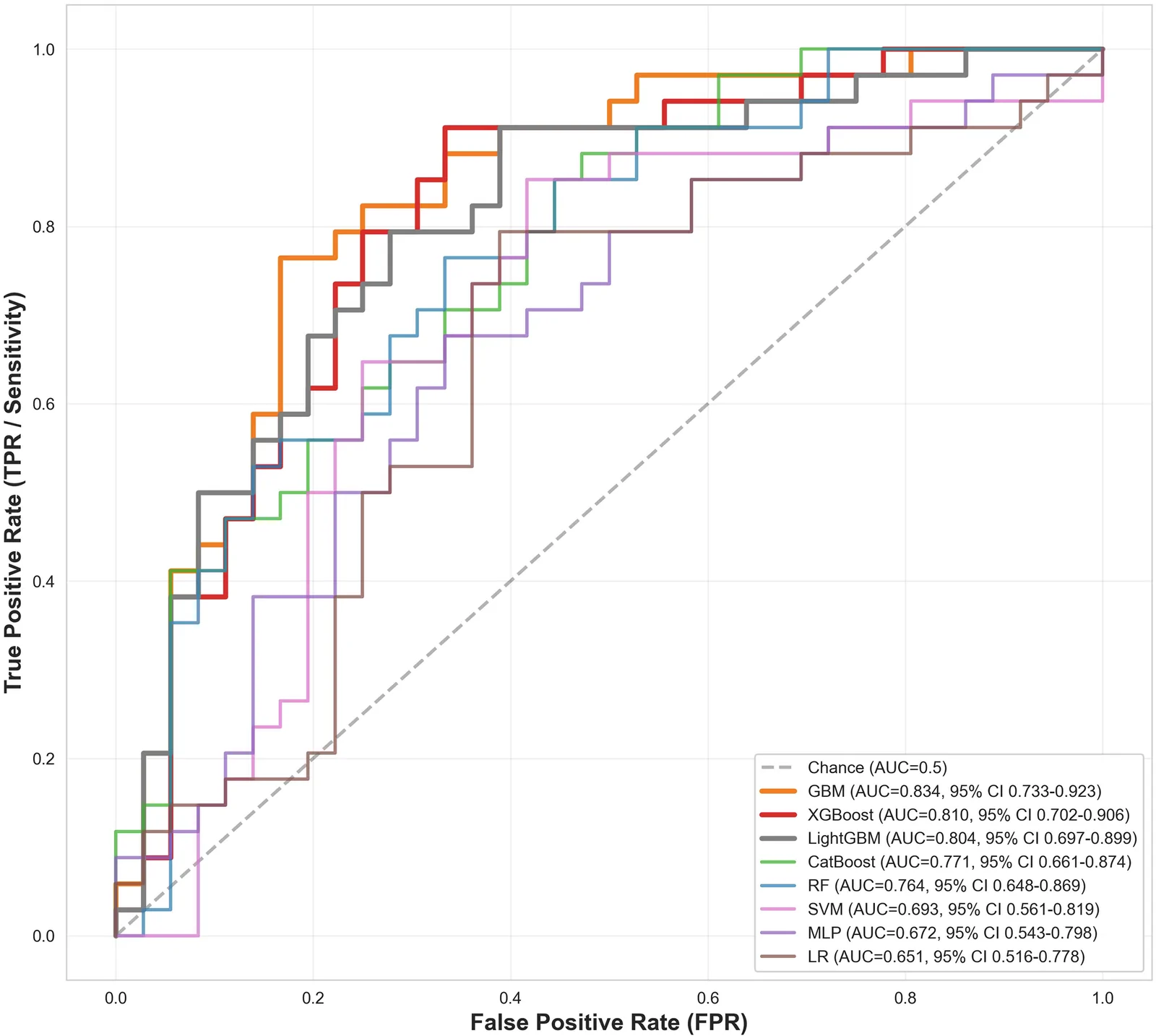
PD versus HC ROC curves. Receiver operating characteristic (ROC) curves for the eight classifiers discriminating Parkinson’s disease (PD) from healthy controls (HC) under nested cross-validation (outer five-fold cross-validation repeated ten times). Curves are computed from pooled out-of-fold predicted probabilities; the area under the curve (AUC) with its bootstrap 95 percent CI is given for each model in the legend. The diagonal dashed line denotes chance (AUC 0.5). Positive class, PD. Abbreviations: GBM gradient boosting machine, XGBoost extreme gradient boosting, LightGBM light gradient boosting machine, CatBoost categorical boosting, RF random forest, SVM support vector machine, MLP multilayer perceptron, LR logistic regression.

**Fig. 4:**
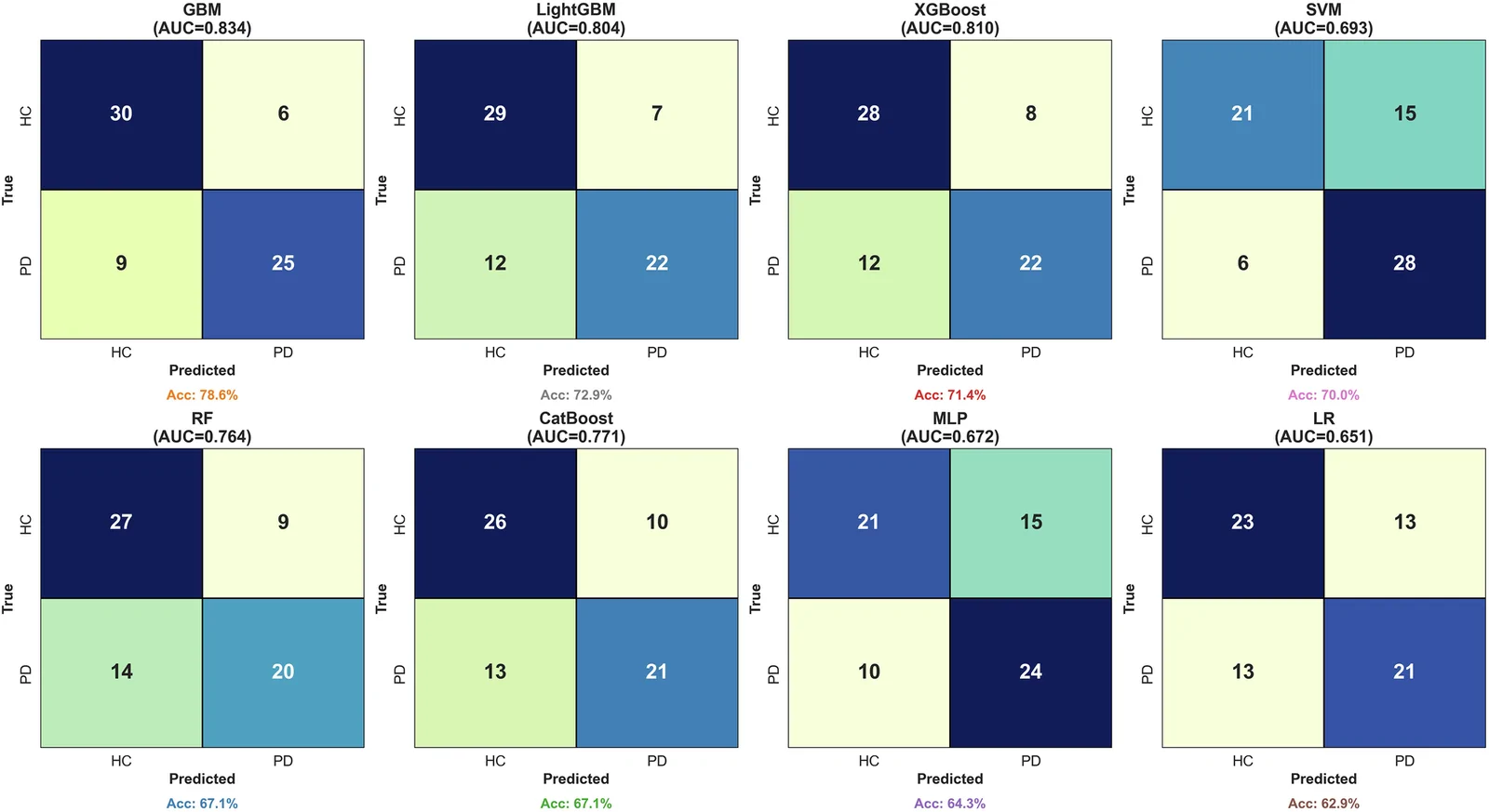
PD versus HC confusion matrices. Confusion matrices for the eight classifiers at a fixed decision threshold of 0.5, computed from pooled out-of-fold predictions across the repeated nested cross-validation. Rows are true labels and columns are predicted labels; PD denotes Parkinson’s disease and HC denotes healthy controls. Diagonal cells are correct predictions and off-diagonal cells are misclassifications. Models are ordered by accuracy. Abbreviations as in Fig. 3.

**Fig. 5:**
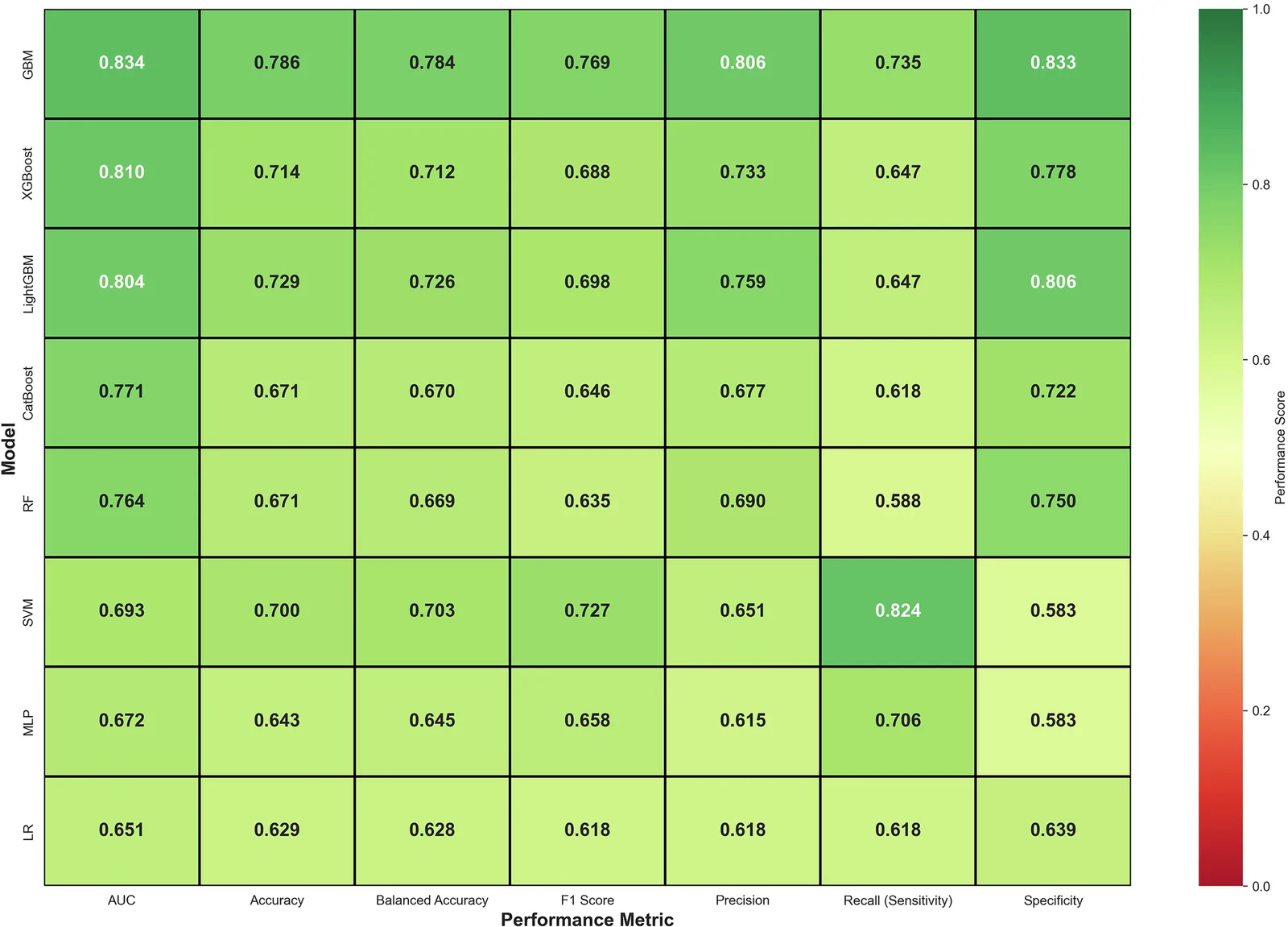
PD versus HC performance metrics. Colour-coded heatmap of performance metrics for the eight classifiers discriminating PD from HC, with PD as the positive class and a fixed 0.5 threshold. Green indicates higher and red lower values (range 0–1). Metrics are derived from pooled out-of-fold predictions under nested cross-validation. Abbreviations: AUC area under the receiver operating characteristic curve; other abbreviations as in Fig. 3.

For the diagnostic task (PD versus HC; positive class PD; 34 PD and 36 HC), the gradient boosting machine (GBM) achieved the highest discrimination, with an area under the receiver operating characteristic curve (AUC) of 0.834 (95% CI 0.733 to 0.923), followed by extreme gradient boosting (XGBoost; 0.810, 95% CI 0.702 to 0.906), light gradient boosting machine (LightGBM; 0.804, 95% CI 0.697 to 0.899), categorical boosting (CatBoost; 0.771, 95% CI 0.661 to 0.874), random forest (RF; 0.764, 95% CI 0.648 to 0.869), support vector machine (SVM; 0.693, 95% CI 0.561 to 0.819), multilayer perceptron (MLP; 0.672, 95% CI 0.543 to 0.798), and logistic regression (LR; 0.651, 95% CI 0.516 to 0.778) (Figs. 3, 5). At a fixed decision threshold of 0.5, the GBM reached an accuracy of 0.786, a balanced accuracy of 0.784, a sensitivity of 0.735, a specificity of 0.833, and an F1 score of 0.769 (Figs. 4, 5).

**Fig. 6:**
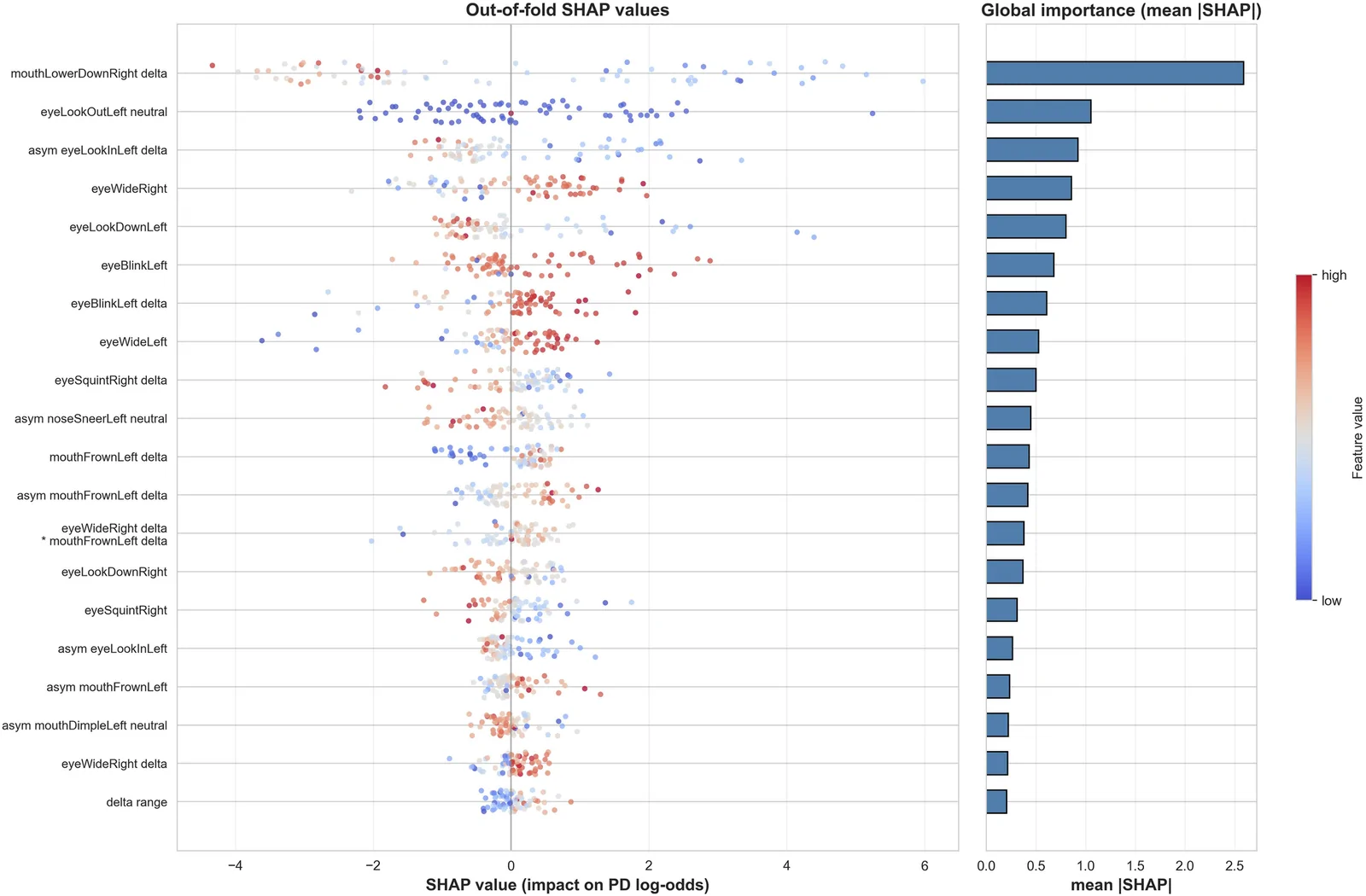
PD versus HC: SHAP importance for the GBM. Feature importance for the best-performing model (gradient boosting machine, GBM). Left, out-of-fold SHAP (SHapley Additive exPlanations) values for the most influential features, aggregated across the 50 outer test folds; each point is one participant, colour encodes the feature value (low to high), and the horizontal position is the signed contribution to the log-odds of PD. Right, global importance ranked by mean absolute SHAP value. Feature names combine an ARKit blendshape with a measurement condition (neutral, activated, or delta); the prefix asym denotes a left-minus-right asymmetry index. Abbreviations: SHAP SHapley Additive exPlanations, GBM gradient boosting machine, PD Parkinson’s disease, HC healthy controls.

To identify the features that drove the best-performing model, we computed out-of-fold SHAP (SHapley Additive exPlanations) values for the GBM, aggregated across the 50 outer test folds (Fig. 6). The dynamic range of right lower lip depression (***mouthLowerDownRight***, delta condition) was by far the most influential feature, followed by baseline lateral gaze position (***eyeLookOutLeft***, neutral), the asymmetry of inward gaze change during expression (***asym_eyeLookInLeft***, delta), right eye widening (***eyeWideRight***), and left downward gaze (***eyeLookDownLeft***).

To isolate the contribution of the engineered ARKit features, we repeated the identical nested pipeline on three reference feature sets in the same cohort (Supplementary Fig. 6). An amplitude-only baseline restricted to the 52 delta blendshapes reached a maximum AUC of 0.752 (XGBoost; 95% CI 0.632 to 0.862), below the engineered ARKit features. A demographic confounder-only baseline using age and sex as the sole predictors reached a maximum AUC of 0.681 (GBM and MLP; 95% CI 0.555 to 0.802 and 0.552 to 0.800, respectively), indicating that demographic differences alone do not account for the diagnostic signal. Engineered OpenFace action-unit features, evaluated on the same ARKit-selected peak frames, did not discriminate PD from HC (all models at or below chance, maximum AUC 0.436 for logistic regression; 95% CI 0.304 to 0.581); because the frames were selected by ARKit, we interpret this contrast with caution (see Discussion).

A label-permutation test with 1000 shuffles, refitting feature selection on each shuffle, confirmed that observed discrimination exceeded the null for seven of the eight classifiers (GBM empirical *p* = 0.002; XGBoost 0.002; LightGBM 0.003; CatBoost 0.004; RF 0.005; SVM 0.021; MLP 0.038; logistic regression 0.061) (Supplementary Fig. 5).

## Discussion

We found that automated facial blendshape analysis captures differences that correlate with clinical severity across three complementary levels: group-level discrimination between PD and HC, correlation with established clinical severity measures, and machine-learning classification of PD versus HC. The most informative features were not limited to movement amplitude. They also included interaction terms, which reflect combined reduction of two facial effectors on the same side of the face (e.g right eye widening and right lower lip depression), and asymmetry indices, which reflect lateralised motor impairment. These dimensions have been largely overlooked by prior automated approaches.

The strongest and most consistent between-group difference was observed in ***mouthLowerDownRight***, which differed significantly in both the delta and activated conditions and was the most influential feature in the diagnostic model’s SHAP analysis (Fig. 6). This aligns with Novotny et al., who reported that reduced lower lip movement amplitude was among the strongest correlates of nigrostriatal dopaminergic loss and limb bradykinesia and rigidity in an automated video-based study^24^. The predominance of lower facial involvement is anatomically plausible and is supported by a recent action-unit study suggresting that upper facial regions benefit from bilateral cortical motor innervation and are comparatively resilient to unilateral nigrostriatal degeneration, whereas the lower face relies predominantly on contralateral corticobulbar input and is more vulnerable to PD pathology^1,25^.

The spatially differential vulnerability of facial expressivity may extend to accounts of hemihypomimia. In our study this is corroborated by ***asym_mouthFrownLeft*** in the activated condition, which was greater in PD, by the prominence of a left-sided asymmetry index (***asym_eyeLookInLeft***) among the most influential features of the diagnostic model, and by the ***eyeWideRight*** and ***mouthLowerDownRight*** interaction in the delta condition, among the largest between-group effects, which together could be interpreted as suggestive of right-sided hemihypomimia in our cohort (Figs. 1C, F, 6). Hemihypomimia has been documented in photographic and videographic studies based on blinded clinical judgement, ipsilateral to the most affected body side^26,27^. Ozekmekci et al. identified hemihypomimia in 13 of 204 H&Y Stage 2 PD patients, consistently on the right side and associated with earlier onset, whereas Guerra-Hiraldo et al. found global facial asymmetry in 46% of PD patients photographed under standardised conditions, with predominant lower face involvement, suggesting that hemihypomimia may be an underappreciated phenotypic feature of PD rather than a rare curiosity^22,28^.

The correlation findings indicate that blendshape-based analysis captures distinct dimensions of motor dysfunction. Clinician-rated hypomimia was best explained by the ***mouthLeft*** by ***mouthSmileRight*** interaction and by ***mouthSmileRight*** alone in the activated condition, accounting for 42% and 38 percent of the variance, respectively (Fig. 2). Consistently, perioral smile-related and lower-lip features were prominent in both the correlation analysis and the diagnostic model.

The resting position of the outer brow in PD was bilaterally elevated, with significantly higher ***browOuterUpRight*** and ***browOuterUpLeft*** values in the neutral condition than in HC. Frontalis hyperactivity as a compensatory mechanism for vertical gaze difficulty is well established in progressive supranuclear palsy (PSP), where it is a useful distinguishing feature^29,30^. Its presence in PD has not been systematically described and should be validated before its neuroanatomical substrate is elucidated. The single positive association, of ***eyeBlinkLeft*** in the delta condition with greater overall motor severity, likely reflects widened palpebral fissures and a staring impression rather than enhanced eyelid motor activity, since all participants reached the maximal blendshape weight of approximately one in the activated condition while the neutral weights varied considerably^31^.

Automated facial analysis for PD can be organised into four broad method families, surveyed comprehensively^8^. Geometric and landmark-based methods measure distances or trajectories between facial keypoints and capture the spatial configuration of the face directly. They are the closest precedent for the present work, although most landmark studies have quantified overall or regional movement amplitude and have not modelled left-right asymmetry or interaction explicitly. Textural and appearance-based methods, whether pixel-based or built on convolutional networks, can reach strong group-level discrimination, but they require large labelled image or video datasets and remain difficult to interpret at the sample sizes typical of PD facial studies^8^. Action-unit methods based on the Facial Action Coding System, such as OpenFace and ARKit pipelines, express the face as anatomically defined movement primitives, which supports interpretation, but their output is only as valid as the underlying action-unit detector. Kinematic methods capture how movement unfolds over time, at the cost of requiring temporal video and, given the small PD facial cohorts available, often data augmentation such as CGAN-based tabular augmentation of facial features^14^. Cutting across these families, paradigms that elicit expressions with emotional stimuli face a further difficulty specific to PD, where impaired expression generation and impaired emotion recognition co-occur and can be hard to disentangle.

Our ARKit blendshape features are, in these terms, primarily a geometric representation computed from a depth-fitted facial mesh, whose coefficients are at the same time conceptually close to facial action units because each encodes a named facial movement. On this base we engineer explicit left-right asymmetry and cross-task interaction features, so that the lateralised and synergistic patterns that landmark studies have rarely modelled become measurable quantities^15^.

Our results indicate both where this approach is informative and where it is likely to fall short. The method is best suited to the voluntary-movement deficits that characterise motor-manifest PD. Perioral amplitude reduction and bilaterally elevated resting brow tone in H&Y Stage 1 and 2 patients were captured with cued tasks, and the engineered asymmetry and interaction features allowed the analysis to resolve lateralised and synergistic patterns that a single global expressivity rating cannot.

Two features of the capture modality are worth highlighting as strengths. First, ARKit blendshape coefficients are normalised to each participant’s own tracked face, ranging from 0 to 1 relative to their neutral expression, and each coefficient corresponds to a named facial movement that maps onto the Facial Action Coding System^32^. The representation is therefore both person-referenced and anatomically interpretable. Two-dimensional action-unit pipelines such as OpenFace instead estimate the same primitives from a generic appearance model^13^. Second, capture relies on the TrueDepth sensor, whose active depth encoding is by design less dependent on ambient lighting and skin texture than the pixel- and CNN-based methods known to be sensitive to these factors^8^.

These strengths are bounded by several limitations. First, although the diagnostic classification is largely free of circular validation, the between-group and correlation analyses are not. They relate facial features to item 3.2 and other clinician-rated scores and therefore retain a degree of dependence on the clinical assessment. Second, all clinical assessments were performed by a single clinician, so inter-rater reliability was not available and the unblinded ratings allow possible expectation bias. Apathy and depression, which can produce hypomimia-like changes, were also not systematically assessed, and future protocols should add a second blinded rater together with screening for these conditions. Third, the approach depends on frontal head pose. ARKit-based facial measurements are known to vary with viewing angle, so residual pose variation can influence blendshape estimates, and an automated frontal-pose check would reduce this source of variance^33,34^. Fourth, fine-grained staging within PD was not attempted, amplitude changes may plateau in moderate disease, and the small number of H&Y Stage 3 participants precludes conclusions about advanced disease. Fifth, the protocol captures voluntary cued movements and does not assess the spontaneous expression profiles that documented in PD^15^. Because co-activation during spontaneous expression may differ from co-activation during instructed poses, future work should pair cued and free-expression paradigms. Sixth, all participants carried an established clinical diagnosis of PD, so performance in prodromal or preclinical populations, where hypomimia may precede motor diagnosis by years and blendshape deviations have not been characterised, is unknown. Seventh, the sample is modest and from a single site, without external validation or test-retest data, so generalisation and reliability across sites and over time remain to be established. The cross-sectional design further precludes causal and progression inferences. Finally, ARKit is proprietary and closed-source, and its blendshape model was designed for consumer face tracking and animation rather than clinical measurement, with the underlying calibration populations undisclosed, so it may not represent the age distribution or facial anatomy of PD cohorts, and its estimator cannot be independently audited. In addition, blendshape coefficients saturate at their upper bound under the most intense expressions, which reduces the resolution available for the most severely affected individuals. A purpose-built, open-source framework trained on age- and sex-matched data would therefore better serve scientific aims.

In conclusion, parkinsonian hypomimia is a multidimensional phenomenon that encompasses not only diminished voluntary movement amplitude but also bilaterally elevated resting brow position, perioral cross-task interaction and hemifacial asymmetry patterns that explain up to 42% of the variance in clinician-rated hypomimia. These features distinguish PD from HC, correlate with clinician-rated hypomimia severity, and support machine-learning discrimination of PD from HC (best model AUC 0.834) using features extractable in under ten minutes from a consumer tablet. With external validation, ExpressionTracker may serve as a digital biomarker for community screening, longitudinal disease monitoring, and drug-efficacy assessment in future clinical trials.

## Methods

### ExpressionTracker application: technical details

**Fig. 7:**
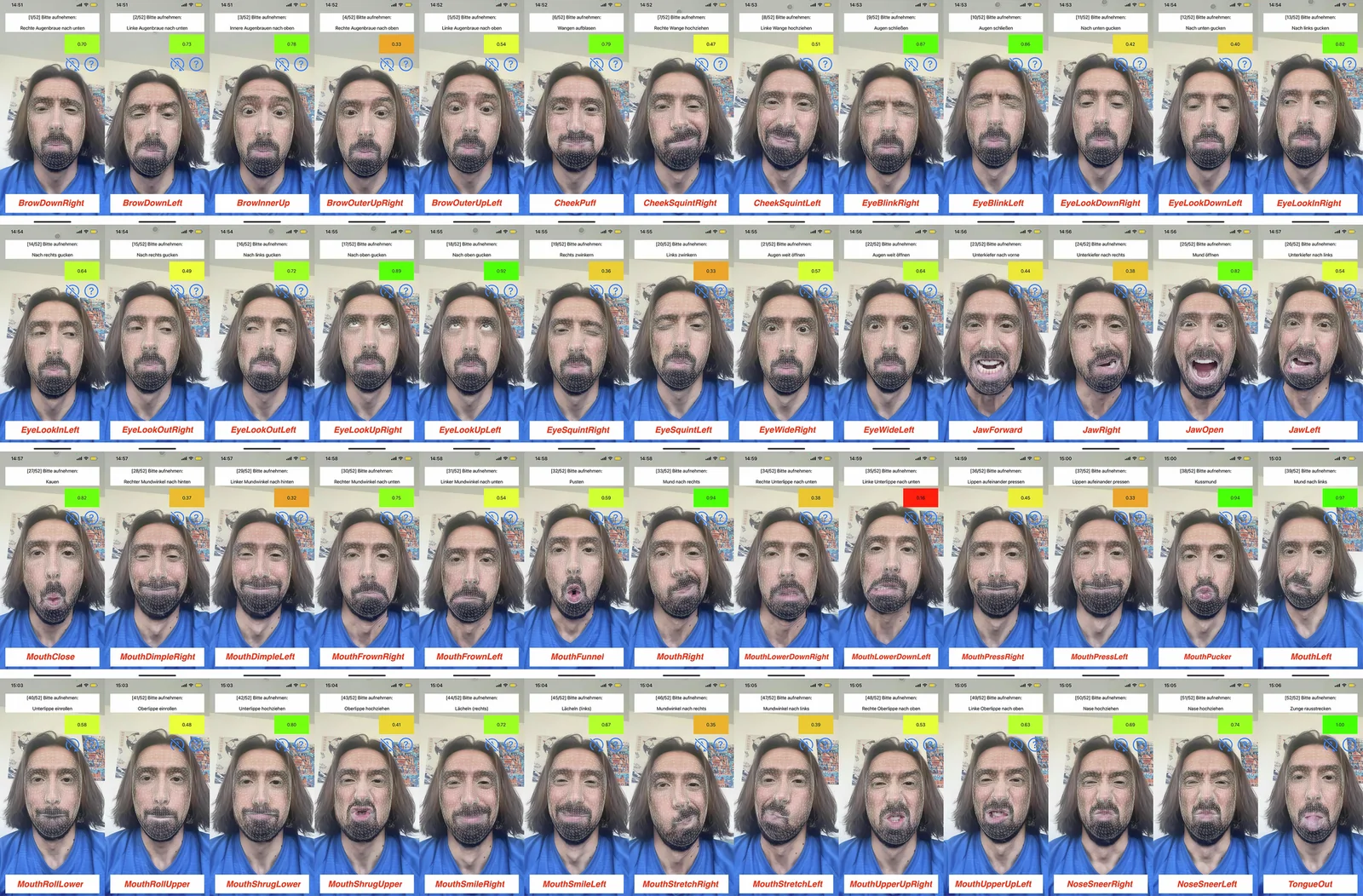
ExpressionTracker application interface. Each tile represents one facial blendshape; the value in the upper-right corner (0 to 1) denotes its coefficient, that is, the degree of deformation applied to the neutral facial mesh. Textual instructions for the corresponding facial movement are shown above each tile, while the animated mask cues used in the application are omitted here for conciseness. The interface imagery includes identifiable photographs of the first author (B.K.), who provided written informed consent for publication.

We used a custom iOS application adapted from Menzel et al. using the ARKit framework^23^. Participants performed 53 facial poses: one neutral pose and 52 expression poses corresponding to the predefined ARKit facial movements. The application continuously records numerical blendshape weights describing the extent to which each predefined movement is present at a given time. These coefficients are normalised (0 to 1) and represent proportional activation relative to each individual’s facial anatomy, providing person-specific calibration that accounts for baseline facial-structure variability. For each requested pose, the application automatically detects when the participant has reached the target movement most strongly and captures the complete set of blendshape weights at that moment (Fig. 7).

### Study design and participants

AVERT-MD (Avatar Evaluation in Real-Time for Movement Disorders) is a longitudinal observational study conducted within the InVirtuo 4.0 project. The present analysis reports baseline cross-sectional findings from the first assessment wave. Participants were recruited from the Clinic for Parkinson’s Disease, Sleep Disorders, and Movement Disorders at University Hospital Bonn. Inclusion criteria for PD required a clinical diagnosis according to the MDS Clinical Diagnostic Criteria for PD. PD-specific exclusion criteria were atypical parkinsonian syndromes (progressive supranuclear palsy, multiple system atrophy, corticobasal degeneration, dementia with Lewy bodies). HC-specific exclusion criteria were first-degree relatives with PD. Exclusion criteria for both groups were major psychiatric disorders (major depression, schizophrenia, bipolar disorder) and other neurological conditions affecting facial expressivity (facial palsy, hemifacial spasm, myasthenia gravis, myotonic dystrophy). The analysed sample comprised a matched cohort of 70 participants (34 PD, 36 HC). The study protocol was approved by the Institutional Review Board of University Hospital Bonn (approval number: 2024-367-BO). The study was conducted in accordance with the ethical principles of the Declaration of Helsinki. All participants provided written informed consent after receiving comprehensive information about the study procedures and sufficient time for consideration. Identifiable photographs of the first author (B.K.) appear in the ExpressionTracker interface and in the Supplementary Video; the first author has provided written informed consent for publication of these images and video (Fig. 7).

### Data acquisition protocol

Assessments were conducted in a standardised environment. Participants were seated at a fixed distance of 30 cm from a sixth-generation iPad Pro 12.9 inch equipped with a 12-megapixel ultra-wide front camera with TrueDepth sensors. Camera height was aligned with each participant’s eye level using a height-adjustable tripod. The room was illuminated exclusively by artificial fluorescent ceiling lighting, with outdoor light blocked by curtains. Participants removed eyeglasses to prevent reflections that could interfere with eye-related blendshape capture. After a brief introduction and a practice trial, data from the subsequent second trial were used for all analyses. Participants first maintained a neutral, relaxed expression to establish an individual baseline, then performed 52 voluntary facial movements guided by animated visual demonstrations paired with written instructions, with a four-second relaxation pause after each task (Supplementary Video).

### Blendshape conditions and feature engineering

From the 53 poses we derived three measurement conditions: the neutral condition (weights during the instructed neutral pose), the activated condition (weights at peak voluntary activation during each expression pose), and the delta condition (activated minus neutral, representing movement amplitude). This yielded 156 base features (52 blendshapes by three conditions). We then generated three classes of derived features. Aggregation features comprised five summary statistics (mean, standard deviation, maximum, minimum, and range) per condition, yielding 15 features. Interaction features were multiplicative pairwise products: we identified the 15 most discriminative base features using analysis-of-variance F-tests comparing PD and HC, then paired the top-ranked feature with the next five and iterated, yielding 30 interaction features. This global selection was performed once for the exploratory between-group and correlation analyses and was not repeated within cross-validation folds; the machine-learning pipeline instead refitted elastic-net feature selection within every inner training fold (see Machine-learning classification). Note that pairing was cross-task. No interaction term was computed from values sampled at the same time point in the same task. Asymmetry features were left-minus-right indices for all bilateral blendshape pairs (positive values indicate left-sided predominance), yielding 60 features across the three conditions. The complete engineered feature space comprised 261 features (156 base, 15 aggregation, 30 interaction, and 60 asymmetry) (Supplementary Fig. 1).

### Outlier detection

Outlier detection used the Tukey interquartile range (IQR) method, applied independently within each diagnostic group (HC and PD). On average, 4.1 outliers per feature were removed (approximately 5.8% of data points), balanced between HC (mean 2.0 per feature) and PD (mean 2.1 per feature). This procedure improved distributional normality: the proportion of features suitable for parametric testing increased from 26 to 57%.

### Coactivation and multicollinearity assessment

Given the synergistic nature of facial muscle activity, we assessed interdependence among blendshapes by examining pairwise correlations (|r| > 0.7) within each condition and by computing variance inflation factors (VIF) (Supplementary Figs. 2–4). Neutral blendshapes exhibited pronounced coactivation, including bilateral correlations reflecting facial symmetry (e.g ***eyeLookUpRight*** and ***eyeLookUpLeft***) and strong correlations among anatomically adjacent regions (e.g ***mouthUpperUp***, ***noseSneer***, and ***cheekSquint***). Voluntary facial movements disrupted this global coupling, though bilateral homologous correlations persisted at reduced levels. VIF analysis demonstrated substantial multicollinearity in the neutral condition that was markedly reduced in the activated and delta conditions.

### Statistical analyses

Normality was assessed with the Shapiro-Wilk test. Between-group analyses used the independent-samples t-test with Welch correction for normally distributed features and the two-tailed Mann–Whitney U test otherwise, with effect sizes expressed as Cohen’s d and rank-biserial correlation, respectively. Correlation analyses used the Pearson coefficient for normally distributed data and the Spearman coefficient otherwise. To control multiple testing within each of six feature families we applied a Bonferroni correction based on the effective number of independent tests (*Meff*) estimated from the eigenvalues of each family’s correlation matrix ; per-family *Meff* values are reported under "Multiple testing correction" section below and in ref. ^35^ (Supplementary Tables 2 and 3). Interdependence among blendshapes was assessed by pairwise correlations and variance inflation factors (Supplementary Table 1 and Supplementary Figs. 2–4). Five clinical outcome measures were analysed (MDS-UPDRS III item 3.2, MDS-UPDRS III sum, MDS-UPDRS IV sum, H&Y stage, and LED); correlations were adjusted for age, sex, and LED, except the LED correlation, which was adjusted for age, sex, and H&Y stage.

### Multiple testing correction

To account for dependency among correlated blendshapes, we applied a Bonferroni correction based on the effective number of independent tests (*Meff*), estimated from the eigenvalue spectrum of each feature family’s correlation matrix^35^. Rather than treating all 261 features as a single test family, which would be overly conservative, we performed hypothesis testing within six functional feature families (neutral, activated, delta, aggregation, interaction, and asymmetry), each representing a distinct biological hypothesis (for example, whether voluntary movement amplitude is altered in PD, addressed by delta features, versus whether facial asymmetry is altered, addressed by asymmetry features). We note that this within-family correction is more lenient than a single global correction across all 261 features: it applies a smaller effective number of tests within each family and therefore a less stringent per-test threshold. The per-family *Meff* values and the resulting Bonferroni-adjusted thresholds (alpha = 0.05/*Meff*) are reported for the between-group and correlation analyses (Supplementary Table 2 and Supplementary Table 3).

For the between-group analysis:

- Delta: 52 features, Meff = 50.46, adjusted alpha = 1.0 × 10^−3^
- Neutral: 52 features, Meff = 48.08, adjusted alpha = 1.0 × 10^−3^
- Activated: 52 features, Meff = 50.62, adjusted alpha = 1.0 × 10^−3^
- Aggregation: 15 features, Meff = 8.60, adjusted alpha = 5.8 × 10^−3^
- Interaction: 30 features, Meff = 28.60, adjusted alpha = 1.7 × 10^−3^
- Asymmetry: 60 features, Meff = 48.64, adjusted alpha = 1.0 × 10^−3^

For the correlation analysis:

- Delta: 52 features, Meff = 37.50, adjusted alpha = 1.3 × 10^−3^
- Neutral: 52 features, Meff = 36.28, adjusted alpha = 1.4 × 10^−3^
- Activated: 52 features, Meff = 38.87, adjusted alpha = 1.3 × 10^−3^
- Aggregation: 15 features, Meff = 7.99, adjusted alpha = 6.3 × 10^−3^
- Interaction: 30 features, Meff = 24.01, adjusted alpha = 2.1 × 10^−3^
- Asymmetry: 60 features, Meff = 41.96, adjusted alpha = 1.2 × 10^−3^

### Machine-learning pipeline

We focused the classification analysis on the diagnostic task, PD versus HC, in the single matched cohort of 70 participants (34 PD, 36 HC). Feature selection used an embedded elastic-net logistic model^36^. All models were evaluated under nested cross-validation: an outer loop of stratified five-fold cross-validation repeated ten times (50 outer folds) estimated generalisation performance, while an inner stratified cross-validation performed grid-search hyperparameter tuning. Every data-dependent step, including residualisation for age and sex, median imputation, robust scaling, elastic-net feature selection, and hyperparameter search, was fitted only on the inner training data of each fold and then applied to the held-out data, so that no information from held-out participants informed model fitting. Because each participant appeared in exactly one held-out outer test fold per repeat, every participant contributed ten out-of-fold predicted probabilities. For each participant we summed these probabilities and divided by ten to obtain a single pooled out-of-fold score; all reported discrimination metrics (AUC with bootstrap 95% confidence intervals, accuracy, balanced accuracy, sensitivity, specificity, and F1 score) and the ROC curves in (Fig. 3) were computed from these pooled scores across all 70 participants. We evaluated eight classifiers: logistic regression (LR), random forest (RF), gradient boosting machine (GBM), support vector machine (SVM) with a radial basis function kernel, multilayer perceptron (MLP), extreme gradient boosting (XGBoost), light gradient boosting machine (LightGBM), and categorical boosting (CatBoost). Class imbalance was handled consistently by class weighting, the decision threshold was fixed at 0.5 for all models, and PD was the positive class. A single global random seed (42) was used throughout. To describe the most important features, we computed out-of-fold SHAP values aggregated across the 50 outer test folds^37^. Statistical significance of the observed performance was assessed by a label-permutation test with 1000 shuffles in which the labels were randomly shuffled and the per-fold feature selection was refitted on every shuffle; the empirical *p* value is the proportion of permutations whose AUC matched or exceeded the observed AUC (Supplementary Fig. 5). Full hyperparameter configurations and software versions are provided in the following two subsections.

### Feature selection and hyperparameter configurations

Feature selection used SelectFromModel with an elastic-net logistic regression (saga solver, C = 0.5, class weighting balanced, maximum 1000 iterations), retaining the top features by absolute coefficient; the elastic-net mixing parameter (l1_ratio in {0.2, 0.8}) and the number of retained features (in {20, 40}) were tuned in the inner loop^36^. The per-model hyperparameter grids tuned in the inner loop were:

- Logistic regression (LR): C in {0.01, 0.1, 1.0, 10.0}.
- Random forest (RF): maximum depth in {3, 5, 15, none}; maximum features = sqrt; 300 trees.
- Gradient boosting machine (GBM): learning rate in {0.05, 0.1}; maximum depth in {2, 3}.
- Support vector machine (SVM): C in {0.1, 1.0, 10.0}; gamma = scale; radial basis function kernel; probability estimates enabled.
- Multilayer perceptron (MLP): hidden layer sizes in {(100, 50), (50,)}; L2 penalty alpha in {1 × 10^−4^, 1 × 10^−3^}.
- XGBoost: maximum depth in {3, 6}; learning rate in {0.05, 0.1}.
- LightGBM: maximum depth in {3, 6}; learning rate in {0.05, 0.1}.
- CatBoost: depth in {4, 6}; learning rate in {0.05, 0.1}.

Model interpretation used out-of-fold SHAP (SHapley Additive exPlanations) values for the best-performing model (GBM), aggregated across the 50 outer test folds^37^. Statistical significance of the observed performance was assessed by a label-permutation test with 1000 shuffles in which the diagnostic labels were randomly shuffled and the per-fold feature selection was refitted on every shuffle; the empirical *p* value is the proportion of permutations whose AUC matched or exceeded the observed AUC (Supplementary Fig. 5). Three reference feature sets were run through the identical pipeline for comparison: an amplitude-only baseline (the 52 delta blendshapes), a demographic confounder-only baseline (age and sex), and an engineered OpenFace action-unit set computed on the same cohort (Supplementary Fig. 6).

### Software and computational environment

All analyses were implemented in Python 3.12. Machine learning and statistics: scikit-learn 1.5 (pipelines, cross-validation, elastic-net selection, metrics), XGBoost 2.1, LightGBM 4.5, CatBoost 1.2, SHAP 0.46, and statsmodels 0.14. Data processing and numerical computing: pandas 2.2, NumPy 2.x, and SciPy 1.14. Visualisation: matplotlib 3.9 and seaborn 0.13. The analysis pipeline fixes a single global random seed (42) for every estimator, data splitter, permutation, and bootstrap to ensure reproducibility. Agentic coding assistance (code generation, refactoring, and support for result checking) was provided by Cursor, with all analyses verified by the authors.

## Supplementary information

Supplementary information

Supplementary video

## Data Availability

The minimal dataset underlying the findings of this study comprises participant-level facial blendshape features and clinical covariates. These data cannot be made publicly available owing to patient privacy constraints and institutional ethics restrictions. De-identified data may be available from the corresponding author upon reasonable request, subject to institutional approval and a data-use agreement.

## Code availability

The custom analysis pipeline used to generate and analyse the datasets reported in this article is available from the corresponding author upon reasonable request.
