# Supplementary material for: Interaction- and asymmetry-aware facial blendshape analysis for objective quantification of Parkinsonian hypomimia
Source: NPJ Parkinsons Dis. 2026 Sep 23;12:231. doi: 10.1038/s41531-026-01579-2 (PMC13601575; doi:10.1038/s41531-026-01579-2)
Supplement: Supplementary file 1 [file 41531_2026_1579_MOESM1_ESM.pdf]

Interaction- and Asymmetry-Aware Facial Blendshape Analysis for Objective Quantification of Parkinsonian Hypomimia

SUPPLEMENTARY INFORMATION

Supplementary Information

Supplementary Information 1: Facial Action Coding System (FACS)

The ExpressionTracker shares fundamental principles with the Facial Action Coding System (FACS) and its action units, as both frameworks describe facial movement as anatomically grounded transformations of the facial structure. FACS was introduced in the 1970s by Paul Ekman to analyse facial expressions across six main emotion categories (happiness, sadness, anger, fear, surprise, and disgust) by decomposing them into anatomically defined building blocks termed action units, each corresponding to the activation or relaxation of a single facial muscle or a functionally inseparable muscle group **[1]**. Computer-graphics researchers later adopted the same action units to parameterise and synthesise facial expressions of virtual humans. Face-tracking software from the computer-graphics and computer-vision communities now uses analysis-by-synthesis approaches to track human facial expressions, providing stable detection of action units (or blendshapes) on consumer devices such as smartphones, opening avenues beyond emotional interpretation toward holistic motor quantification of facial expressions.

Supplementary Information 2: MDS-UPDRS IV correlation analysis

No facial blendshape feature survived *Meff*-adjusted Bonferroni correction for the MDS-UPDRS IV sum score. This selective absence of significant associations suggests that facial blendshapes reflect parkinsonian motor state rather than treatment-related motor complications such as dyskinesias or motor fluctuations.

Supplementary Figures

Supplementary Figure 1 | Apple ARKit facial blendshapes and feature-engineering pipeline

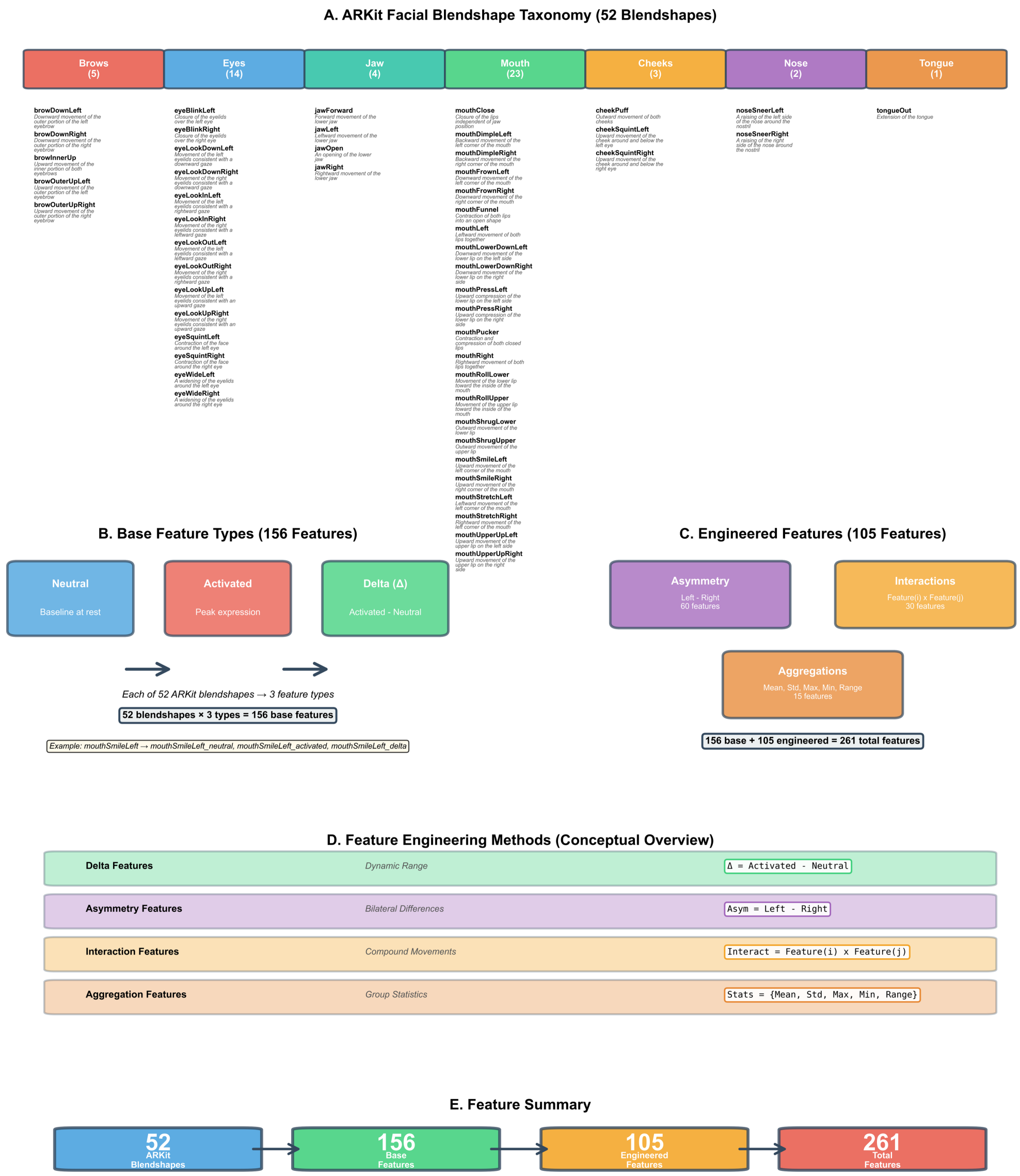

A: Apple's ARKit framework provides 52 facial blendshapes via the TrueDepth camera, each representing a discrete facial action unit quantified as a continuous value between 0 and 1.

B: Each blendshape is represented by neutral (baseline at rest), activated (peak expression during task performance), and delta (dynamic range = Activated − Neutral), yielding 156 base features (52 blendshapes × 3 conditions).

C: Engineered features extend the base representation with bilateral asymmetry (left − right), multiplicative interactions of top discriminative blendshapes, and per-group aggregations (mean, standard deviation, max, min, range), adding 105 features for downstream analysis.

D: Feature engineering applies defined transformations—delta as activated−neutral dynamic range, asymmetry as bilateral differences, interactions as compound movement products, and aggregations as summary statistics across feature groups.

E: Feature summary showing transformation from 52 blendshapes to 261 total features

Supplementary Figure 2 | Correlation matrix: activated blendshapes

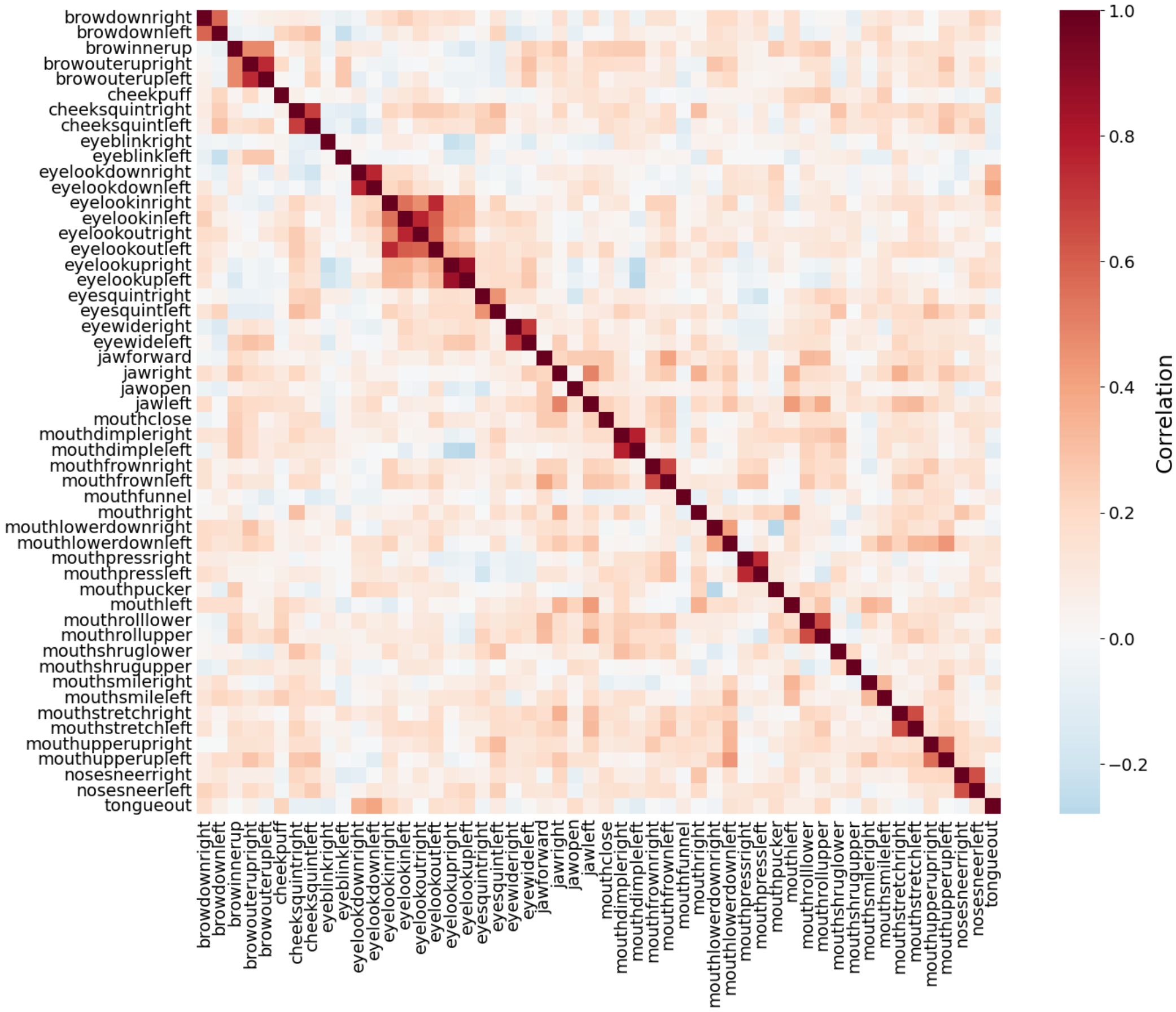

Correlation matrices show pairwise Pearson correlations between RAW facial blendshape features within each condition (Activated, Neutral, Delta). Condition-specific suffixes are removed from labels for readability. Highly collinear pairs (|r| ≥ 0.70): eyelookupright–eyelookupleft (r=0.85); mouthdimpleright–mouthdimpleleft (r=0.78); eyelookdownright–eyelookdownleft (r=0.76); eyelookinleft–eyelookoutright (r=0.76); mouthpressright–mouthpressleft (r=0.75); eyelookinright–eyelookoutleft (r=0.75); browouterupright–browouterupleft (r=0.74); eyewideright–eyewideleft (r=0.70).

Supplementary Figure 3 | Correlation matrix: neutral blendshapes

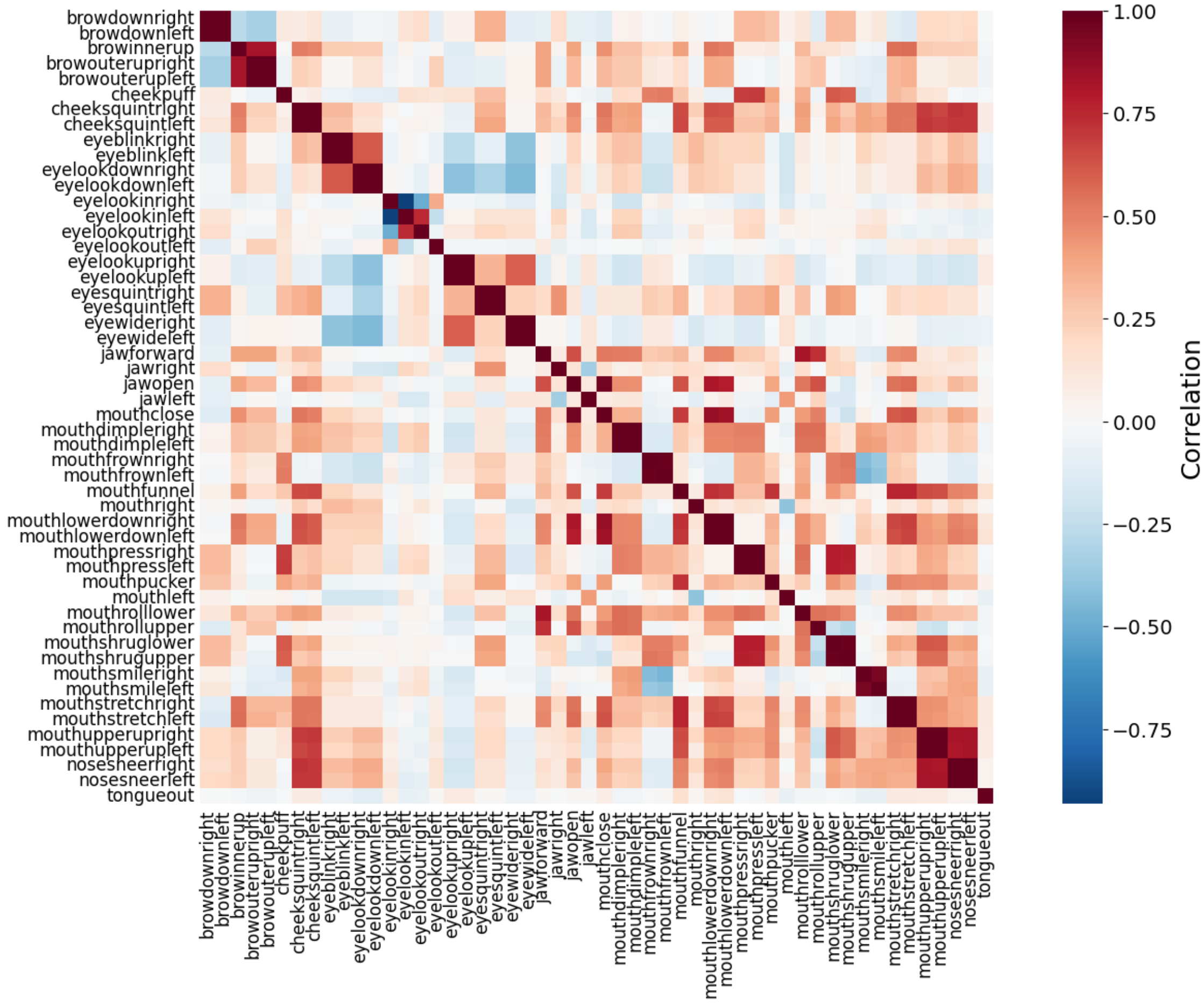

Correlation matrices show pairwise Pearson correlations between RAW facial blendshape features within each condition (Activated, Neutral, Delta). Condition-specific suffixes are removed from labels for readability. Highly collinear pairs (|r| ≥ 0.70): eyesquintright–eyesquintleft (r=1.00); eyeblinkright–eyeblinkleft (r=1.00); eyelookdownright–eyelookdownleft (r=1.00); browdownright–browdownleft (r=1.00); eyelookupright–eyelookupleft (r=1.00); browouterupright–browouterupleft (r=1.00); eyewideright–eyewideleft (r=1.00); mouthpressright–mouthpressleft (r=1.00); mouthdimpleright–mouthdimpleleft (r=1.00); mouthlowerdownright–mouthlowerdownleft (r=0.99); nosesneerright–nosesneerleft (r=0.99); mouthstretchright–mouthstretchleft (r=0.99); cheeksquintright–cheeksquintleft (r=0.99); mouthupperupright–mouthupperupleft (r=0.99); mouthfrownright–mouthfrownleft (r=0.98); mouthshruglower–mouthshrugupper (r=0.98); jawopen–mouthclose (r=0.96); mouthsmileright–mouthsmileleft (r=0.94); eyelookinright–eyelookinleft (r=-0.93); mouthclose–mouthlowerdownright (r=0.86); mouthclose–mouthlowerdownleft (r=0.84); browinnerup–browouterupleft (r=0.83); browinnerup–browouterupright (r=0.83); mouthupperupright–nosesneerleft (r=0.83); mouthupperupright–nosesneerright (r=0.83); jawforward–mouthrolllower (r=0.83); mouthupperupleft–nosesneerleft (r=0.82); jawopen–mouthlowerdownright (r=0.82); mouthupperupleft–nosesneerright (r=0.80); mouthpressright–mouthshruglower (r=0.79); jawopen–mouthlowerdownleft (r=0.79); mouthpressleft–mouthshruglower (r=0.78); mouthpressright–mouthshrugupper (r=0.78); mouthpressleft–mouthshrugupper (r=0.77); mouthfunnel–mouthstretchright (r=0.76); mouthfunnel–mouthstretchleft (r=0.76); eyelookinleft–eyelookoutright (r=0.74); jawforward–mouthrollupper (r=0.72); mouthfunnel–mouthlowerdownright (r=0.72); mouthfunnel–mouthpucker (r=0.72); cheeksquintright–nosesneerright (r=0.72); cheeksquintleft–nosesneerright (r=0.70); cheeksquintright–nosesneerleft (r=0.70); mouthfunnel–mouthlowerdownleft (r=0.70); cheeksquintleft–mouthupperupright (r=0.70).

Supplementary Figure 4 | Correlation matrix: delta blendshapes

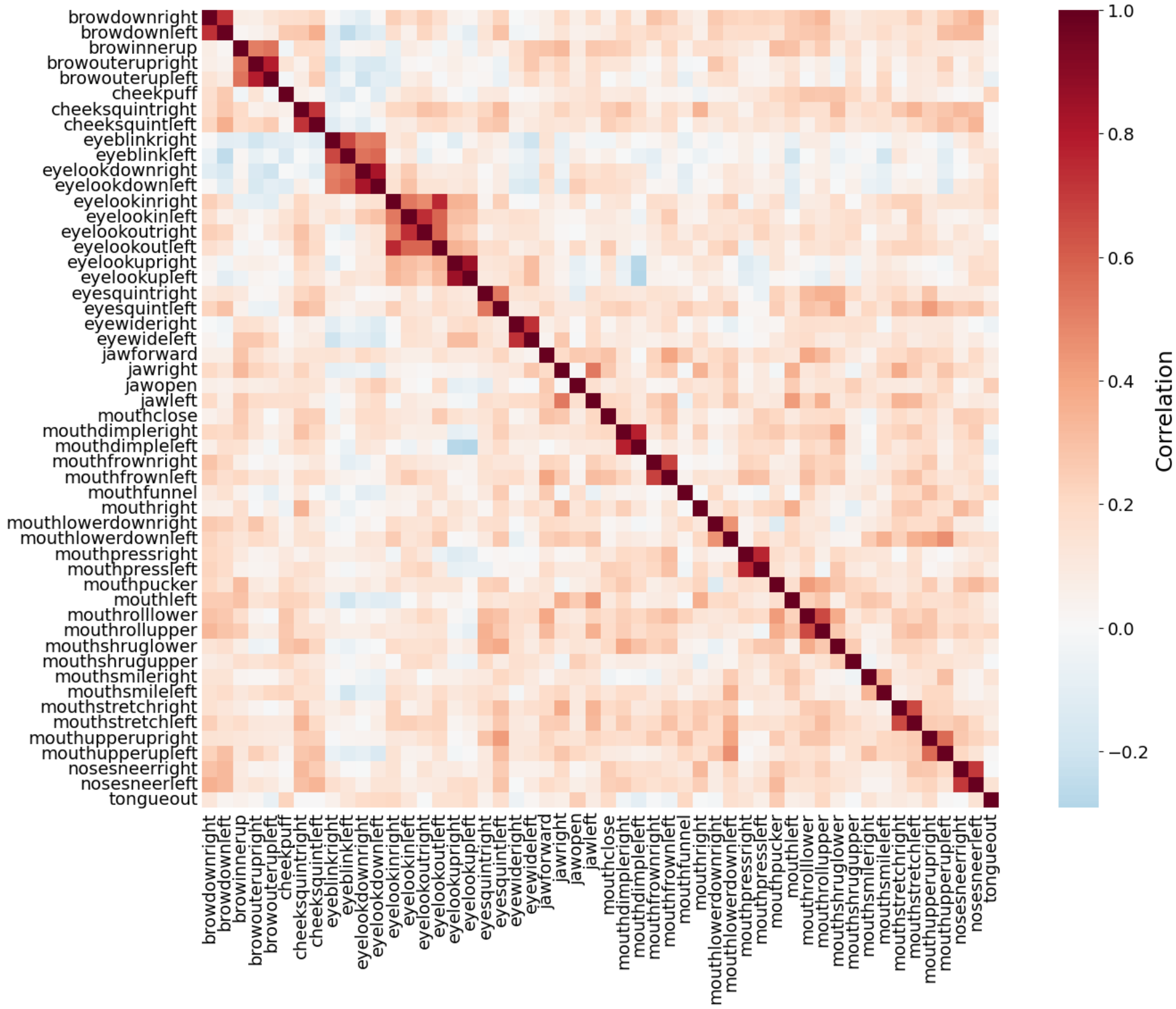

Correlation matrices show pairwise Pearson correlations between RAW facial blendshape features within each condition (Activated, Neutral, Delta). Condition-specific suffixes are removed from labels for readability. Highly collinear pairs (|r| ≥ 0.70): eyelookupright–eyelookupleft (r=0.85); eyelookdownright–eyelookdownleft (r=0.82); browouterupright–browouterupleft (r=0.78); mouthdimpleright–mouthdimpleleft (r=0.78); mouthpressright–mouthpressleft (r=0.76); eyelookinright–eyelookoutleft (r=0.75); eyelookinleft–eyelookoutright (r=0.74); eyewideright–eyewideleft (r=0.73); browdownright–browdownleft (r=0.73); cheeksquintright–cheeksquintleft (r=0.72); nosesneerright–nosesneerleft (r=0.71).

Supplementary Figure 5 | Label-permutation test for the PD versus HC pipeline

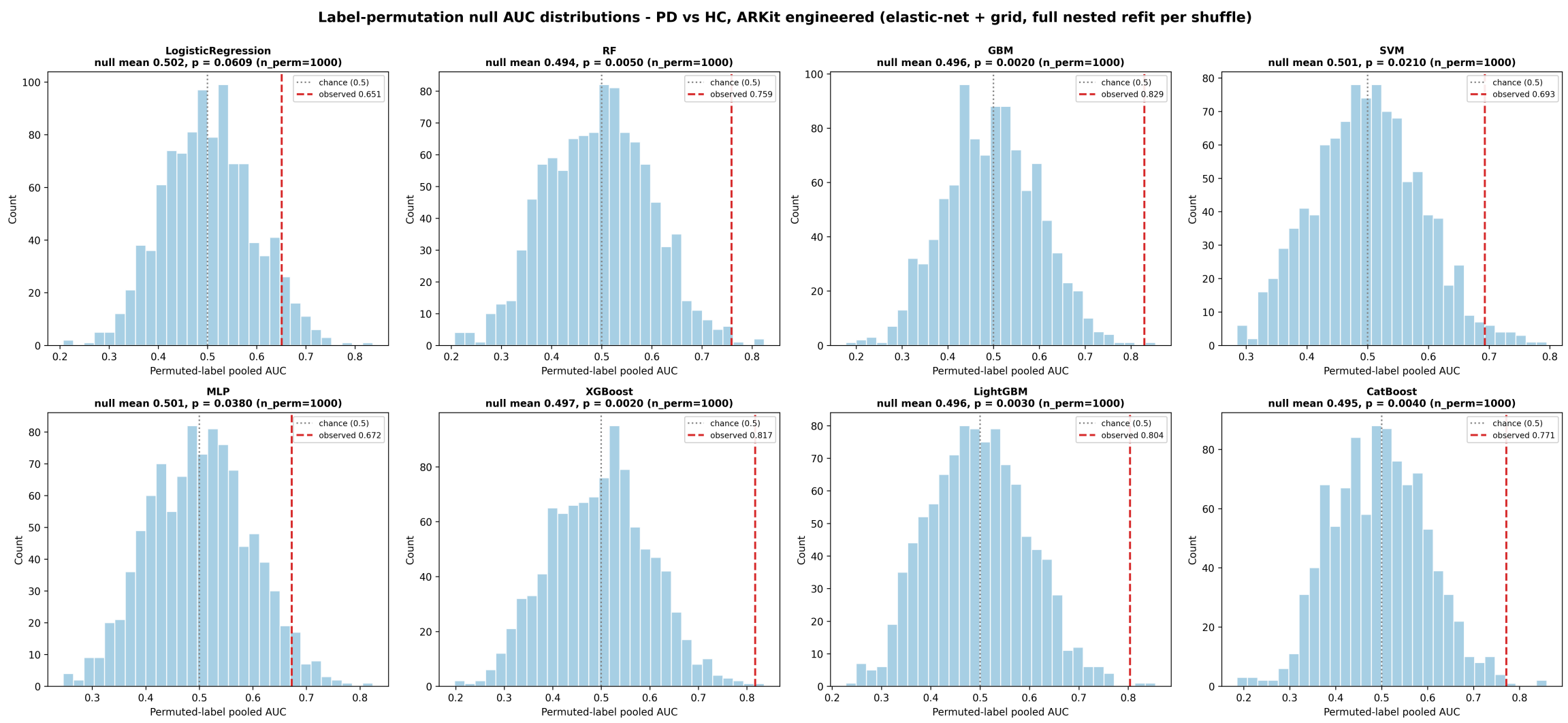

Supplementary Figure 5. Label-permutation test for the PD versus HC pipeline. For each classifier, the boxplot shows the distribution of out-of-fold AUCs obtained when the diagnostic labels were randomly shuffled (the null distribution), with the per-fold feature selection refitted on every shuffle; the green diamond marks the observed AUC and the dashed line marks chance (AUC 0.5). The empirical p value is the proportion of permutations whose AUC matched or exceeded the observed value. This analysis used 1000 permutations. The observed AUCs exceeded their null distributions for seven of the eight classifiers (GBM empirical p = 0.002; XGBoost 0.002; LightGBM 0.003; CatBoost 0.004; RF 0.005; SVM 0.021; MLP 0.038); logistic regression did not reach significance (empirical p = 0.061). Abbreviations: AUC, area under the receiver operating characteristic curve; PD, Parkinson's disease; HC, healthy controls; other model abbreviations as in Supplementary Figure 6.

Supplementary Figure 6 | Diagnostic performance across feature sets

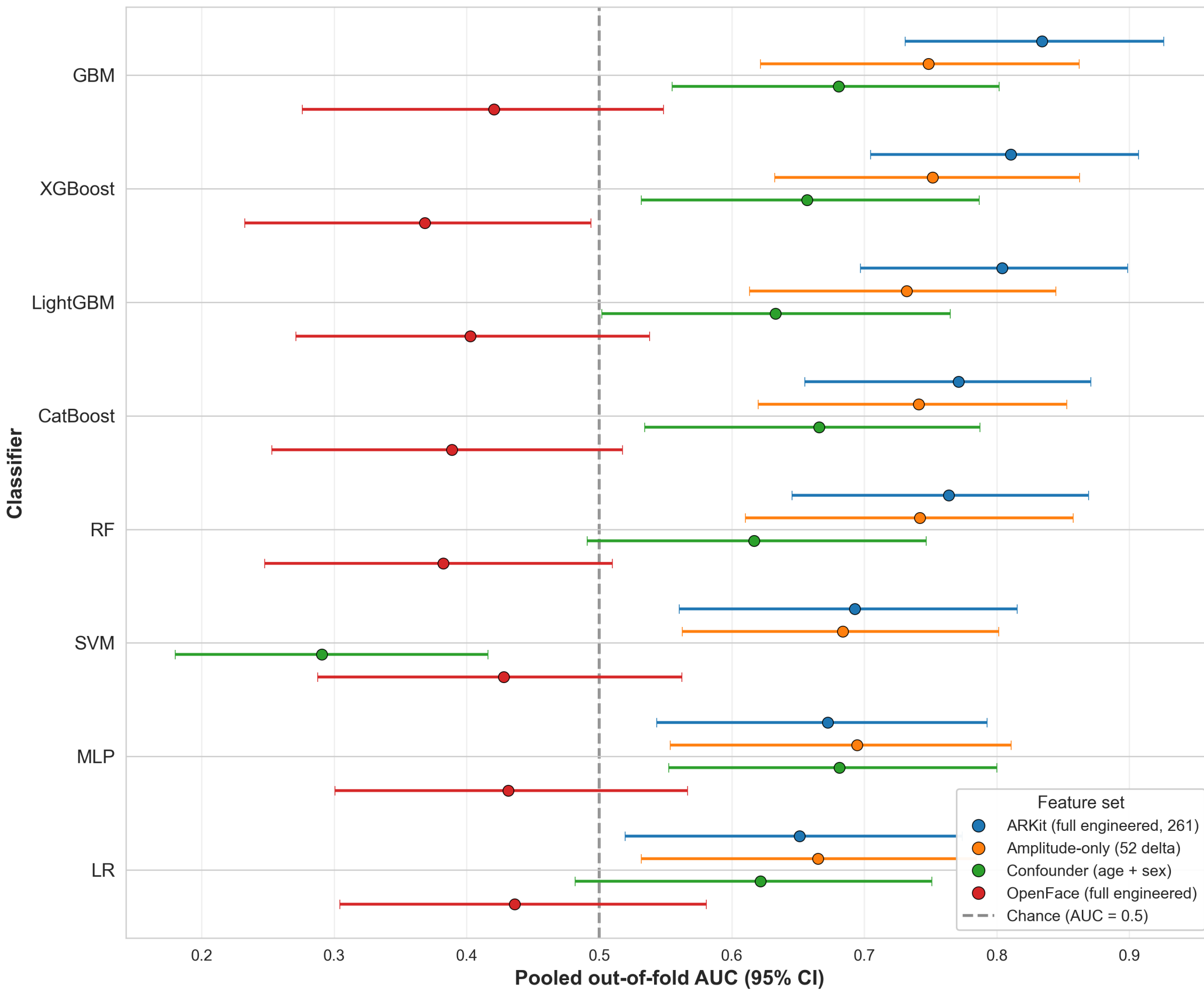

Supplementary Figure 6. Forest plot of diagnostic discrimination (area under the receiver operating characteristic curve, AUC) for the eight classifiers across four feature sets in the matched PD versus HC cohort (34 PD, 36 HC). Points are pooled out-of-fold AUCs under nested cross-validation (outer five-fold cross-validation repeated ten times); horizontal bars are bootstrap 95 percent confidence intervals. Feature sets are the engineered ARKit pool, an amplitude-only baseline (52 delta blendshapes), a demographic confounder-only baseline (age and sex), and an engineered OpenFace action-unit set on the same ARKit-selected frames. The vertical dashed line marks chance (AUC 0.5). Across all eight classifiers the engineered ARKit features ranked highest, the amplitude-only and confounder-only baselines were intermediate, and the OpenFace set fell at or below chance. The best model per feature set reached pooled out-of-fold AUCs of 0.834 (GBM; 95 percent CI 0.733 to 0.923) for the engineered ARKit pool, 0.752 (XGBoost; 95 percent CI 0.632 to 0.862) for the amplitude-only baseline, 0.681 (GBM and MLP; 95 percent CI 0.555 to 0.802 and 0.552 to 0.800, respectively) for the confounder-only baseline, and 0.436 (logistic regression; 95 percent CI 0.304 to 0.581) for the OpenFace set. Abbreviations: AUC, area under the receiver operating characteristic curve; PD, Parkinson's disease; HC, healthy controls; GBM, gradient boosting machine; XGBoost, extreme gradient boosting; LightGBM, light gradient boosting machine; CatBoost, categorical boosting; RF, random forest; SVM, support vector machine; MLP, multilayer perceptron; LR, logistic regression.

Supplementary Tables

Supplementary Table 1| Variance inflation factors for ARKit blendshapes

Condition	Blendshape	VIF
Activated	tongueout	537.92
Activated	eyeblinkleft	199.62
Activated	mouthpucker	110.02
Activated	eyelookoutleft	109.1
Activated	eyelookupright	108.91
Activated	eyelookinleft	106.9
Activated	eyelookinright	97.07
Activated	eyelookupleft	95.24
Activated	eyelookdownleft	85.9
Activated	eyelookoutright	69.95
Activated	eyeblinkright	64.02
Activated	jawopen	63.04
Activated	browouterupleft	62.27
Activated	eyelookdownright	55.66
Activated	eyewideleft	54.66
Activated	browouterupright	48.66
Activated	eyewideright	46.83
Activated	nosesneerleft	39.36
Activated	mouthsmileright	32.68
Activated	browdownleft	31.36
Activated	cheeksquintright	31.33
Activated	browinnerup	31.16
Activated	browdownright	30.88
Activated	nosesneerright	30.37
Activated	mouthrolllower	27.95

Activated	eyesquintleft	27.51
Activated	cheeksquintleft	27.2
Activated	eyesquintright	26.61
Activated	mouthlowerdownleft	26.37
Activated	mouthupperupright	26.21
Activated	mouthsmileleft	25.28
Activated	cheekpuff	23.62
Activated	mouthfunnel	22.07
Activated	mouthupperupleft	21.27
Activated	mouthclose	20.17
Activated	mouthleft	19.4
Activated	mouthshruglower	18.39
Activated	mouthpressleft	17.27
Activated	mouthpressright	16.34
Activated	mouthlowerdownright	16.32
Activated	mouthright	14.73
Activated	mouthstretchleft	14.47
Activated	mouthstretchright	13.43
Activated	mouthdimpleleft	12.63
Activated	mouthdimpleright	12.51
Activated	mouthfrownleft	11.54
Activated	jawleft	10.74
Activated	mouthfrownright	10.62
Activated	mouthrollupper	10.57
Activated	jawright	8.52
Activated	mouthshrugupper	8.2
Activated	jawforward	5.3
Neutral	eyesquintleft_neutral	8147345.35
Neutral	eyesquintright_neutral	8146425.43

Neutral	eyelookdownright_neutral	1499961.1
Neutral	eyelookdownleft_neutral	1498677.47
Neutral	eyeblinkleft_neutral	1081570.69
Neutral	eyeblinkright_neutral	1081487.7
Neutral	browdownleft_neutral	700614.02
Neutral	browdownright_neutral	698977.99
Neutral	eyewideleft_neutral	184704.96
Neutral	eyewideright_neutral	184402.08
Neutral	eyelookupright_neutral	162530.39
Neutral	eyelookupleft_neutral	162177.13
Neutral	browouterupleft_neutral	97324.7
Neutral	browouterupright_neutral	97127.49
Neutral	mouthpressleft_neutral	3693.49
Neutral	mouthpressright_neutral	3692.19
Neutral	mouthdimpleright_neutral	1842.53
Neutral	mouthdimpleleft_neutral	1793.93
Neutral	mouthstretchleft_neutral	1638.85
Neutral	mouthstretchright_neutral	1543.53
Neutral	cheeksquintright_neutral	1400.78
Neutral	nosesneerright_neutral	1297.79
Neutral	cheeksquintleft_neutral	1288.15
Neutral	mouthlowerdownright_neutral	1076.64
Neutral	nosesneerleft_neutral	1069.58
Neutral	mouthlowerdownleft_neutral	949.26
Neutral	mouthupperupright_neutral	644.96
Neutral	mouthupperupleft_neutral	568.71
Neutral	mouthshruglower_neutral	458.52
Neutral	mouthshrugupper_neutral	304.82
Neutral	jawopen_neutral	225.25

Neutral	mouthfrownleft_neutral	199.98
Neutral	mouthfrownright_neutral	198.06
Neutral	mouthclose_neutral	197.67
Neutral	mouthsmileright_neutral	83.06
Neutral	mouthsmileleft_neutral	75.87
Neutral	mouthrolllower_neutral	49.3
Neutral	mouthfunnel_neutral	45.59
Neutral	jawforward_neutral	36.08
Neutral	mouthrollupper_neutral	32.39
Neutral	browinnerup_neutral	26.46
Neutral	eyelookinleft_neutral	25.64
Neutral	mouthpucker_neutral	20.19
Neutral	cheekpuff_neutral	15.73
Neutral	eyelookinright_neutral	10.47
Neutral	mouthright_neutral	9.33
Neutral	jawright_neutral	6.01
Neutral	mouthleft_neutral	5.82
Neutral	jawleft_neutral	5.42
Neutral	eyelookoutright_neutral	4.02
Neutral	eyelookoutleft_neutral	2.7
Neutral	tongueout_neutral	1.54
Delta	tongueout_delta	175.84
Delta	eyelookupright_delta	109.4
Delta	eyelookoutleft_delta	109.01
Delta	eyelookupleft_delta	91.05
Delta	mouthpucker_delta	88.66
Delta	eyelookinright_delta	87.85
Delta	eyelookinleft_delta	74.21
Delta	eyeblinkleft_delta	68.41

Delta	eyelookoutright_delta	67.97
Delta	browouterupleft_delta	57.62
Delta	jawopen_delta	57.4
Delta	eyewideleft_delta	50.19
Delta	browouterupright_delta	47.78
Delta	eyewideright_delta	45.32
Delta	eyeblinkright_delta	43.49
Delta	eyelookdownleft_delta	41.68
Delta	eyelookdownright_delta	29.17
Delta	mouthsmileright_delta	26
Delta	nosesneerleft_delta	25.72
Delta	mouthupperupright_delta	24.92
Delta	browinnerup_delta	24.46
Delta	mouthsmileleft_delta	22.78
Delta	cheekpuff_delta	22.29
Delta	cheeksquintright_delta	20.92
Delta	mouthlowerdownleft_delta	20.68
Delta	nosesneerright_delta	20.57
Delta	mouthupperupleft_delta	19.62
Delta	cheeksquintleft_delta	18.92
Delta	eyesquintleft_delta	18.79
Delta	browdownleft_delta	18.23
Delta	mouthleft_delta	18.05
Delta	mouthfunnel_delta	17.95
Delta	eyesquintright_delta	17.95
Delta	mouthclose_delta	17.74
Delta	browdownright_delta	16.08
Delta	mouthrolllower_delta	16
Delta	mouthright_delta	14.87

Delta	mouthlowerdownright_delta	12.52
Delta	mouthpressleft_delta	12.24
Delta	jawleft_delta	11.5
Delta	mouthpressright_delta	11.41
Delta	mouthshruglower_delta	11.27
Delta	mouthdimpleleft_delta	9.8
Delta	jawright_delta	9.39
Delta	mouthfrownright_delta	9.14
Delta	mouthrollupper_delta	9.01
Delta	mouthdimpleright_delta	8.96
Delta	mouthfrownleft_delta	8.67
Delta	mouthstretchleft_delta	7.59
Delta	mouthstretchright_delta	7.09
Delta	mouthshrugupper_delta	5.24
Delta	jawforward_delta	4.36

Supplementary Table 1. Variance inflation factors (VIF) for the 52 Apple ARKit facial blendshapes under each measurement condition (activated, neutral, and delta). VIF quantifies multicollinearity as the inflation of the sampling variance of a regression coefficient relative to the orthogonal case; larger values indicate stronger linear dependence on the remaining blendshapes. Values were computed within condition after Tukey outlier removal.

Supplementary Table 2 | Effective number of tests for between-group analyses

Feature group	N features	*Meff*	p_adj threshold
Delta	52	50.46	9.91e-04
Neutral	52	48.08	0.001
Activated	52	50.62	9.88e-04
Aggregation	15	8.6	0.0058
Interaction	30	28.6	0.0017
Asymmetry	60	48.64	0.001

Supplementary Table 2. *Meff*-based multiple-testing correction for the PD versus HC between-group analyses. Hypothesis testing was performed within six feature families (delta, neutral, activated, aggregation, interaction, and asymmetry). For each family, the effective number of independent tests (*Meff*) was estimated from the eigenvalue spectrum of the within-family correlation matrix, and a Bonferroni threshold was set at alpha = 0.05 / *Meff*.

Supplementary Table 3 | Effective number of tests for clinical correlation analyses

Feature group	N features	*Meff*	p_adj threshold
Delta	52	37.5	0.0013
Neutral	52	36.28	0.0014
Activated	52	38.87	0.0013
Aggregation	15	7.99	0.0063
Interaction	30	24.01	0.0021
Asymmetry	60	41.96	0.0012

Supplementary Table 3. *Meff*-based multiple-testing correction for the clinical correlation analyses in people with PD. Correlations were tested within the same six feature families used for the between-group analyses. *Meff* was estimated from the eigenvalue spectrum of each family's correlation matrix, and the Bonferroni threshold was set at alpha = 0.05 / *Meff*.

Supplementary Table 4 | Complete between-group comparisons of facial blendshape features (PD versus HC)

Feature	Feature group	HC mean	HC SD	PD mean	PD SD	Test	p value	Effect size	Effect type	p_adj
mouthlowerdownright_delta	Delta	0.049	0.145	-0.126	0.103	MWU	6.74e-07	-0.72	Rank_biserial	3.40e-05
mouthlowerdownright	Activated	0.034	0.135	-0.1	0.113	MWU	1.03e-04	-0.57	Rank_biserial	0.0052
eyewideright_delta × mouthlowerdownright_delta	Interaction	0.00562	0.011	-0.00968	0.02	t-test	3.29e-04	-0.95	Cohens_d	0.00941
browouterupleft_neutral	Neutral	-0.02	0.03	7.11e-04	0.00542	MWU	3.35e-04	0.57	Rank_biserial	0.016
browouterupright_neutral	Neutral	-0.02	0.03	7.34e-04	0.00541	MWU	3.35e-04	0.57	Rank_biserial	0.016
asym_mouthfrownleft	Asymmetry	-0.063	0.106	0.06	0.174	t-test	9.77e-04	0.85	Cohens_d	0.048
eyewideright	Activated	-0.042	0.17	0.081	0.087	MWU	0.00143	0.45	Rank_biserial	0.072
eyewideright_delta	Delta	-0.04	0.182	0.077	0.107	t-test	0.00188	0.77	Cohens_d	0.095
asym_mouthfrownleft_delta	Asymmetry	-0.057	0.116	0.05	0.158	t-test	0.00254	0.78	Cohens_d	0.124

eyelookoutleft_neutral	Neutral	-6.93e-05	6.41e-05	-3.52e-05	6.12e-05	MWU	0.00392	0.4	Rank_biserial	0.188
asym_eyelookinleft_delta	Asymmetry	0.043	0.128	-0.055	0.148	t-test	0.00432	-0.71	Cohens_d	0.21
mouthshrugupper_delta × eyelookdownright	Interaction	-0.00153	0.00401	0.0028	0.0053	MWU	0.00449	0.42	Rank_biserial	0.128
eyeblinkleft_delta	Delta	-0.011	0.12	0.067	0.051	MWU	0.00484	0.41	Rank_biserial	0.244
eyelookdownleft	Activated	0.023	0.059	-0.023	0.077	MWU	0.00506	-0.4	Rank_biserial	0.256
mouthfunnel_delta	Delta	0.026	0.125	-0.046	0.09	t-test	0.0084	-0.65	Cohens_d	0.424
eyesquintright_delta × eyewideright_delta	Interaction	0.00714	0.015	-6.46e-04	0.00898	t-test	0.016	-0.61	Cohens_d	0.463
browinnerup_neutral	Neutral	-0.033	0.031	-0.014	0.03	t-test	0.017	0.62	Cohens_d	0.806
eyelookdownright_delta	Delta	0.035	0.091	-0.018	0.088	t-test	0.017	-0.6	Cohens_d	0.852
eyelookdownleft_delta	Delta	0.032	0.077	-0.013	0.08	t-test	0.02	-0.58	Cohens_d	1
mouthstretchright_neutral	Neutral	-0.015	0.036	0.00666	0.041	t-test	0.02	0.58	Cohens_d	0.963
eyelookdownright	Activated	0.026	0.056	-0.019	0.093	t-test	0.022	-0.57	Cohens_d	1
mouthfrownleft_delta	Delta	-0.06	0.247	0.062	0.223	MWU	0.023	0.31	Rank_biserial	1
tongueout_neutral	Neutral	-5.32e-05	5.09e-05	-2.62e-05	4.41e-05	t-test	0.024	0.57	Cohens_d	1
mouthshrugupper_delta × eyelookdownleft	Interaction	-3.93e-04	0.00369	0.00195	0.00436	t-test	0.025	0.58	Cohens_d	0.721
asym_eyelookinleft	Asymmetry	0.025	0.101	-0.045	0.149	t-test	0.027	-0.55	Cohens_d	1
asym_eyewideleft_neutral	Asymmetry	1.14e-05	5.72e-05	3.17e-05	5.33e-05	MWU	0.027	0.34	Rank_biserial	1
cheekpuff_delta × eyewideright_delta	Interaction	-0.00403	0.015	0.00268	0.00862	t-test	0.03	0.56	Cohens_d	0.864
eyesquintright	Activated	0.033	0.135	-0.034	0.123	t-test	0.031	-0.52	Cohens_d	1
mouthfrownleft	Activated	-0.062	0.234	0.063	0.248	t-test	0.032	0.52	Cohens_d	1

mouthfunnel	Activated	0.014	0.121	-0.044	0.098	MWU	0.033	-0.3	Rank_biserial	1
jawright_neutral	Neutral	-0.0042	0.00713	4.45e-04	0.01	MWU	0.045	0.29	Rank_biserial	1
asym_eyewideleft_delta	Asymmetry	-0.014	0.058	0.00724	0.048	MWU	0.054	0.29	Rank_biserial	1
eyesquintright_delta	Delta	0.037	0.149	-0.019	0.077	t-test	0.059	-0.45	Cohens_d	1
asym_nosesneerleft_neutral	Asymmetry	0.00132	0.00652	-0.00165	0.00602	t-test	0.059	-0.47	Cohens_d	1
asym_eyewideleft	Asymmetry	-0.014	0.058	0.00723	0.048	MWU	0.06	0.28	Rank_biserial	1
eyesquintright_delta × eyeblinkright_neutral	Interaction	9.74e-04	0.00634	-0.00206	0.00508	MWU	0.06	-0.28	Rank_biserial	1
eyesquintright_delta × mouthshrugupper_delta	Interaction	9.77e-04	0.00635	-0.00206	0.00509	MWU	0.06	-0.28	Rank_biserial	1
eyewideleft	Activated	-0.024	0.159	0.037	0.151	MWU	0.064	0.26	Rank_biserial	1
mouthstretchleft_neutral	Neutral	-0.015	0.037	0.00211	0.036	t-test	0.067	0.45	Cohens_d	1
eyelookinright_neutral	Neutral	0.00355	0.029	-0.00871	0.022	MWU	0.069	-0.26	Rank_biserial	1
activated_range	Aggregation	0.737	0.127	0.801	0.161	t-test	0.069	0.44	Cohens_d	0.597
activated_min	Aggregation	-0.404	0.117	-0.456	0.128	MWU	0.072	-0.25	Rank_biserial	0.618
asym_mouthleft_delta	Asymmetry	0.068	0.34	-0.071	0.298	t-test	0.075	-0.43	Cohens_d	1
asym_mouthstretchleft_neutral	Asymmetry	1.65e-04	0.00551	-0.00267	0.00728	t-test	0.076	-0.44	Cohens_d	1
mouthstretchleft	Activated	-0.035	0.105	0.012	0.111	t-test	0.077	0.43	Cohens_d	1
asym_mouthleft	Asymmetry	0.068	0.341	-0.071	0.304	t-test	0.077	-0.43	Cohens_d	1
eyesquintleft	Activated	0.022	0.111	-0.023	0.1	t-test	0.082	-0.43	Cohens_d	1
delta_min	Aggregation	-0.409	0.128	-0.459	0.132	MWU	0.086	-0.24	Rank_biserial	0.736
eyeblinkright_delta	Delta	0.014	0.155	0.064	0.163	MWU	0.089	0.24	Rank_biserial	1

asym_mouthlowerdownleft_delta	Asymmetry	-0.02	0.126	0.03	0.171	MWU	0.09	0.24	Rank_biserial	1
asym_mouthstretchleft_delta	Asymmetry	-0.018	0.076	0.012	0.065	t-test	0.093	0.43	Cohens_d	1
eyelookinleft_delta	Delta	0.03	0.128	-0.03	0.169	t-test	0.098	-0.4	Cohens_d	1
eyewideright_delta × mouthfrownleft_delta	Interaction	-7.29e-04	0.038	0.012	0.02	t-test	0.099	0.41	Cohens_d	1
mouthshrugupper_delta	Delta	0.047	0.268	-0.048	0.213	t-test	0.103	-0.39	Cohens_d	1
delta_range	Aggregation	0.751	0.132	0.812	0.179	t-test	0.106	0.39	Cohens_d	0.915
asym_mouthsmileleft	Asymmetry	0.037	0.235	-0.043	0.17	t-test	0.108	-0.39	Cohens_d	1
mouthlowerdownright_neutral	Neutral	-0.011	0.021	0.00405	0.036	MWU	0.111	0.23	Rank_biserial	1
asym_eyelookinleft_neutral	Asymmetry	-0.00935	0.064	0.015	0.064	t-test	0.114	0.38	Cohens_d	1
mouthshrugupper	Activated	0.046	0.257	-0.048	0.237	t-test	0.114	-0.38	Cohens_d	1
asym_mouthsmileleft_delta	Asymmetry	0.036	0.244	-0.043	0.17	t-test	0.118	-0.38	Cohens_d	1
asym_mouthstretchleft	Asymmetry	-0.018	0.077	0.011	0.067	t-test	0.118	0.39	Cohens_d	1
cheekpuff_delta	Delta	0.094	0.109	-0.049	0.282	MWU	0.124	-0.22	Rank_biserial	1
eyelookinright_delta	Delta	-0.024	0.149	0.036	0.125	MWU	0.126	0.22	Rank_biserial	1
cheekpuff	Activated	0.092	0.108	-0.048	0.28	MWU	0.127	-0.22	Rank_biserial	1
asym_mouthdimpleleft_neutral	Asymmetry	5.63e-04	0.00101	8.50e-05	0.00138	t-test	0.129	-0.39	Cohens_d	1
eyeblinkleft	Activated	0.016	0.031	0.028	0.038	t-test	0.141	0.37	Cohens_d	1
mouthlowerdownleft_neutral	Neutral	-0.011	0.025	0.00363	0.036	MWU	0.151	0.2	Rank_biserial	1
eyelookupleft_neutral	Neutral	-0.00914	0.022	-0.016	0.013	t-test	0.151	-0.36	Cohens_d	1
eyelookupright_neutral	Neutral	-0.00917	0.022	-0.016	0.013	t-test	0.152	-0.36	Cohens_d	1

mouthfrownleft_delta × mouthshrugupper_delta	Interaction	4.88e-04	0.021	-0.00397	0.015	MWU	0.155	-0.2	Rank_biserial	1
asym_eyesquintleft_delta	Asymmetry	-0.021	0.152	0.023	0.095	t-test	0.157	0.34	Cohens_d	1
asym_eyesquintleft	Asymmetry	-0.021	0.152	0.023	0.095	t-test	0.157	0.34	Cohens_d	1
mouthstretchright	Activated	-0.02	0.127	0.021	0.126	MWU	0.162	0.19	Rank_biserial	1
asym_mouthlowerdownleft	Asymmetry	-0.02	0.126	0.031	0.171	t-test	0.163	0.34	Cohens_d	1
asym_mouthupperupleft_delta	Asymmetry	-0.017	0.147	0.03	0.136	t-test	0.166	0.33	Cohens_d	1
asym_mouthupperupleft	Asymmetry	-0.017	0.146	0.03	0.136	t-test	0.169	0.33	Cohens_d	1
jawforward	Activated	-0.018	0.147	0.018	0.121	MWU	0.177	0.19	Rank_biserial	1
eyeblinkright_neutral	Neutral	0.00277	0.087	-0.032	0.061	MWU	0.178	-0.19	Rank_biserial	1
asym_eyelookupleft	Asymmetry	0.00898	0.034	-0.00362	0.038	t-test	0.179	-0.35	Cohens_d	1
eyeblinkleft_neutral	Neutral	0.00278	0.087	-0.032	0.06	MWU	0.182	-0.19	Rank_biserial	1
eyelookinleft_neutral	Neutral	-0.0058	0.036	0.00576	0.036	t-test	0.184	0.32	Cohens_d	1
mouthrolllower	Activated	-0.022	0.168	0.023	0.121	t-test	0.195	0.31	Cohens_d	1
eyesquintleft_delta	Delta	0.00802	0.113	-0.026	0.104	t-test	0.201	-0.31	Cohens_d	1
neutral_max	Aggregation	0.172	0.096	0.146	0.066	t-test	0.202	-0.31	Cohens_d	1
asym_eyelookdownleft_neutral	Asymmetry	4.31e-05	1.87e-04	-5.27e-06	1.58e-04	MWU	0.203	-0.18	Rank_biserial	1
asym_eyelookoutleft_neutral	Asymmetry	0.00648	0.0035	0.00595	0.00198	MWU	0.207	-0.2	Rank_biserial	1
browdownleft	Activated	0.028	0.164	-0.029	0.183	MWU	0.208	-0.17	Rank_biserial	1
eyewideleft_neutral	Neutral	-0.028	0.031	-0.00272	0.054	MWU	0.21	0.18	Rank_biserial	1
eyewideright_neutral	Neutral	-0.028	0.031	-0.00276	0.054	MWU	0.21	0.18	Rank_biserial	1

eyewideleft_delta	Delta	-0.023	0.181	0.023	0.171	MWU	0.212	0.17	Rank_biserial	1
mouthright_neutral	Neutral	-0.00408	0.00725	-0.00175	0.00867	MWU	0.212	0.18	Rank_biserial	1
browdownleft_neutral	Neutral	0.016	0.134	-0.029	0.139	MWU	0.213	-0.17	Rank_biserial	1
browdownright_neutral	Neutral	0.016	0.134	-0.029	0.139	MWU	0.217	-0.17	Rank_biserial	1
eyeblinkright	Activated	0.059	0.07	0.062	0.091	MWU	0.228	0.18	Rank_biserial	1
mouthright	Activated	-0.042	0.312	0.043	0.291	MWU	0.229	0.17	Rank_biserial	1
cheekpuff_delta × mouthfrownleft_delta	Interaction	-0.00241	0.023	0.00354	0.016	t-test	0.236	0.3	Cohens_d	1
mouthlowerdownright_delta × eyeblinkright_neutral	Interaction	0.00118	0.00392	0.00283	0.012	MWU	0.236	0.19	Rank_biserial	1
mouthlowerdownright_delta × mouthshrugupper_delta	Interaction	0.00119	0.00392	0.00284	0.012	MWU	0.236	0.19	Rank_biserial	1
mouthrolllower_delta	Delta	-0.016	0.17	0.028	0.135	t-test	0.237	0.28	Cohens_d	1
asym_eyelookoutleft_delta	Asymmetry	-0.021	0.111	0.012	0.127	t-test	0.243	0.28	Cohens_d	1
mouthsmileleft_delta	Delta	0.024	0.172	-0.025	0.186	t-test	0.256	-0.27	Cohens_d	1
asym_browdownleft_neutral	Asymmetry	-1.05e-04	1.08e-04	-7.64e-05	1.48e-04	MWU	0.259	0.17	Rank_biserial	1
eyelookinright	Activated	-0.02	0.144	0.021	0.163	t-test	0.261	0.27	Cohens_d	1
mouthleft_neutral	Neutral	-0.00215	0.00755	-0.00347	0.00741	MWU	0.261	-0.16	Rank_biserial	1
neutral_std	Aggregation	0.055	0.02	0.051	0.014	t-test	0.261	-0.27	Cohens_d	1
eyelookoutright_neutral	Neutral	-0.00655	0.00352	-0.00609	0.00205	MWU	0.267	0.17	Rank_biserial	1
asym_eyelookoutleft	Asymmetry	-0.02	0.109	0.011	0.125	t-test	0.274	0.26	Cohens_d	1
asym_eyesquintleft_neutral	Asymmetry	5.18e-06	3.51e-05	1.47e-05	2.09e-05	MWU	0.274	0.17	Rank_biserial	1
mouthupperupright_delta	Delta	0.025	0.154	-0.025	0.152	MWU	0.277	-0.15	Rank_biserial	1

cheeksquintright	Activated	-0.00548	0.138	0.029	0.119	t-test	0.281	0.27	Cohens_d	1
mouthright_delta	Delta	-0.041	0.311	0.042	0.289	MWU	0.282	0.15	Rank_biserial	1
neutral_range	Aggregation	0.276	0.101	0.256	0.079	MWU	0.286	-0.15	Rank_biserial	1
mouthsmileleft	Activated	0.022	0.17	-0.023	0.183	t-test	0.29	-0.25	Cohens_d	1
mouthstretchleft_delta	Delta	-0.025	0.105	0.00111	0.103	t-test	0.295	0.25	Cohens_d	1
mouthupperupright	Activated	0.025	0.148	-0.016	0.135	MWU	0.304	-0.14	Rank_biserial	1
cheekpuff_delta × mouthlowerdownright_delta	Interaction	-0.00184	0.00801	-0.00257	0.014	MWU	0.311	-0.16	Rank_biserial	1
mouthfrownleft_delta × mouthlowerdownright_delta	Interaction	0.003	0.012	-0.00312	0.026	MWU	0.318	-0.15	Rank_biserial	1
asym_browdownleft	Asymmetry	-0.014	0.126	-0.048	0.149	t-test	0.324	-0.25	Cohens_d	1
asym_browdownleft_delta	Asymmetry	-0.014	0.126	-0.048	0.149	t-test	0.324	-0.25	Cohens_d	1
jawforward_delta	Delta	-0.012	0.15	0.00239	0.106	MWU	0.326	0.14	Rank_biserial	1
asym_mouthfrownleft_neutral	Asymmetry	-0.00466	0.00532	-0.00129	0.01	MWU	0.335	0.14	Rank_biserial	1
asym_eyeblinkleft_delta	Asymmetry	-0.045	0.086	-0.036	0.085	MWU	0.336	0.15	Rank_biserial	1
asym_eyeblinkleft	Asymmetry	-0.045	0.086	-0.036	0.085	MWU	0.336	0.15	Rank_biserial	1
eyelookoutleft_delta	Delta	-0.011	0.121	0.018	0.105	MWU	0.338	0.13	Rank_biserial	1
eyelookoutleft	Activated	-0.011	0.121	0.018	0.105	MWU	0.338	0.13	Rank_biserial	1
asym_eyelookupleft_delta	Asymmetry	0.00895	0.034	-3.16e-04	0.041	t-test	0.342	-0.24	Cohens_d	1
mouthpucker	Activated	0.038	0.059	0.026	0.049	t-test	0.36	-0.23	Cohens_d	1
jawforward_neutral	Neutral	-0.00568	0.02	2.31e-04	0.022	MWU	0.364	0.13	Rank_biserial	1
browdownright_delta	Delta	-0.022	0.179	0.022	0.224	t-test	0.366	0.22	Cohens_d	1

mouthpressright_delta	Delta	0.014	0.161	-0.014	0.189	MWU	0.373	-0.12	Rank_biserial	1
eyelookinleft	Activated	0.024	0.136	-6.89e-04	0.137	MWU	0.377	-0.12	Rank_biserial	1
mouthfrownright_delta	Delta	-0.028	0.196	0.013	0.194	t-test	0.379	0.21	Cohens_d	1
mouthdimpleright_neutral	Neutral	-0.0045	0.017	-0.00117	0.015	t-test	0.384	0.21	Cohens_d	1
mouthleft_delta	Delta	0.046	0.234	-0.028	0.294	MWU	0.385	-0.12	Rank_biserial	1
mouthpressright_neutral	Neutral	-0.00209	0.047	-0.00975	0.022	t-test	0.389	-0.21	Cohens_d	1
mouthpressright	Activated	0.018	0.169	-0.018	0.183	t-test	0.393	-0.2	Cohens_d	1
asym_mouthpressleft_neutral	Asymmetry	0.00128	0.00232	7.59e-04	0.00251	t-test	0.394	-0.21	Cohens_d	1
browouterupright	Activated	0.00479	0.162	0.052	0.167	MWU	0.407	0.12	Rank_biserial	1
mouthdimpleleft	Activated	-0.00746	0.122	-0.00111	0.094	MWU	0.407	0.12	Rank_biserial	1
asym_mouthlowerdownleft_neutral	Asymmetry	4.22e-04	0.00411	-4.36e-04	0.00419	t-test	0.408	-0.21	Cohens_d	1
asym_jawleft_neutral	Asymmetry	-4.69e-04	0.015	-0.00342	0.015	t-test	0.42	-0.2	Cohens_d	1
mouthlowerdownleft_delta	Delta	0.0088	0.149	-0.02	0.156	t-test	0.426	-0.19	Cohens_d	1
mouthdimpleleft_neutral	Neutral	-0.00418	0.016	-0.00129	0.014	t-test	0.428	0.19	Cohens_d	1
mouthleft	Activated	0.046	0.232	-0.027	0.298	MWU	0.438	-0.11	Rank_biserial	1
mouthupperupleft	Activated	0.0087	0.166	-0.019	0.139	t-test	0.447	-0.18	Cohens_d	1
mouthfrownleft_delta × eyeblinkright_neutral	Interaction	4.77e-04	0.021	-0.00279	0.013	t-test	0.449	-0.19	Cohens_d	1
asym_eyelookupleft_neutral	Asymmetry	3.13e-05	8.49e-05	1.77e-05	5.59e-05	t-test	0.455	-0.19	Cohens_d	1
browouterupleft	Activated	-0.00361	0.155	0.022	0.186	MWU	0.463	0.1	Rank_biserial	1
mouthdimpleleft_delta	Delta	-0.00703	0.124	-0.00206	0.095	MWU	0.47	0.1	Rank_biserial	1

mouthsmileright	Activated	-0.015	0.178	0.015	0.173	t-test	0.475	0.17	Cohens_d	1
mouthshruglower	Activated	-0.019	0.214	0.019	0.203	MWU	0.479	0.1	Rank_biserial	1
asym_mouthupperupleft_neutral	Asymmetry	5.67e-04	0.00191	2.28e-04	0.00191	t-test	0.481	-0.18	Cohens_d	1
mouthupperupleft_delta	Delta	0.00768	0.167	-0.018	0.139	t-test	0.482	-0.17	Cohens_d	1
mouthfrownleft_delta × eyeblinkleft_neutral	Interaction	-5.10e-04	0.00463	1.88e-04	0.00342	t-test	0.484	0.17	Cohens_d	1
eyesquintleft_neutral	Neutral	-0.00848	0.04	-0.00165	0.042	t-test	0.49	0.17	Cohens_d	1
eyesquintright_neutral	Neutral	-0.00847	0.04	-0.00165	0.042	t-test	0.49	0.17	Cohens_d	1
asym_browouterupleft	Asymmetry	-0.00978	0.081	-0.023	0.081	t-test	0.511	-0.17	Cohens_d	1
mouthfrownright	Activated	-0.016	0.212	0.017	0.21	t-test	0.518	0.15	Cohens_d	1
mouthshruglower_delta	Delta	-0.016	0.215	0.016	0.204	t-test	0.518	0.15	Cohens_d	1
mouthstretchright_delta	Delta	-0.00904	0.118	0.0093	0.12	t-test	0.519	0.15	Cohens_d	1
neutral_min	Aggregation	-0.097	0.035	-0.101	0.034	MWU	0.527	-0.09	Rank_biserial	1
asym_browouterupleft_delta	Asymmetry	-0.01	0.081	-0.023	0.081	t-test	0.538	-0.16	Cohens_d	1
eyewideright_delta × eyeblinkleft_neutral	Interaction	7.07e-04	0.00328	2.62e-04	0.00268	t-test	0.544	-0.15	Cohens_d	1
asym_eyeblinkleft_neutral	Asymmetry	2.92e-05	5.88e-05	3.67e-05	2.77e-05	t-test	0.55	0.16	Cohens_d	1
jawleft_delta	Delta	0.017	0.173	-0.017	0.167	MWU	0.561	-0.08	Rank_biserial	1
mouthrollupper_neutral	Neutral	-0.00573	0.018	-0.00323	0.017	t-test	0.567	0.14	Cohens_d	1
activated_std	Aggregation	0.16	0.022	0.163	0.025	t-test	0.573	0.13	Cohens_d	1
mouthshrugupper_delta × eyeblinkleft_neutral	Interaction	2.61e-04	0.00135	5.18e-05	0.00169	t-test	0.576	-0.14	Cohens_d	1
mouthlowerdownright_delta × cheekpuff	Interaction	-3.00e-04	0.00204	-7.91e-04	0.00448	t-test	0.583	-0.14	Cohens_d	1

asym_eyelookdownleft_delta	Asymmetry	-0.0078	0.039	-0.00215	0.043	t-test	0.584	0.14	Cohens_d	1
eyelookdownright_neutral	Neutral	-0.00369	0.071	-0.00931	0.093	MWU	0.585	-0.08	Rank_biserial	1
mouthdimpleright_delta	Delta	-0.015	0.122	-0.00539	0.106	MWU	0.585	0.08	Rank_biserial	1
eyelookdownleft_neutral	Neutral	-0.00363	0.071	-0.00933	0.093	MWU	0.585	-0.08	Rank_biserial	1
asym_eyelookdownleft	Asymmetry	-0.00776	0.039	-0.00218	0.043	t-test	0.588	0.14	Cohens_d	1
mouthsmileright_delta	Delta	-0.012	0.205	0.013	0.183	t-test	0.59	0.13	Cohens_d	1
cheekpuff_delta × eyesquintright_delta	Interaction	0.00189	0.00909	8.34e-04	0.00482	t-test	0.591	-0.14	Cohens_d	1
asym_mouthpressleft	Asymmetry	4.74e-04	0.1	0.014	0.102	t-test	0.596	0.13	Cohens_d	1
mouthrolllower_neutral	Neutral	-0.016	0.051	-0.00972	0.053	t-test	0.597	0.13	Cohens_d	1
cheeksquintleft_delta	Delta	0.011	0.186	-0.012	0.191	t-test	0.611	-0.12	Cohens_d	1
jawleft	Activated	0.017	0.176	-0.017	0.17	MWU	0.617	-0.07	Rank_biserial	1
jawopen_delta	Delta	0.031	0.072	0.022	0.078	t-test	0.618	-0.12	Cohens_d	1
asym_mouthsmileleft_neutral	Asymmetry	-2.76e-05	0.01	-9.80e-04	0.011	MWU	0.62	-0.08	Rank_biserial	1
browouterupright_delta	Delta	0.02	0.164	0.052	0.165	MWU	0.62	0.07	Rank_biserial	1
asym_mouthpressleft_delta	Asymmetry	0.00107	0.101	0.013	0.102	t-test	0.627	0.12	Cohens_d	1
mouthshrugupper_delta × cheekpuff	Interaction	2.69e-05	0.0029	-2.66e-04	0.00215	t-test	0.641	-0.11	Cohens_d	1
browouterupleft_delta	Delta	0.013	0.155	0.022	0.183	MWU	0.644	0.07	Rank_biserial	1
mouthpucker_delta	Delta	0.025	0.105	0.029	0.071	MWU	0.644	-0.07	Rank_biserial	1
mouthpressleft_delta	Delta	-0.0097	0.165	0.00998	0.192	t-test	0.645	0.11	Cohens_d	1
mouthpucker_neutral	Neutral	-0.00834	0.046	-0.018	0.033	MWU	0.66	-0.06	Rank_biserial	1

mouthsmileright_neutral	Neutral	-0.019	0.036	-0.0024	0.061	MWU	0.665	0.06	Rank_biserial	1
asym_jawleft	Asymmetry	0.016	0.181	4.12e-04	0.12	t-test	0.666	-0.1	Cohens_d	1
asym_cheeksquintleft_neutral	Asymmetry	0.00174	0.00709	0.00105	0.00655	t-test	0.681	-0.1	Cohens_d	1
browinnerup	Activated	-0.00445	0.19	0.00458	0.226	MWU	0.683	0.06	Rank_biserial	1
asym_cheeksquintleft	Asymmetry	-0.00417	0.101	0.00641	0.112	t-test	0.685	0.1	Cohens_d	1
asym_cheeksquintleft_delta	Asymmetry	-0.00412	0.102	0.00659	0.113	t-test	0.686	0.1	Cohens_d	1
cheeksquintleft	Activated	0.00848	0.17	-0.00872	0.194	t-test	0.693	-0.09	Cohens_d	1
mouthfrownleft_delta × cheekpuff	Interaction	-0.00137	0.011	-6.22e-04	0.00481	t-test	0.704	0.09	Cohens_d	1
activated_mean	Aggregation	0.00218	0.046	-0.00224	0.053	t-test	0.709	-0.09	Cohens_d	1
jawright_delta	Delta	0.00168	0.161	-0.00173	0.154	MWU	0.717	-0.05	Rank_biserial	1
neutral_mean	Aggregation	8.47e-04	0.023	-8.71e-04	0.017	t-test	0.718	-0.09	Cohens_d	1
mouthshrugupper_delta × eyeblinkright_neutral	Interaction	0.00568	0.00575	0.00494	0.00405	MWU	0.724	-0.05	Rank_biserial	1
mouthupperupleft_neutral	Neutral	-0.00418	0.011	-0.00253	0.013	MWU	0.724	0.05	Rank_biserial	1
cheeksquintright_delta	Delta	0.00682	0.167	-0.00702	0.167	t-test	0.728	-0.08	Cohens_d	1
mouthsmileleft_neutral	Neutral	-0.017	0.035	-0.00431	0.056	MWU	0.73	0.05	Rank_biserial	1
nosesneerleft_delta	Delta	-0.00699	0.238	0.025	0.204	MWU	0.738	0.05	Rank_biserial	1
mouthshrugupper_neutral	Neutral	-0.00886	0.084	-0.02	0.057	MWU	0.74	-0.05	Rank_biserial	1
tongueout_delta	Delta	8.60e-04	0.00107	0.00113	8.11e-04	MWU	0.741	0.05	Rank_biserial	1
eyelookupleft_delta	Delta	1.49e-04	0.175	0.011	0.136	t-test	0.777	0.07	Cohens_d	1
mouthpressleft_neutral	Neutral	-0.00227	0.046	-0.012	0.021	MWU	0.778	-0.04	Rank_biserial	1

mouthclose	Activated	0.00413	0.136	-0.00425	0.118	t-test	0.782	-0.07	Cohens_d	1
cheekpuff_neutral	Neutral	-0.00117	0.016	-0.00167	0.011	MWU	0.782	0.04	Rank_biserial	1
cheekpuff_delta × mouthshrugupper_delta	Interaction	-0.00506	0.00485	-0.00499	0.00466	MWU	0.8	-0.04	Rank_biserial	1
browdownright	Activated	0.00633	0.159	-0.00651	0.145	MWU	0.805	-0.03	Rank_biserial	1
cheeksquintleft_neutral	Neutral	-0.00277	0.048	-0.00513	0.036	MWU	0.805	0.04	Rank_biserial	1
eyelookupleft	Activated	0.00309	0.168	-0.0017	0.123	MWU	0.805	-0.04	Rank_biserial	1
eyelookupright_delta	Delta	0.0043	0.16	-0.00442	0.144	t-test	0.81	-0.06	Cohens_d	1
mouthlowerdownleft	Activated	-0.0071	0.135	-0.016	0.163	t-test	0.813	-0.06	Cohens_d	1
nosesneerright_delta	Delta	-0.00618	0.237	0.00636	0.217	t-test	0.817	0.06	Cohens_d	1
mouthclose_delta	Delta	0.00859	0.14	0.00269	0.102	t-test	0.841	-0.05	Cohens_d	1
mouthupperupright_neutral	Neutral	-0.00369	0.012	-0.00214	0.015	MWU	0.846	0.03	Rank_biserial	1
delta_mean	Aggregation	0.00128	0.056	-0.00131	0.056	t-test	0.846	-0.05	Cohens_d	1
mouthlowerdownright_delta × eyeblinkleft_neutral	Interaction	1.00e-04	0.00105	-1.93e-05	0.00334	t-test	0.849	-0.05	Cohens_d	1
tongueout	Activated	8.72e-04	0.001	0.00112	8.26e-04	MWU	0.862	0.03	Rank_biserial	1
mouthfrownleft_neutral	Neutral	-0.017	0.057	-0.021	0.045	MWU	0.873	0.02	Rank_biserial	1
mouthrollupper	Activated	-0.00364	0.208	0.00374	0.167	MWU	0.877	0.02	Rank_biserial	1
asym_nosesneerleft_delta	Asymmetry	-0.033	0.114	-0.03	0.073	t-test	0.881	0.04	Cohens_d	1
mouthpressleft	Activated	-0.0064	0.188	0.00658	0.184	MWU	0.886	0.02	Rank_biserial	1
mouthclose_neutral	Neutral	-0.00446	0.03	-0.00183	0.033	MWU	0.89	0.02	Rank_biserial	1
jawopen_neutral	Neutral	-0.00448	0.026	-0.00416	0.027	MWU	0.897	-0.02	Rank_biserial	1

nosesneerleft_neutral	Neutral	-0.019	0.029	-0.02	0.036	t-test	0.899	-0.03	Cohens_d	1
asym_jawleft_delta	Asymmetry	0.015	0.172	0.011	0.102	t-test	0.906	-0.03	Cohens_d	1
nosesneerleft	Activated	-0.00128	0.221	0.02	0.182	MWU	0.911	0.02	Rank_biserial	1
mouthfunnel_neutral	Neutral	-0.00518	0.036	-0.00426	0.032	t-test	0.912	0.03	Cohens_d	1
jawright	Activated	1.55e-04	0.164	-1.60e-04	0.154	MWU	0.913	-0.02	Rank_biserial	1
asym_browouterupleft_neutral	Asymmetry	6.73e-05	1.31e-04	4.59e-05	1.20e-04	MWU	0.927	0.01	Rank_biserial	1
eyewideright_delta × mouthshrugupper_delta	Interaction	9.13e-04	0.012	0.0011	0.00768	t-test	0.94	0.02	Cohens_d	1
asym_mouthdimpleleft_delta	Asymmetry	-0.0075	0.028	-0.00665	0.06	t-test	0.941	0.02	Cohens_d	1
eyewideright_delta × eyeblinkright_neutral	Interaction	9.11e-04	0.012	0.00109	0.00765	t-test	0.942	0.02	Cohens_d	1
eyelookoutright_delta	Delta	0.00126	0.14	-0.00129	0.163	t-test	0.944	-0.02	Cohens_d	1
nosesneerright_neutral	Neutral	-0.02	0.029	-0.02	0.033	t-test	0.959	-0.01	Cohens_d	1
jawopen	Activated	0.029	0.066	0.024	0.078	MWU	0.959	0.01	Rank_biserial	1
eyesquintright_delta × mouthfrownleft_delta	Interaction	-2.67e-04	0.027	-5.06e-04	0.014	t-test	0.964	-0.01	Cohens_d	1
eyelookupright	Activated	0.00721	0.154	0.022	0.109	MWU	0.966	-0.01	Rank_biserial	1
mouthshruglower_neutral	Neutral	-0.00371	0.109	-0.011	0.08	MWU	0.967	0.01	Rank_biserial	1
jawleft_neutral	Neutral	-0.00351	0.0039	-0.00477	0.00251	MWU	0.971	-0.01	Rank_biserial	1
asym_mouthdimpleleft	Asymmetry	-0.00732	0.028	-0.00695	0.06	t-test	0.974	0.01	Cohens_d	1
mouthfrownright_neutral	Neutral	-0.022	0.041	-0.025	0.034	MWU	0.979	0	Rank_biserial	1
activated_max	Aggregation	0.333	0.08	0.333	0.055	t-test	0.98	-0.01	Cohens_d	1
delta_max	Aggregation	0.342	0.086	0.342	0.073	t-test	0.983	-0.01	Cohens_d	1

cheeksquintright_neutral	Neutral	-0.00293	0.052	-0.00847	0.037	MWU	0.985	-0	Rank_biserial	1
mouthrollupper_delta	Delta	-2.42e-04	0.199	2.48e-04	0.159	MWU	0.986	0	Rank_biserial	1
asym_nosesneerleft	Asymmetry	-0.032	0.117	-0.031	0.073	t-test	0.987	0	Cohens_d	1
eyesquintright_delta × mouthlowerdownright_delta	Interaction	-2.93e-04	0.00622	-3.21e-04	0.0093	t-test	0.99	-0	Cohens_d	1
eyelookoutright	Activated	-2.17e-04	0.14	2.23e-04	0.163	t-test	0.99	0	Cohens_d	1
browinnerup_delta	Delta	0.024	0.184	-0.00106	0.248	MWU	0.991	0	Rank_biserial	1
asym_mouthleft_neutral	Asymmetry	0.00289	0.015	0.00294	0.02	t-test	0.991	0	Cohens_d	1
delta_std	Aggregation	0.166	0.024	0.166	0.028	t-test	0.991	0	Cohens_d	1
browdownleft_delta	Delta	1.92e-04	0.196	-1.97e-04	0.215	t-test	0.994	-0	Cohens_d	1
mouthdimpleright	Activated	-0.00754	0.128	-0.02	0.084	MWU	0.995	-0	Rank_biserial	1
nosesneerright	Activated	-0.00245	0.215	0.00252	0.205	MWU	0.995	0	Rank_biserial	1
mouthlowerdownright_delta × eyelookdownright	Interaction	9.17e-04	0.00623	8.93e-04	0.00841	MWU	1	0	Rank_biserial	1

Supplementary Table 4. Complete results of all between-group comparisons between people with Parkinson's disease (PD; n = 34) and healthy controls (HC; n = 36) across the 261 engineered facial blendshape features. Outliers were removed separately within each diagnostic group using the Tukey interquartile-range rule. Normality was assessed with the Shapiro-Wilk test. Between-group tests used the independent-samples t-test with Welch correction for normally distributed features and the two-tailed MWU test otherwise. Effect sizes are Cohen's d (t-test) or rank-biserial correlation (MWU). Multiple testing was controlled within each of the six feature families using *Meff*-based Bonferroni correction. MWU: Mann-Whitney U test

Supplementary Table 5 | Clinical correlations with MDS-UPDRS III sum score

Feature	Feature group	r	p value	Method	p_adj
eyeblinkleft_delta	Delta	0.61	8.28e-04	Spearman	0.031
eyewideright	Activated	-0.59	0.00114	Pearson	0.044
eyewideright_delta	Delta	-0.57	0.00198	Pearson	0.074
asym_eyesquintleft_delta	Asymmetry	-0.5	0.00819	Pearson	0.344
asym_eyesquintleft	Asymmetry	-0.5	0.0082	Pearson	0.344
mouthrollupper_neutral	Neutral	-0.49	0.01	Pearson	0.365
eyelookinright_delta	Delta	-0.45	0.019	Pearson	0.722
browdownright	Activated	-0.43	0.02	Pearson	0.782
mouthrolllower_neutral	Neutral	-0.43	0.023	Pearson	0.821
mouthfrownright_neutral	Neutral	0.43	0.027	Pearson	0.979
mouthfrownleft_neutral	Neutral	0.41	0.033	Pearson	1
eyeblinkleft_neutral	Neutral	-0.4	0.034	Spearman	1
eyeblinkright_neutral	Neutral	-0.4	0.034	Spearman	1
asym_mouthlowerdownleft_neutral	Asymmetry	-0.4	0.039	Pearson	1
mouthleft	Activated	-0.38	0.042	Spearman	1
eyewideleft	Activated	-0.38	0.056	Pearson	1
mouthleft_delta	Delta	-0.38	0.045	Spearman	1
delta_max	Aggregation	-0.37	0.056	Pearson	0.449
asym_eyeblinkleft_neutral	Asymmetry	-0.37	0.083	Pearson	1
mouthsmileright	Activated	-0.37	0.051	Pearson	1
asym_mouthfrownleft_neutral	Asymmetry	0.36	0.065	Spearman	1
mouthpucker × mouthleft	Interaction	-0.35	0.064	Spearman	1
eyelookdownright	Activated	-0.34	0.073	Pearson	1
eyelookupleft_neutral	Neutral	-0.34	0.114	Pearson	1
asym_eyewideleft	Asymmetry	0.34	0.115	Pearson	1
asym_eyewideleft_delta	Asymmetry	0.34	0.116	Pearson	1

eyelookupright_neutral	Neutral	-0.34	0.118	Pearson	1
mouthdimpleright_neutral	Neutral	-0.33	0.082	Pearson	1
jawleft	Activated	-0.33	0.079	Pearson	1
mouthdimpleleft_neutral	Neutral	-0.33	0.086	Pearson	1
mouthleft × mouthsmileright	Interaction	-0.33	0.082	Pearson	1
mouthrollupper × mouthstretchright	Interaction	-0.33	0.095	Pearson	1
browdownright_delta	Delta	-0.33	0.085	Pearson	1
asym_browouterupleft_neutral	Asymmetry	0.32	0.1	Spearman	1
eyewideleft_neutral	Neutral	0.32	0.091	Spearman	1
eyewideright_neutral	Neutral	0.32	0.091	Spearman	1
asym_mouthpressleft_neutral	Asymmetry	0.32	0.092	Pearson	1
asym_cheeksquintleft_neutral	Asymmetry	-0.32	0.106	Pearson	1
jawleft_delta	Delta	-0.31	0.097	Pearson	1
mouthlowerdownleft	Activated	-0.31	0.104	Pearson	1
eyeblinkright_delta	Delta	0.31	0.123	Pearson	1
asym_jawleft	Asymmetry	-0.31	0.108	Pearson	1
jawforward_neutral	Neutral	-0.3	0.127	Pearson	1
eyelookinright	Activated	-0.3	0.141	Pearson	1
mouthrolllower_delta	Delta	0.29	0.121	Pearson	1
delta_mean	Aggregation	-0.29	0.126	Pearson	1
tongueout_delta	Delta	-0.29	0.161	Pearson	1
mouthstretchright_delta	Delta	-0.29	0.14	Pearson	1
tongueout	Activated	-0.29	0.167	Pearson	1
eyelookoutleft_delta	Delta	-0.28	0.151	Pearson	1
mouthleft × mouthshrugupper	Interaction	-0.28	0.136	Pearson	1
eyelookoutleft	Activated	-0.28	0.154	Pearson	1
mouthsmileright_delta	Delta	-0.28	0.14	Pearson	1
mouthstretchleft_delta	Delta	-0.28	0.14	Pearson	1

mouthpressleft_neutral	Neutral	-0.28	0.157	Pearson	1
asym_browdownleft_neutral	Asymmetry	0.28	0.199	Pearson	1
mouthlowerdownleft_delta	Delta	-0.27	0.157	Pearson	1
asym_jawleft_delta	Asymmetry	-0.27	0.175	Pearson	1
mouthlowerdownright	Activated	-0.27	0.177	Pearson	1
mouthlowerdownleft_neutral	Neutral	-0.27	0.161	Pearson	1
asym_eyelookupleft_neutral	Asymmetry	-0.26	0.196	Pearson	1
asym_browdownleft_delta	Asymmetry	0.26	0.173	Pearson	1
asym_browdownleft	Asymmetry	0.26	0.175	Pearson	1
mouthsmileleft	Activated	-0.25	0.189	Pearson	1
mouthupperupleft	Activated	-0.24	0.219	Pearson	1
activated_mean	Aggregation	-0.24	0.211	Pearson	1
mouthstretchleft	Activated	-0.24	0.205	Pearson	1
asym_mouthleft	Asymmetry	-0.23	0.242	Pearson	1
browouterupright	Activated	-0.23	0.226	Pearson	1
mouthupperupleft_delta	Delta	-0.23	0.248	Pearson	1
cheeksquintright	Activated	-0.23	0.288	Spearman	1
mouthlowerdownright_neutral	Neutral	-0.22	0.259	Pearson	1
eyelookdownleft	Activated	-0.22	0.244	Pearson	1
mouthleft × mouthrolllower	Interaction	-0.22	0.246	Pearson	1
eyelookoutright_delta	Delta	-0.22	0.247	Pearson	1
jawopen	Activated	-0.22	0.3	Spearman	1
browouterupright_delta	Delta	-0.22	0.25	Pearson	1
asym_mouthleft_delta	Asymmetry	-0.22	0.277	Pearson	1
mouthshrugupper × mouthsmileright	Interaction	-0.22	0.258	Pearson	1
mouthstretchright	Activated	-0.22	0.26	Pearson	1
asym_browouterupleft_delta	Asymmetry	0.22	0.29	Pearson	1
asym_browouterupleft	Asymmetry	0.21	0.292	Pearson	1

eyesquintright_delta	Delta	0.21	0.272	Pearson	1
mouthpucker	Activated	0.21	0.303	Pearson	1
eyelookdownleft_neutral	Neutral	-0.21	0.279	Pearson	1
eyelookdownright_neutral	Neutral	-0.21	0.279	Pearson	1
browinnerup_neutral	Neutral	0.2	0.322	Pearson	1
eyelookoutright	Activated	-0.2	0.293	Pearson	1
eyesquintright	Activated	0.2	0.296	Pearson	1
eyelookupright	Activated	-0.2	0.317	Pearson	1
mouthleft × mouthshruglower	Interaction	-0.2	0.299	Spearman	1
cheeksquintleft_delta	Delta	-0.2	0.302	Pearson	1
mouthfrownright_delta	Delta	0.2	0.307	Pearson	1
mouthshruglower_delta	Delta	0.2	0.31	Pearson	1
mouthfrownright	Activated	0.19	0.319	Pearson	1
cheeksquintleft	Activated	-0.19	0.319	Pearson	1
asym_mouthdimpleleft_neutral	Asymmetry	0.19	0.339	Pearson	1
asym_eyelookoutleft_delta	Asymmetry	0.19	0.324	Pearson	1
asym_eyesquintleft_neutral	Asymmetry	-0.18	0.356	Spearman	1
jawright_delta	Delta	-0.18	0.348	Pearson	1
mouthshrugupper × mouthsmileleft	Interaction	-0.18	0.35	Pearson	1
browouterupleft	Activated	-0.18	0.352	Pearson	1
asym_eyeblinkleft_delta	Asymmetry	0.18	0.394	Pearson	1
asym_eyeblinkleft	Asymmetry	0.18	0.394	Pearson	1
mouthlowerdownright_delta	Delta	-0.18	0.408	Pearson	1
nosesneerleft_delta	Delta	-0.18	0.361	Spearman	1
jawright	Activated	-0.17	0.369	Pearson	1
mouthpressright_neutral	Neutral	-0.17	0.426	Pearson	1
mouthshruglower	Activated	0.17	0.378	Pearson	1
browouterupright_neutral	Neutral	-0.17	0.423	Pearson	1
eyelookoutright_neutral	Neutral	0.17	0.417	Pearson	1

mouthpucker_neutral	Neutral	-0.17	0.417	Pearson	1
asym_mouthstretchleft_delta	Asymmetry	0.16	0.403	Pearson	1
browouterupleft_neutral	Neutral	-0.16	0.433	Pearson	1
cheeksquintright_delta	Delta	-0.16	0.397	Pearson	1
browouterupleft_delta	Delta	-0.16	0.404	Pearson	1
asym_eyelookoutleft	Asymmetry	0.16	0.408	Pearson	1
mouthshruglower × mouthsmileleft	Interaction	-0.16	0.42	Spearman	1
mouthshruglower × mouthstretchright	Interaction	-0.16	0.416	Pearson	1
mouthsmileleft_delta	Delta	-0.16	0.417	Pearson	1
asym_mouthstretchleft_neutral	Asymmetry	-0.15	0.44	Pearson	1
neutral_mean	Aggregation	-0.15	0.431	Pearson	1
mouthpucker × mouthshruglower	Interaction	0.15	0.425	Pearson	1
mouthshrugupper	Activated	-0.15	0.43	Pearson	1
browinnerup	Activated	-0.15	0.432	Pearson	1
asym_mouthupperupleft_neutral	Asymmetry	-0.15	0.476	Pearson	1
mouthrolllower × mouthsmileleft	Interaction	-0.15	0.455	Pearson	1
mouthshrugupper_delta	Delta	-0.15	0.447	Pearson	1
eyelookinleft_delta	Delta	-0.15	0.447	Pearson	1
mouthrolllower	Activated	0.14	0.467	Pearson	1
mouthshruglower × mouthstretchleft	Interaction	-0.14	0.466	Pearson	1
mouthsmileright_neutral	Neutral	-0.14	0.487	Spearman	1
mouthrolllower × mouthshruglower	Interaction	0.14	0.479	Pearson	1
asym_eyelookdownleft	Asymmetry	0.13	0.496	Pearson	1
asym_eyelookdownleft_delta	Asymmetry	0.13	0.497	Pearson	1
neutral_max	Aggregation	-0.13	0.488	Pearson	1
neutral_range	Aggregation	-0.13	0.488	Pearson	1
asym_eyelookupleft	Asymmetry	-0.13	0.506	Pearson	1
asym_eyelookoutleft_neutral	Asymmetry	-0.13	0.515	Pearson	1

asym_eyelookupleft_delta	Asymmetry	-0.13	0.51	Pearson	1
asym_nosesneerleft_delta	Asymmetry	0.13	0.528	Pearson	1
mouthdimpleleft	Activated	-0.13	0.5	Pearson	1
mouthleft × mouthrollupper	Interaction	-0.13	0.5	Spearman	1
eyelookdownright_delta	Delta	0.13	0.503	Pearson	1
mouthrollupper × mouthsmileright	Interaction	-0.13	0.506	Pearson	1
mouthdimpleright	Activated	-0.13	0.507	Pearson	1
mouthshruglower_neutral	Neutral	0.13	0.519	Pearson	1
eyelookinleft	Activated	-0.13	0.511	Pearson	1
jawopen_delta	Delta	-0.12	0.544	Pearson	1
mouthright_neutral	Neutral	-0.12	0.538	Spearman	1
asym_nosesneerleft	Asymmetry	0.12	0.556	Pearson	1
neutral_std	Aggregation	-0.12	0.537	Pearson	1
mouthpucker × mouthshrugupper	Interaction	-0.12	0.538	Pearson	1
mouthfrownleft_delta	Delta	0.12	0.542	Pearson	1
asym_mouthstretchleft	Asymmetry	0.12	0.554	Pearson	1
mouthsmileleft_neutral	Neutral	-0.12	0.558	Spearman	1
mouthstretchright_neutral	Neutral	0.11	0.554	Pearson	1
browinnerup_delta	Delta	-0.11	0.564	Pearson	1
asym_mouthsmileleft_delta	Asymmetry	0.11	0.578	Pearson	1
mouthfrownleft	Activated	0.11	0.578	Pearson	1
mouthrollupper × mouthshruglower	Interaction	0.11	0.591	Spearman	1
eyesquintleft_delta	Delta	-0.11	0.586	Pearson	1
mouthshrugupper_neutral	Neutral	0.11	0.595	Spearman	1
mouthpucker × mouthrolllower	Interaction	0.1	0.592	Pearson	1
browdownright_neutral	Neutral	0.1	0.611	Pearson	1
browdownleft_neutral	Neutral	0.1	0.619	Pearson	1
nosesneerleft	Activated	-0.1	0.629	Pearson	1
browdownleft_delta	Delta	-0.1	0.624	Pearson	1

eyeblinkright	Activated	-0.09	0.653	Pearson	1
jawforward	Activated	-0.09	0.639	Pearson	1
eyelookupleft_delta	Delta	-0.09	0.636	Pearson	1
nosesneerleft_neutral	Neutral	0.09	0.658	Pearson	1
asym_mouthfrownleft_delta	Asymmetry	-0.09	0.648	Pearson	1
mouthleft_neutral	Neutral	-0.09	0.677	Spearman	1
asym_mouthsmileleft	Asymmetry	0.09	0.662	Pearson	1
activated_range	Aggregation	-0.09	0.657	Pearson	1
mouthrolllower × mouthsmileright	Interaction	-0.09	0.657	Pearson	1
asym_mouthfrownleft	Asymmetry	-0.09	0.657	Pearson	1
browdownleft	Activated	-0.08	0.676	Pearson	1
asym_cheeksquintleft	Asymmetry	-0.08	0.676	Pearson	1
asym_mouthdimpleleft_delta	Asymmetry	-0.08	0.686	Pearson	1
mouthupperupleft_neutral	Neutral	0.08	0.69	Pearson	1
eyelookoutleft_neutral	Neutral	-0.08	0.69	Spearman	1
eyelookinleft_neutral	Neutral	0.08	0.685	Pearson	1
mouthrollupper × mouthshrugupper	Interaction	-0.08	0.697	Pearson	1
mouthdimpleright_delta	Delta	0.08	0.693	Pearson	1
asym_mouthleft_neutral	Asymmetry	-0.08	0.7	Pearson	1
asym_mouthdimpleleft	Asymmetry	-0.08	0.704	Pearson	1
mouthpucker_delta	Delta	0.07	0.716	Pearson	1
mouthdimpleleft_delta	Delta	-0.07	0.725	Pearson	1
jawleft_neutral	Neutral	-0.07	0.756	Spearman	1
mouthrollupper × mouthsmileleft	Interaction	-0.07	0.731	Pearson	1
asym_cheeksquintleft_delta	Asymmetry	-0.07	0.732	Pearson	1
mouthfunnel_delta	Delta	0.07	0.743	Pearson	1
mouthright_delta	Delta	-0.07	0.734	Pearson	1
delta_std	Aggregation	-0.07	0.734	Pearson	1

asym_eyelookinleft_neutral	Asymmetry	0.07	0.745	Pearson	1
mouthshrugupper × mouthstretchright	Interaction	-0.06	0.759	Spearman	1
mouthshruglower × mouthsmileright	Interaction	0.06	0.775	Pearson	1
asym_mouthlowerdownleft	Asymmetry	-0.05	0.782	Pearson	1
mouthright	Activated	-0.05	0.778	Pearson	1
mouthrollupper	Activated	-0.05	0.783	Pearson	1
nosesneerright_neutral	Neutral	0.05	0.8	Pearson	1
mouthshruglower × mouthshrugupper	Interaction	0.05	0.793	Pearson	1
eyelookupleft	Activated	-0.05	0.794	Pearson	1
asym_mouthsmileleft_neutral	Asymmetry	-0.05	0.819	Pearson	1
cheekpuff_delta	Delta	-0.05	0.811	Spearman	1
mouthupperupright	Activated	0.04	0.823	Pearson	1
mouthrolllower × mouthshrugupper	Interaction	0.04	0.831	Pearson	1
mouthclose	Activated	0.04	0.831	Pearson	1
cheekpuff	Activated	-0.04	0.833	Spearman	1
asym_eyelookinleft_delta	Asymmetry	-0.04	0.836	Pearson	1
jawright_neutral	Neutral	0.04	0.841	Spearman	1
mouthupperupright_delta	Delta	0.04	0.855	Pearson	1
activated_min	Aggregation	0.04	0.852	Pearson	1
asym_mouthpressleft_delta	Asymmetry	-0.04	0.855	Pearson	1
mouthstretchleft_neutral	Neutral	0.04	0.856	Pearson	1
mouthpressleft	Activated	-0.03	0.862	Pearson	1
mouthpucker × mouthrollupper	Interaction	-0.03	0.864	Pearson	1
activated_std	Aggregation	0.03	0.866	Pearson	1
tongueout_neutral	Neutral	0.03	0.87	Pearson	1
asym_mouthlowerdownleft_delta	Asymmetry	-0.03	0.873	Pearson	1
asym_mouthpressleft	Asymmetry	-0.03	0.879	Pearson	1

cheekpuff_neutral	Neutral	0.03	0.88	Pearson	1
asym_mouthupperupleft	Asymmetry	-0.03	0.884	Pearson	1
cheeksquintright_neutral	Neutral	0.03	0.886	Pearson	1
eyesquintleft	Activated	0.03	0.893	Pearson	1
mouthfunnel	Activated	0.03	0.902	Pearson	1
asym_nosesneerleft_neutral	Asymmetry	-0.02	0.9	Pearson	1
mouthclose_neutral	Neutral	0.02	0.901	Pearson	1
eyeblinkleft	Activated	0.02	0.91	Pearson	1
asym_mouthupperupleft_delta	Asymmetry	-0.02	0.907	Pearson	1
delta_min	Aggregation	-0.02	0.906	Pearson	1
nosesneerright	Activated	-0.02	0.909	Pearson	1
jawforward_delta	Delta	0.02	0.915	Pearson	1
mouthshrugupper × mouthstretchleft	Interaction	-0.02	0.921	Spearman	1
mouthrolllower × mouthrollupper	Interaction	0.02	0.92	Pearson	1
nosesneerright_delta	Delta	0.02	0.919	Pearson	1
eyelookdownleft_delta	Delta	-0.02	0.92	Pearson	1
asym_eyelookinleft	Asymmetry	-0.02	0.921	Pearson	1
eyewideleft_delta	Delta	-0.02	0.924	Pearson	1
activated_max	Aggregation	-0.02	0.935	Pearson	1
mouthpressright	Activated	-0.01	0.939	Pearson	1
mouthclose_delta	Delta	-0.01	0.94	Pearson	1
eyesquintleft_neutral	Neutral	0.01	0.943	Pearson	1
eyesquintright_neutral	Neutral	0.01	0.945	Pearson	1
jawopen_neutral	Neutral	0.01	0.947	Pearson	1
mouthshrugupper × mouthupperupright	Interaction	-0.01	0.946	Pearson	1
mouthrollupper_delta	Delta	0.01	0.951	Pearson	1
mouthpressright_delta	Delta	0.01	0.954	Pearson	1
mouthpressleft_delta	Delta	-0.01	0.954	Pearson	1

asym_eyewideleft_neutral	Asymmetry	-0.01	0.963	Pearson	1
asym_eyelookdownleft_neutral	Asymmetry	0.01	0.966	Pearson	1
mouthfunnel_neutral	Neutral	0.01	0.971	Pearson	1
mouthupperupright_neutral	Neutral	-0.01	0.969	Pearson	1
delta_range	Aggregation	-0.01	0.969	Pearson	1
cheeksquintleft_neutral	Neutral	-0.01	0.976	Pearson	1
eyelookinright_neutral	Neutral	0	0.983	Pearson	1
asym_jawleft_neutral	Asymmetry	-0	0.987	Pearson	1
eyelookupright_delta	Delta	0	0.988	Pearson	1
neutral_min	Aggregation	-	-	Pearson	-

Supplementary Table 5. Feature-level correlations between the 261 engineered facial blendshape features and the MDS-UPDRS III sum score in people with Parkinson's disease. Correlations were residualised for age, sex, and levodopa equivalent dose (LED). Pearson correlation was used when both variables were approximately normal after Tukey outlier removal; Spearman correlation was used otherwise. Multiple testing was controlled within each feature family using *Meff*-based Bonferroni correction.

Supplementary Table 6| Clinical correlations with MDS-UPDRS III item 3.2 (facial expression)

Feature	Feature group	r	p value	Method	p_adj
mouthleft × mouthsmileright	Interaction	-0.65	5.00e-05	Spearman	0.0012
mouthsmileright	Activated	-0.62	1.22e-04	Spearman	0.00473
mouthleft × mouthrolllower	Interaction	-0.55	8.79e-04	Spearman	0.021
mouthshrugupper × mouthsmileright	Interaction	-0.52	0.00206	Spearman	0.05
mouthrolllower × mouthsmileright	Interaction	-0.52	0.00211	Spearman	0.051
mouthleft × mouthshrugupper	Interaction	-0.52	0.00211	Spearman	0.051
mouthsmileright_delta	Delta	-0.49	0.00369	Spearman	0.138
mouthrolllower × mouthsmileleft	Interaction	-0.49	0.00431	Spearman	0.103
mouthleft	Activated	-0.48	0.00512	Spearman	0.199
jawleft	Activated	-0.47	0.00537	Spearman	0.209
mouthlowerdownleft	Activated	-0.47	0.00665	Spearman	0.258

mouthpucker × mouthleft	Interaction	-0.46	0.00732	Spearman	0.176
mouthleft_delta	Delta	-0.46	0.00752	Spearman	0.282
jawleft_delta	Delta	-0.45	0.00868	Spearman	0.325
mouthshrugupper × mouthsmileleft	Interaction	-0.45	0.00913	Spearman	0.219
browouterupleft_delta	Delta	-0.45	0.01	Spearman	0.393
asym_eyewideleft	Asymmetry	0.43	0.027	Spearman	1
asym_eyewideleft_delta	Asymmetry	0.43	0.027	Spearman	1
mouthshruglower_delta	Delta	0.43	0.013	Spearman	0.482
mouthshruglower	Activated	0.43	0.013	Spearman	0.521
eyewideright_delta	Delta	-0.41	0.022	Spearman	0.812
mouthsmileleft	Activated	-0.41	0.019	Spearman	0.719
mouthleft × mouthrollupper	Interaction	-0.41	0.019	Spearman	0.449
mouthrollupper × mouthsmileright	Interaction	-0.4	0.022	Spearman	0.523
eyewideright	Activated	-0.39	0.028	Spearman	1
mouthfrownright	Activated	0.39	0.024	Spearman	0.924
browouterupleft	Activated	-0.39	0.027	Spearman	1
eyewideleft	Activated	-0.39	0.035	Spearman	1
mouthdimpleright	Activated	-0.38	0.033	Spearman	1
mouthshrugupper_delta	Delta	-0.36	0.039	Spearman	1
mouthlowerdownleft_delta	Delta	-0.35	0.051	Spearman	1
mouthrollupper × mouthstretchright	Interaction	-0.34	0.06	Spearman	1
mouthshrugupper	Activated	-0.34	0.057	Spearman	1
mouthpucker × mouthshruglower	Interaction	0.33	0.058	Spearman	1
mouthrollupper × mouthshrugupper	Interaction	-0.33	0.067	Spearman	1
asym_nosesneerleft_neutral	Asymmetry	-0.33	0.067	Spearman	1
browinnerup_delta	Delta	-0.32	0.069	Spearman	1
asym_eyeblinkleft_neutral	Asymmetry	-0.32	0.104	Spearman	1
eyeblinkleft_delta	Delta	0.32	0.086	Spearman	1

mouthpucker × mouthshrugupper	Interaction	-0.32	0.074	Spearman	1
mouthfrownright_delta	Delta	0.31	0.076	Spearman	1
mouthshrugupper × mouthstretchright	Interaction	-0.31	0.082	Spearman	1
eyelookdownleft_neutral	Neutral	-0.31	0.079	Spearman	1
eyelookdownright_neutral	Neutral	-0.31	0.079	Spearman	1
browdownright	Activated	-0.31	0.079	Spearman	1
mouthrollupper × mouthsmileleft	Interaction	-0.31	0.081	Spearman	1
browinnerup	Activated	-0.31	0.084	Spearman	1
asym_mouthpressleft_delta	Asymmetry	0.3	0.086	Spearman	1
mouthrolllower	Activated	-0.3	0.093	Spearman	1
mouthstretchleft	Activated	-0.3	0.088	Spearman	1
asym_eyeblinkleft_delta	Asymmetry	-0.3	0.119	Spearman	1
asym_eyeblinkleft	Asymmetry	-0.3	0.119	Spearman	1
asym_mouthpressleft	Asymmetry	0.3	0.092	Spearman	1
mouthrolllower_neutral	Neutral	-0.29	0.103	Spearman	1
mouthdimpleleft_neutral	Neutral	-0.29	0.103	Spearman	1
mouthsmileleft_delta	Delta	-0.29	0.098	Spearman	1
mouthdimpleright_neutral	Neutral	-0.29	0.104	Spearman	1
mouthrolllower × mouthshrugupper	Interaction	-0.29	0.101	Spearman	1
asym_mouthleft_delta	Asymmetry	-0.29	0.115	Spearman	1
eyewideleft_delta	Delta	-0.29	0.11	Spearman	1
activated_mean	Aggregation	-0.29	0.111	Spearman	0.885
asym_browdownleft	Asymmetry	0.28	0.109	Spearman	1
asym_browdownleft_delta	Asymmetry	0.28	0.109	Spearman	1
asym_mouthleft	Asymmetry	-0.28	0.122	Spearman	1
asym_mouthfrownleft_delta	Asymmetry	-0.28	0.119	Spearman	1
mouthdimpleright_delta	Delta	-0.27	0.135	Spearman	1
asym_eyesquintleft_neutral	Asymmetry	0.27	0.136	Spearman	1

mouthfunnel	Activated	0.27	0.144	Spearman	1
mouthlowerdownleft_neutral	Neutral	-0.27	0.124	Spearman	1
mouthshrugupper × mouthstretchleft	Interaction	-0.27	0.139	Spearman	1
asym_jawleft	Asymmetry	-0.27	0.134	Spearman	1
asym_eyelookupleft	Asymmetry	-0.27	0.149	Spearman	1
asym_eyelookupleft_delta	Asymmetry	-0.27	0.149	Spearman	1
delta_mean	Aggregation	-0.27	0.13	Spearman	1
mouthrolllower × mouthrollupper	Interaction	-0.27	0.14	Spearman	1
asym_mouthsmileleft_delta	Asymmetry	0.27	0.143	Spearman	1
neutral_mean	Aggregation	-0.27	0.143	Spearman	1
asym_nosesneerleft_delta	Asymmetry	0.26	0.175	Spearman	1
eyelookupright_neutral	Neutral	-0.26	0.197	Spearman	1
eyelookupleft_neutral	Neutral	-0.26	0.197	Spearman	1
asym_mouthsmileleft	Asymmetry	0.26	0.148	Spearman	1
cheekpuff_neutral	Neutral	0.26	0.146	Spearman	1
asym_mouthfrownleft	Asymmetry	-0.25	0.156	Spearman	1
eyeblinkright_delta	Delta	0.25	0.183	Spearman	1
asym_mouthfrownleft_neutral	Asymmetry	0.24	0.185	Spearman	1
eyelookupright	Activated	-0.24	0.191	Spearman	1
mouthstretchleft_delta	Delta	-0.24	0.182	Spearman	1
asym_eyelookinleft_delta	Asymmetry	-0.24	0.186	Spearman	1
cheeksquintright_delta	Delta	-0.24	0.187	Spearman	1
asym_nosesneerleft	Asymmetry	0.23	0.229	Spearman	1
mouthpucker × mouthrolllower	Interaction	-0.23	0.207	Spearman	1
mouthrollupper	Activated	-0.22	0.21	Spearman	1
browouterupright_delta	Delta	-0.22	0.223	Spearman	1
eyelookupleft_delta	Delta	-0.22	0.215	Spearman	1
nosesneerleft_delta	Delta	0.22	0.22	Spearman	1

eyewideleft_neutral	Neutral	0.22	0.222	Spearman	1
eyewideright_neutral	Neutral	0.22	0.222	Spearman	1
mouthpucker × mouthrollupper	Interaction	-0.21	0.232	Spearman	1
cheeksquintleft_delta	Delta	-0.21	0.235	Spearman	1
asym_jawleft_delta	Asymmetry	-0.21	0.274	Spearman	1
cheeksquintleft	Activated	-0.21	0.25	Spearman	1
mouthstretchright_delta	Delta	-0.2	0.263	Spearman	1
asym_mouthleft_neutral	Asymmetry	-0.2	0.272	Spearman	1
eyelookdownright_delta	Delta	0.2	0.263	Spearman	1
mouthrollupper × mouthshruglower	Interaction	-0.2	0.274	Spearman	1
asym_mouthlowerdownleft	Asymmetry	-0.2	0.283	Spearman	1
browdownright_delta	Delta	-0.2	0.276	Spearman	1
browdownleft_neutral	Neutral	-0.19	0.286	Spearman	1
mouthpressleft	Activated	0.19	0.281	Spearman	1
eyeblinkright	Activated	0.19	0.321	Spearman	1
eyelookupright_delta	Delta	-0.19	0.293	Spearman	1
asym_mouthlowerdownleft_neutral	Asymmetry	-0.19	0.314	Spearman	1
browdownright_neutral	Neutral	-0.19	0.308	Spearman	1
mouthleft × mouthshruglower	Interaction	-0.18	0.303	Spearman	1
mouthpressright_neutral	Neutral	-0.18	0.377	Spearman	1
delta_std	Aggregation	-0.18	0.322	Spearman	1
eyelookinright_neutral	Neutral	-0.18	0.341	Spearman	1
eyelookupleft	Activated	-0.18	0.327	Spearman	1
asym_eyelookinleft_neutral	Asymmetry	0.17	0.348	Spearman	1
mouthright_delta	Delta	-0.17	0.336	Spearman	1
jawforward_delta	Delta	0.16	0.371	Spearman	1
eyelookdownright	Activated	-0.16	0.373	Spearman	1
mouthpressleft_delta	Delta	0.16	0.366	Spearman	1
mouthsmileright_neutral	Neutral	0.16	0.402	Spearman	1

asym_mouthlowerdownleft_delta	Asymmetry	-0.16	0.387	Spearman	1
asym_mouthupperupleft_neutral	Asymmetry	-0.16	0.413	Spearman	1
mouthrolllower × mouthshruglower	Interaction	0.16	0.38	Spearman	1
eyelookinleft_delta	Delta	-0.16	0.383	Spearman	1
eyelookdownleft	Activated	-0.16	0.383	Spearman	1
mouthpressleft_neutral	Neutral	-0.16	0.408	Spearman	1
asym_browouterupleft_delta	Asymmetry	-0.16	0.42	Spearman	1
asym_browouterupleft	Asymmetry	-0.16	0.42	Spearman	1
mouthfunnel_delta	Delta	0.15	0.407	Spearman	1
asym_cheeksquintleft	Asymmetry	0.15	0.401	Spearman	1
jawopen	Activated	-0.15	0.436	Spearman	1
asym_eyesquintleft_delta	Asymmetry	-0.15	0.412	Spearman	1
asym_eyesquintleft	Asymmetry	-0.15	0.412	Spearman	1
mouthright	Activated	-0.15	0.403	Spearman	1
browouterupright	Activated	-0.15	0.413	Spearman	1
mouthstretchleft_neutral	Neutral	-0.15	0.41	Spearman	1
mouthrollupper_delta	Delta	-0.15	0.412	Spearman	1
asym_mouthdimpleleft_delta	Asymmetry	0.15	0.423	Spearman	1
mouthdimpleleft	Activated	-0.15	0.426	Spearman	1
asym_mouthdimpleleft	Asymmetry	0.15	0.427	Spearman	1
mouthlowerdownright_neutral	Neutral	-0.14	0.461	Spearman	1
mouthshrugupper × mouthupperupright	Interaction	-0.13	0.456	Spearman	1
eyesquintright_delta	Delta	-0.13	0.462	Spearman	1
eyesquintright	Activated	-0.13	0.462	Spearman	1
eyelookoutright_delta	Delta	-0.13	0.465	Spearman	1
mouthupperupleft_delta	Delta	0.13	0.481	Spearman	1
activated_min	Aggregation	-0.13	0.467	Spearman	1
eyelookoutright_neutral	Neutral	0.13	0.509	Spearman	1

asym_eyelookoutleft_neutral	Asymmetry	-0.13	0.511	Spearman	1
jawright	Activated	-0.13	0.475	Spearman	1
mouthstretchright_neutral	Neutral	-0.13	0.482	Spearman	1
eyelookinleft_neutral	Neutral	0.12	0.495	Spearman	1
eyelookoutleft	Activated	-0.12	0.506	Spearman	1
browdownleft_delta	Delta	0.12	0.494	Spearman	1
mouthfrownleft	Activated	0.12	0.5	Spearman	1
asym_eyelookoutleft_delta	Asymmetry	0.12	0.502	Spearman	1
asym_cheeksquintleft_delta	Asymmetry	0.12	0.511	Spearman	1
eyelookoutleft_delta	Delta	-0.12	0.52	Spearman	1
cheeksquintleft_neutral	Neutral	-0.12	0.515	Spearman	1
nosesneerright_delta	Delta	0.12	0.518	Spearman	1
eyelookinleft	Activated	-0.11	0.525	Spearman	1
mouthupperupright	Activated	0.11	0.533	Spearman	1
eyelookoutright	Activated	-0.11	0.533	Spearman	1
mouthfrownleft_neutral	Neutral	0.11	0.554	Spearman	1
mouthlowerdownright_delta	Delta	0.11	0.592	Spearman	1
mouthfrownleft_delta	Delta	0.11	0.55	Spearman	1
eyesquintleft_delta	Delta	-0.11	0.555	Spearman	1
asym_eyelookoutleft	Asymmetry	0.1	0.566	Spearman	1
jawright_delta	Delta	-0.1	0.566	Spearman	1
mouthclose	Activated	0.1	0.579	Spearman	1
mouthsmileleft_neutral	Neutral	-0.1	0.605	Spearman	1
asym_mouthstretchleft_delta	Asymmetry	0.1	0.6	Spearman	1
cheeksquintright_neutral	Neutral	-0.1	0.6	Spearman	1
asym_mouthsmileleft_neutral	Asymmetry	-0.1	0.626	Spearman	1
eyelookinright_delta	Delta	-0.1	0.611	Spearman	1
mouthupperupright_delta	Delta	0.09	0.605	Spearman	1
asym_eyelookinleft	Asymmetry	-0.09	0.607	Spearman	1

mouthfrownright_neutral	Neutral	0.09	0.626	Spearman	1
mouthright_neutral	Neutral	0.09	0.634	Spearman	1
mouthstretchright	Activated	-0.09	0.623	Spearman	1
mouthshruglower × mouthsmileleft	Interaction	-0.09	0.634	Spearman	1
asym_mouthupperupleft	Asymmetry	-0.09	0.642	Spearman	1
asym_mouthupperupleft_delta	Asymmetry	-0.09	0.644	Spearman	1
asym_mouthstretchleft_neutral	Asymmetry	-0.08	0.652	Spearman	1
activated_range	Aggregation	0.08	0.653	Spearman	1
delta_max	Aggregation	-0.08	0.673	Spearman	1
nosesneerleft	Activated	0.08	0.67	Spearman	1
jawforward_neutral	Neutral	-0.08	0.676	Spearman	1
mouthpucker	Activated	-0.08	0.683	Spearman	1
nosesneerleft_neutral	Neutral	-0.08	0.682	Spearman	1
neutral_std	Aggregation	-0.08	0.673	Spearman	1
mouthpressright_delta	Delta	-0.08	0.676	Spearman	1
mouthshruglower × mouthshrugupper	Interaction	-0.07	0.682	Spearman	1
browdownleft	Activated	0.07	0.683	Spearman	1
mouthshruglower_neutral	Neutral	-0.07	0.689	Spearman	1
asym_cheeksquintleft_neutral	Asymmetry	-0.07	0.714	Spearman	1
asym_browouterupleft_neutral	Asymmetry	0.07	0.716	Spearman	1
eyelookdownleft_delta	Delta	0.06	0.72	Spearman	1
mouthdimpleleft_delta	Delta	-0.06	0.739	Spearman	1
mouthshruglower × mouthsmileright	Interaction	-0.06	0.744	Spearman	1
mouthleft_neutral	Neutral	-0.06	0.765	Spearman	1
eyelookoutleft_neutral	Neutral	-0.06	0.754	Spearman	1
cheekpuff	Activated	0.06	0.755	Spearman	1
mouthclose_neutral	Neutral	-0.06	0.756	Spearman	1
mouthpressright	Activated	-0.06	0.761	Spearman	1

jawleft_neutral	Neutral	0.05	0.786	Spearman	1
eyelookinright	Activated	-0.05	0.784	Spearman	1
cheeksquintright	Activated	-0.05	0.793	Spearman	1
mouthshrugupper_neutral	Neutral	-0.05	0.788	Spearman	1
activated_max	Aggregation	-0.05	0.794	Spearman	1
jawforward	Activated	0.05	0.793	Spearman	1
eyeblinkleft	Activated	0.05	0.804	Spearman	1
tongueout_neutral	Neutral	0.05	0.802	Spearman	1
neutral_range	Aggregation	-0.04	0.807	Spearman	1
neutral_max	Aggregation	-0.04	0.807	Spearman	1
mouthupperupleft	Activated	0.04	0.819	Spearman	1
mouthrolllower_delta	Delta	-0.04	0.825	Spearman	1
mouthrollupper_neutral	Neutral	-0.04	0.831	Spearman	1
asym_mouthpressleft_neutral	Asymmetry	0.04	0.828	Spearman	1
asym_jawleft_neutral	Asymmetry	0.04	0.837	Spearman	1
delta_range	Aggregation	-0.04	0.839	Spearman	1
nosesneerright_neutral	Neutral	0.04	0.849	Spearman	1
delta_min	Aggregation	0.04	0.843	Spearman	1
mouthupperupright_neutral	Neutral	-0.04	0.846	Spearman	1
jawopen_neutral	Neutral	-0.03	0.853	Spearman	1
mouthpucker_delta	Delta	-0.03	0.856	Spearman	1
eyesquintright_neutral	Neutral	-0.03	0.856	Spearman	1
eyesquintleft_neutral	Neutral	-0.03	0.856	Spearman	1
mouthclose_delta	Delta	-0.03	0.859	Spearman	1
eyesquintleft	Activated	-0.03	0.863	Spearman	1
asym_browdownleft_neutral	Asymmetry	-0.03	0.875	Spearman	1
asym_mouthdimpleleft_neutral	Asymmetry	-0.03	0.873	Spearman	1
browinnerup_neutral	Neutral	0.03	0.882	Spearman	1
asym_mouthstretchleft	Asymmetry	0.03	0.889	Spearman	1

asym_eyelookdownleft	Asymmetry	-0.02	0.911	Spearman	1
asym_eyelookdownleft_delta	Asymmetry	-0.02	0.911	Spearman	1
jawright_neutral	Neutral	-0.02	0.919	Spearman	1
asym_eyelookupleft_neutral	Asymmetry	-0.02	0.927	Spearman	1
cheekpuff_delta	Delta	0.02	0.924	Spearman	1
jawopen_delta	Delta	0.01	0.949	Spearman	1
nosesneerright	Activated	0.01	0.947	Spearman	1
mouthupperupleft_neutral	Neutral	0.01	0.956	Spearman	1
activated_std	Aggregation	-0.01	0.956	Spearman	1
tongueout	Activated	-0.01	0.96	Spearman	1
tongueout_delta	Delta	-0.01	0.96	Spearman	1
mouthshruglower × mouthstretchright	Interaction	-0.01	0.957	Spearman	1
browouterupright_neutral	Neutral	-0.01	0.97	Spearman	1
browouterupleft_neutral	Neutral	-0.01	0.97	Spearman	1
asym_eyelookdownleft_neutral	Asymmetry	0.01	0.968	Spearman	1
mouthfunnel_neutral	Neutral	0.01	0.972	Spearman	1
mouthshruglower × mouthstretchleft	Interaction	0.01	0.971	Spearman	1
mouthlowerdownright	Activated	-0.01	0.977	Spearman	1
eyeblinkright_neutral	Neutral	-0	0.986	Spearman	1
eyeblinkleft_neutral	Neutral	-0	0.99	Spearman	1
mouthpucker_neutral	Neutral	0	0.993	Spearman	1
asym_eyewideleft_neutral	Asymmetry	0	0.996	Spearman	1
neutral_min	Aggregation	-	-	Spearman	-

Supplementary Table 6. Feature-level correlations between facial blendshape features and MDS-UPDRS III item 3.2 (facial expression) in people with Parkinson's disease.

Supplementary Table 7| Clinical correlations with MDS-UPDRS IV sum score

Feature	Feature group	r	p value	Method	p_adj
asym_mouthleft_delta	Asymmetry	-0.47	0.00721	Spearman	0.302
mouthsmileleft	Activated	-0.46	0.00772	Spearman	0.3
nosesneerright_delta	Delta	0.45	0.00782	Spearman	0.293
asym_mouthleft	Asymmetry	-0.45	0.011	Spearman	0.462
mouthright	Activated	0.44	0.00992	Spearman	0.385
jawopen	Activated	-0.42	0.026	Spearman	1
mouthright_delta	Delta	0.42	0.015	Spearman	0.573
tongueout_delta	Delta	-0.42	0.024	Spearman	0.899
tongueout	Activated	-0.42	0.024	Spearman	0.931
asym_mouthdimpleleft_delta	Asymmetry	-0.41	0.018	Spearman	0.764
browouterupleft_delta	Delta	-0.41	0.019	Spearman	0.713
mouthrolllower × mouthsmileleft	Interaction	-0.41	0.021	Spearman	0.503
asym_mouthdimpleleft	Asymmetry	-0.4	0.025	Spearman	1
jawopen_delta	Delta	-0.39	0.032	Spearman	1
nosesneerright	Activated	0.39	0.026	Spearman	1
mouthsmileleft_delta	Delta	-0.38	0.028	Spearman	1
jawforward_neutral	Neutral	-0.37	0.043	Spearman	1
mouthshrugupper × mouthsmileleft	Interaction	-0.35	0.048	Spearman	1
browouterupleft	Activated	-0.35	0.052	Spearman	1
jawforward	Activated	-0.34	0.058	Spearman	1
mouthupperupright_delta	Delta	0.33	0.066	Spearman	1
mouthupperupright	Activated	0.32	0.072	Spearman	1
browdownright_neutral	Neutral	0.32	0.078	Spearman	1
browdownleft_neutral	Neutral	0.31	0.082	Spearman	1
eyewideright_delta	Delta	-0.31	0.088	Spearman	1
asym_eyesquintleft	Asymmetry	0.31	0.095	Pearson	1

asym_eyesquintleft_delta	Asymmetry	0.31	0.095	Pearson	1
mouthstretchleft_neutral	Neutral	-0.3	0.085	Spearman	1
jawforward_delta	Delta	-0.29	0.102	Spearman	1
eyeblinkright	Activated	-0.29	0.132	Spearman	1
mouthshruglower × mouthsmileright	Interaction	0.28	0.116	Spearman	1
mouthstretchright_delta	Delta	0.28	0.118	Spearman	1
browouterupright_delta	Delta	-0.27	0.128	Spearman	1
jawopen_neutral	Neutral	-0.27	0.142	Spearman	1
asym_browouterupleft	Asymmetry	-0.26	0.176	Spearman	1
asym_browouterupleft_delta	Asymmetry	-0.26	0.176	Spearman	1
mouthclose_neutral	Neutral	-0.26	0.148	Spearman	1
mouthshrugupper × mouthstretchleft	Interaction	-0.25	0.171	Spearman	1
cheeksquintleft_neutral	Neutral	-0.25	0.165	Spearman	1
eyewideright	Activated	-0.25	0.175	Spearman	1
browouterupleft_neutral	Neutral	0.25	0.193	Spearman	1
browouterupright_neutral	Neutral	0.25	0.193	Spearman	1
asym_eyeblinkleft_neutral	Asymmetry	-0.24	0.221	Pearson	1
browinnerup	Activated	-0.24	0.177	Spearman	1
mouthleft × mouthshruglower	Interaction	0.24	0.179	Spearman	1
asym_mouthsmileleft_neutral	Asymmetry	0.24	0.221	Pearson	1
mouthshrugupper_delta	Delta	-0.24	0.184	Spearman	1
cheeksquintright_neutral	Neutral	-0.24	0.191	Spearman	1
nosesneerleft_neutral	Neutral	-0.24	0.2	Spearman	1
mouthrolllower_neutral	Neutral	-0.24	0.193	Spearman	1
browinnerup_delta	Delta	-0.24	0.186	Spearman	1
asym_eyewideleft_delta	Asymmetry	0.23	0.248	Pearson	1
asym_eyewideleft	Asymmetry	0.23	0.248	Pearson	1
mouthlowerdownleft_neutral	Neutral	-0.23	0.191	Spearman	1

browouterupright	Activated	-0.23	0.199	Spearman	1
mouthsmileright_neutral	Neutral	-0.23	0.218	Spearman	1
mouthstretchright_neutral	Neutral	-0.23	0.202	Spearman	1
asym_mouthfrownleft	Asymmetry	-0.23	0.205	Spearman	1
browdownleft	Activated	0.22	0.212	Spearman	1
mouthpressleft_neutral	Neutral	0.22	0.239	Spearman	1
mouthshruglower_delta	Delta	0.22	0.226	Spearman	1
mouthpucker × mouthshrugupper	Interaction	-0.22	0.229	Spearman	1
nosesneerleft_delta	Delta	0.21	0.23	Spearman	1
mouthsmileleft_neutral	Neutral	-0.21	0.247	Spearman	1
neutral_max	Aggregation	0.21	0.233	Spearman	1
neutral_range	Aggregation	0.21	0.233	Spearman	1
mouthpressright_neutral	Neutral	0.21	0.298	Spearman	1
mouthshrugupper	Activated	-0.21	0.248	Spearman	1
asym_mouthfrownleft_delta	Asymmetry	-0.21	0.25	Spearman	1
asym_mouthupperupleft_delta	Asymmetry	-0.21	0.259	Spearman	1
mouthfrownleft_neutral	Neutral	0.2	0.27	Spearman	1
asym_mouthupperupleft	Asymmetry	-0.2	0.263	Spearman	1
mouthclose_delta	Delta	-0.2	0.267	Spearman	1
activated_mean	Aggregation	-0.2	0.268	Spearman	1
mouthupperupleft_delta	Delta	0.2	0.278	Spearman	1
asym_mouthfrownleft_neutral	Asymmetry	0.2	0.279	Spearman	1
mouthfrownright_neutral	Neutral	0.2	0.282	Spearman	1
nosesneerright_neutral	Neutral	-0.2	0.292	Pearson	1
mouthrolllower × mouthshrugupper	Interaction	-0.2	0.271	Spearman	1
mouthlowerdownright_neutral	Neutral	-0.2	0.287	Pearson	1
mouthshrugupper × mouthsmileright	Interaction	-0.19	0.281	Spearman	1
eyelookoutright_neutral	Neutral	0.19	0.329	Spearman	1

mouthrollupper × mouthstretchright	Interaction	0.19	0.311	Spearman	1
jawright_neutral	Neutral	0.19	0.299	Spearman	1
asym_mouthsmileleft_delta	Asymmetry	-0.18	0.316	Spearman	1
cheekpuff	Activated	-0.18	0.317	Spearman	1
asym_eyelookoutleft_neutral	Asymmetry	-0.18	0.364	Spearman	1
asym_eyelookdownleft_delta	Asymmetry	0.18	0.329	Spearman	1
asym_eyelookdownleft	Asymmetry	0.18	0.329	Spearman	1
asym_eyelookinleft_delta	Asymmetry	-0.17	0.331	Spearman	1
asym_mouthpressleft_delta	Asymmetry	-0.17	0.332	Spearman	1
asym_eyewideleft_neutral	Asymmetry	-0.17	0.38	Spearman	1
asym_mouthlowerdownleft	Asymmetry	-0.17	0.355	Spearman	1
eyelookinleft_delta	Delta	-0.17	0.351	Spearman	1
mouthshruglower	Activated	0.17	0.353	Spearman	1
asym_browdownleft_delta	Asymmetry	0.17	0.355	Spearman	1
asym_browdownleft	Asymmetry	0.17	0.355	Spearman	1
delta_std	Aggregation	-0.17	0.355	Spearman	1
asym_mouthlowerdownleft_delta	Asymmetry	-0.17	0.366	Spearman	1
mouthrolllower × mouthsmileright	Interaction	-0.16	0.36	Spearman	1
cheekpuff_delta	Delta	-0.16	0.361	Spearman	1
mouthfunnel	Activated	-0.16	0.389	Spearman	1
mouthstretchright	Activated	0.16	0.365	Spearman	1
mouthrolllower	Activated	-0.16	0.379	Spearman	1
asym_mouthpressleft	Asymmetry	-0.16	0.376	Spearman	1
mouthfrownleft	Activated	-0.16	0.377	Spearman	1
mouthpucker	Activated	0.16	0.406	Spearman	1
mouthclose	Activated	-0.15	0.39	Spearman	1
mouthupperupleft_neutral	Neutral	-0.15	0.401	Spearman	1
browdownright_delta	Delta	-0.15	0.395	Spearman	1

asym_mouthpressleft_neutral	Asymmetry	0.15	0.396	Spearman	1
asym_mouthsmileleft	Asymmetry	-0.15	0.409	Spearman	1
mouthpucker_delta	Delta	0.15	0.418	Spearman	1
mouthupperupleft	Activated	0.15	0.423	Spearman	1
mouthpucker × mouthshruglower	Interaction	0.14	0.424	Spearman	1
mouthleft_neutral	Neutral	0.14	0.463	Spearman	1
eyelookinleft_neutral	Neutral	0.14	0.439	Spearman	1
mouthpressright	Activated	0.14	0.431	Spearman	1
mouthshruglower × mouthshrugupper	Interaction	-0.14	0.439	Spearman	1
mouthpucker × mouthrolllower	Interaction	-0.14	0.447	Spearman	1
eyesquintleft_delta	Delta	0.14	0.453	Spearman	1
activated_max	Aggregation	-0.14	0.469	Spearman	1
cheeksquintleft	Activated	-0.13	0.467	Spearman	1
cheekpuff_neutral	Neutral	0.13	0.469	Spearman	1
activated_std	Aggregation	-0.13	0.471	Spearman	1
eyewideleft	Activated	-0.13	0.494	Pearson	1
mouthrolllower_delta	Delta	-0.13	0.474	Spearman	1
nosesneerleft	Activated	0.13	0.487	Spearman	1
mouthlowerdownright	Activated	-0.13	0.504	Spearman	1
mouthshrugupper_neutral	Neutral	0.13	0.494	Spearman	1
mouthrollupper × mouthshrugupper	Interaction	0.12	0.503	Spearman	1
mouthrolllower × mouthrollupper	Interaction	0.12	0.495	Spearman	1
asym_eyelookinleft_neutral	Asymmetry	0.12	0.503	Spearman	1
eyelookinright_neutral	Neutral	-0.12	0.506	Spearman	1
activated_min	Aggregation	0.12	0.492	Spearman	1
delta_max	Aggregation	-0.12	0.51	Spearman	1
mouthright_neutral	Neutral	0.12	0.51	Spearman	1
asym_browouterupleft_neutral	Asymmetry	-0.12	0.511	Spearman	1

cheeksquintright	Activated	-0.12	0.54	Pearson	1
mouthupperupright_neutral	Neutral	-0.12	0.504	Spearman	1
asym_jawleft_neutral	Asymmetry	-0.12	0.511	Spearman	1
eyelookdownright_delta	Delta	0.12	0.505	Spearman	1
eyelookdownright	Activated	-0.12	0.513	Spearman	1
delta_min	Aggregation	0.12	0.514	Spearman	1
asym_eyeblinkleft_delta	Asymmetry	0.12	0.555	Spearman	1
asym_eyeblinkleft	Asymmetry	0.12	0.555	Spearman	1
eyelookdownleft_neutral	Neutral	-0.12	0.519	Spearman	1
eyelookupleft	Activated	0.12	0.519	Spearman	1
eyelookdownright_neutral	Neutral	-0.12	0.519	Spearman	1
eyesquintright_delta	Delta	-0.12	0.522	Spearman	1
neutral_std	Aggregation	0.11	0.533	Spearman	1
asym_nosesneerleft_neutral	Asymmetry	-0.11	0.542	Spearman	1
cheeksquintleft_delta	Delta	-0.11	0.541	Spearman	1
mouthdimpleleft	Activated	-0.11	0.554	Spearman	1
asym_nosesneerleft	Asymmetry	-0.11	0.585	Spearman	1
mouthsmileright	Activated	-0.11	0.551	Spearman	1
asym_nosesneerleft_delta	Asymmetry	-0.11	0.587	Spearman	1
asym_eyelookinleft	Asymmetry	-0.11	0.555	Spearman	1
asym_mouthstretchleft	Asymmetry	-0.11	0.565	Spearman	1
asym_mouthleft_neutral	Asymmetry	-0.1	0.575	Spearman	1
mouthleft × mouthrollupper	Interaction	0.1	0.567	Spearman	1
mouthfunnel_neutral	Neutral	0.1	0.597	Spearman	1
asym_eyelookupleft	Asymmetry	-0.1	0.593	Spearman	1
asym_eyelookupleft_delta	Asymmetry	-0.1	0.593	Spearman	1
mouthrollupper_delta	Delta	0.1	0.574	Spearman	1
activated_range	Aggregation	-0.1	0.576	Spearman	1
eyelookinleft	Activated	-0.1	0.584	Spearman	1

asym_eyelookdownleft_neutral	Asymmetry	0.1	0.586	Spearman	1
mouthpucker × mouthrollupper	Interaction	0.1	0.592	Spearman	1
mouthrollupper_neutral	Neutral	-0.1	0.609	Spearman	1
mouthdimpleleft_delta	Delta	-0.09	0.608	Spearman	1
jawleft	Activated	-0.09	0.623	Spearman	1
eyewideleft_neutral	Neutral	0.09	0.624	Spearman	1
eyewideright_neutral	Neutral	0.09	0.624	Spearman	1
mouthfrownleft_delta	Delta	-0.09	0.625	Spearman	1
mouthpressright_delta	Delta	0.09	0.636	Spearman	1
mouthpucker_neutral	Neutral	-0.09	0.654	Spearman	1
eyelookdownleft_delta	Delta	0.08	0.645	Spearman	1
asym_jawleft	Asymmetry	-0.08	0.654	Spearman	1
mouthrollupper × mouthshruglower	Interaction	0.08	0.658	Spearman	1
eyesquintleft	Activated	0.08	0.675	Pearson	1
asym_eyelookoutleft	Asymmetry	-0.08	0.671	Spearman	1
mouthrollupper × mouthsmileright	Interaction	0.08	0.671	Spearman	1
eyelookinright	Activated	-0.08	0.69	Spearman	1
asym_mouthupperupleft_neutral	Asymmetry	0.07	0.702	Pearson	1
asym_browdownleft_neutral	Asymmetry	-0.07	0.718	Pearson	1
mouthrollupper	Activated	0.07	0.691	Spearman	1
mouthdimpleright	Activated	0.07	0.699	Spearman	1
delta_range	Aggregation	-0.07	0.698	Spearman	1
asym_cheeksquintleft_delta	Asymmetry	-0.07	0.703	Spearman	1
mouthstretchleft	Activated	-0.07	0.703	Spearman	1
asym_mouthdimpleleft_neutral	Asymmetry	-0.07	0.714	Spearman	1
cheeksquintright_delta	Delta	0.07	0.712	Spearman	1
eyewideleft_delta	Delta	-0.07	0.721	Spearman	1
mouthleft × mouthshrugupper	Interaction	-0.07	0.717	Spearman	1
browdownright	Activated	0.06	0.724	Spearman	1

delta_mean	Aggregation	-0.06	0.731	Spearman	1
mouthrollupper × mouthsmileleft	Interaction	-0.06	0.731	Spearman	1
eyesquintright_neutral	Neutral	0.06	0.732	Spearman	1
eyesquintleft_neutral	Neutral	0.06	0.732	Spearman	1
asym_mouthlowerdownleft_neutral	Asymmetry	0.06	0.745	Spearman	1
mouthfrownright	Activated	0.06	0.748	Spearman	1
browdownleft_delta	Delta	0.06	0.756	Spearman	1
jawright_delta	Delta	0.05	0.765	Spearman	1
mouthlowerdownleft_delta	Delta	0.05	0.77	Spearman	1
mouthfrownright_delta	Delta	0.05	0.768	Spearman	1
eyelookupleft_neutral	Neutral	-0.05	0.797	Spearman	1
eyelookupright_neutral	Neutral	-0.05	0.797	Spearman	1
eyeblinkleft_neutral	Neutral	-0.05	0.774	Spearman	1
eyeblinkright_neutral	Neutral	-0.05	0.777	Spearman	1
jawleft_delta	Delta	-0.05	0.776	Spearman	1
browinnerup_neutral	Neutral	-0.05	0.788	Pearson	1
asym_cheeksquintleft_neutral	Asymmetry	0.05	0.792	Pearson	1
asym_cheeksquintleft	Asymmetry	-0.05	0.791	Spearman	1
jawright	Activated	0.05	0.789	Spearman	1
eyelookoutright_delta	Delta	-0.05	0.795	Spearman	1
eyelookupleft_delta	Delta	0.05	0.799	Spearman	1
jawleft_neutral	Neutral	-0.05	0.818	Spearman	1
eyelookoutleft_neutral	Neutral	-0.05	0.806	Spearman	1
asym_mouthstretchleft_neutral	Asymmetry	-0.04	0.813	Spearman	1
eyelookoutleft_delta	Delta	0.04	0.823	Spearman	1
eyeblinkright_delta	Delta	-0.04	0.827	Spearman	1
eyesquintright	Activated	-0.04	0.83	Spearman	1
mouthshruglower_neutral	Neutral	0.04	0.836	Spearman	1
mouthsmileright_delta	Delta	-0.04	0.835	Spearman	1

eyelookoutleft	Activated	0.04	0.841	Spearman	1
mouthdimpleleft_neutral	Neutral	0.04	0.845	Spearman	1
asym_eyelookupleft_neutral	Asymmetry	-0.04	0.855	Spearman	1
asym_eyelookoutleft_delta	Asymmetry	-0.04	0.845	Spearman	1
mouthfunnel_delta	Delta	0.03	0.856	Spearman	1
mouthleft × mouthrolllower	Interaction	-0.03	0.852	Spearman	1
asym_mouthstretchleft_delta	Asymmetry	-0.03	0.855	Spearman	1
mouthlowerdownleft	Activated	-0.03	0.858	Spearman	1
mouthshrugupper × mouthstretchright	Interaction	0.03	0.87	Spearman	1
eyeblinkleft	Activated	0.03	0.879	Spearman	1
eyelookupright	Activated	0.03	0.88	Pearson	1
eyeblinkleft_delta	Delta	0.03	0.882	Spearman	1
mouthdimpleright_neutral	Neutral	-0.03	0.878	Spearman	1
mouthlowerdownright_delta	Delta	0.02	0.903	Pearson	1
mouthpressleft_delta	Delta	-0.02	0.897	Spearman	1
mouthpucker × mouthleft	Interaction	0.02	0.903	Spearman	1
eyelookinright_delta	Delta	0.02	0.918	Spearman	1
mouthleft_delta	Delta	-0.02	0.919	Spearman	1
eyelookdownleft	Activated	-0.02	0.921	Spearman	1
mouthrolllower × mouthshruglower	Interaction	0.02	0.922	Spearman	1
mouthstretchleft_delta	Delta	0.02	0.929	Spearman	1
tongueout_neutral	Neutral	-0.01	0.941	Spearman	1
mouthleft	Activated	-0.01	0.941	Spearman	1
mouthshruglower × mouthstretchright	Interaction	-0.01	0.962	Spearman	1
mouthdimpleright_delta	Delta	-0.01	0.975	Pearson	1
mouthpressleft	Activated	0.01	0.975	Spearman	1
eyelookoutright	Activated	-0.01	0.975	Spearman	1
mouthshruglower × mouthsmileleft	Interaction	0.01	0.976	Spearman	1

mouthshruglower × mouthstretchleft	Interaction	0	0.979	Spearman	1
mouthshrugupper × mouthupperupright	Interaction	-0	0.985	Spearman	1
neutral_mean	Aggregation	-0	0.987	Spearman	1
mouthleft × mouthsmileright	Interaction	0	0.988	Spearman	1
eyelookupright_delta	Delta	-0	0.996	Spearman	1
asym_jawleft_delta	Asymmetry	6.67e-04	0.997	Spearman	1
asym_eyesquintleft_neutral	Asymmetry	-4.03e-04	0.998	Spearman	1
neutral_min	Aggregation	-	-	Spearman	-

Supplementary Table 7. Feature-level correlations between facial blendshape features and the MDS-UPDRS IV sum score in people with Parkinson's disease.

Supplementary Table 8| Clinical correlations with Hoehn and Yahr stage

Feature	Feature group	r	p value	Method	p_adj
browdownright	Activated	-0.55	8.07e-04	Pearson	0.031
mouthsmileleft	Activated	-0.49	0.00368	Pearson	0.143
browdownright_delta	Delta	-0.48	0.0052	Pearson	0.195
mouthsmileright	Activated	-0.47	0.00545	Pearson	0.212
mouthsmileright_delta	Delta	-0.47	0.00585	Pearson	0.219
mouthsmileleft_delta	Delta	-0.46	0.0073	Pearson	0.274
mouthlowerdownleft	Activated	-0.45	0.00902	Pearson	0.351
mouthleft	Activated	-0.45	0.00873	Spearman	0.339
eyewideleft	Activated	-0.44	0.014	Pearson	0.544
mouthleft_delta	Delta	-0.44	0.01	Spearman	0.386
mouthpucker × mouthleft	Interaction	-0.42	0.014	Spearman	0.33
mouthrolllower × mouthsmileleft	Interaction	-0.42	0.018	Pearson	0.422
eyewideright	Activated	-0.39	0.028	Pearson	1

mouthlowerdownleft_delta	Delta	-0.39	0.026	Pearson	0.988
eyewideright_delta	Delta	-0.39	0.029	Pearson	1
mouthleft × mouthsmileright	Interaction	-0.39	0.026	Pearson	0.634
eyeblinkleft_delta	Delta	0.37	0.042	Pearson	1
mouthpucker_neutral	Neutral	-0.36	0.05	Pearson	1
mouthshruglower × mouthsmileleft	Interaction	-0.35	0.053	Pearson	1
asym_eyelookupleft	Asymmetry	-0.35	0.062	Pearson	1
asym_eyelookupleft_delta	Asymmetry	-0.34	0.062	Pearson	1
mouthlowerdownright_delta	Delta	-0.34	0.079	Pearson	1
mouthshrugupper × mouthstretchright	Interaction	-0.34	0.055	Spearman	1
asym_mouthupperupleft_neutral	Asymmetry	-0.33	0.084	Pearson	1
eyeblinkright_delta	Delta	0.33	0.079	Pearson	1
mouthstretchright_delta	Delta	-0.32	0.071	Pearson	1
eyelookdownright	Activated	-0.32	0.072	Pearson	1
asym_eyesquintleft_delta	Asymmetry	-0.32	0.078	Pearson	1
asym_eyesquintleft	Asymmetry	-0.32	0.078	Pearson	1
asym_mouthlowerdownleft_neutral	Asymmetry	-0.32	0.083	Pearson	1
activated_mean	Aggregation	-0.31	0.08	Pearson	0.641
asym_nosesneerleft_neutral	Asymmetry	-0.31	0.083	Pearson	1
mouthfrownleft_delta	Delta	0.31	0.081	Pearson	1
browdownleft_delta	Delta	-0.3	0.087	Pearson	1
cheeksquintleft	Activated	-0.3	0.088	Pearson	1
mouthshrugupper × mouthsmileleft	Interaction	-0.3	0.09	Pearson	1
mouthfrownright_delta	Delta	0.3	0.091	Pearson	1
mouthlowerdownright_neutral	Neutral	-0.3	0.105	Pearson	1
asym_mouthleft_neutral	Asymmetry	-0.3	0.105	Pearson	1
asym_mouthlowerdownleft	Asymmetry	-0.3	0.101	Pearson	1
cheeksquintleft_delta	Delta	-0.29	0.097	Pearson	1

eyelookinright	Activated	-0.29	0.121	Pearson	1
mouthclose	Activated	0.29	0.103	Pearson	1
asym_eyesquintleft_neutral	Asymmetry	0.29	0.115	Spearman	1
mouthlowerdownleft_neutral	Neutral	-0.29	0.104	Pearson	1
mouthstretchright	Activated	-0.29	0.105	Pearson	1
cheeksquintright	Activated	-0.29	0.138	Pearson	1
asym_jawleft	Asymmetry	-0.29	0.111	Pearson	1
mouthleft × mouthrolllower	Interaction	-0.28	0.112	Pearson	1
eyeblinkleft	Activated	-0.28	0.144	Pearson	1
asym_mouthlowerdownleft_delta	Asymmetry	-0.28	0.124	Pearson	1
mouthleft × mouthshrugupper	Interaction	-0.28	0.121	Pearson	1
eyelookdownleft	Activated	-0.27	0.124	Pearson	1
asym_mouthfrownleft_neutral	Asymmetry	0.27	0.148	Spearman	1
asym_mouthleft	Asymmetry	-0.26	0.15	Pearson	1
mouthstretchleft_neutral	Neutral	-0.26	0.143	Pearson	1
mouthshruglower_delta	Delta	0.26	0.147	Pearson	1
browouterupleft	Activated	-0.25	0.164	Spearman	1
mouthleft × mouthrollupper	Interaction	-0.25	0.163	Spearman	1
asym_mouthstretchleft_neutral	Asymmetry	-0.25	0.18	Pearson	1
jawleft	Activated	-0.25	0.166	Pearson	1
mouthshrugupper × mouthsmileright	Interaction	-0.24	0.175	Pearson	1
eyelookinright_delta	Delta	-0.24	0.19	Pearson	1
eyelookoutright_neutral	Neutral	0.24	0.215	Pearson	1
mouthright_neutral	Neutral	0.24	0.191	Spearman	1
mouthfrownleft	Activated	0.24	0.177	Pearson	1
asym_mouthdimpleleft_delta	Asymmetry	0.24	0.188	Pearson	1
asym_eyelookoutleft_neutral	Asymmetry	-0.24	0.226	Pearson	1
asym_mouthleft_delta	Asymmetry	-0.24	0.201	Pearson	1

neutral_mean	Aggregation	-0.24	0.195	Pearson	1
mouthrollupper × mouthstretchright	Interaction	-0.23	0.205	Spearman	1
asym_mouthdimpleleft	Asymmetry	0.23	0.197	Pearson	1
jawleft_delta	Delta	-0.23	0.197	Pearson	1
mouthfrownright	Activated	0.23	0.202	Pearson	1
mouthrolllower × mouthsmileright	Interaction	-0.23	0.202	Pearson	1
eyeblinkright_neutral	Neutral	-0.23	0.209	Spearman	1
asym_jawleft_delta	Asymmetry	-0.23	0.226	Pearson	1
eyeblinkleft_neutral	Neutral	-0.23	0.211	Spearman	1
browdownleft	Activated	-0.23	0.207	Pearson	1
eyelookinleft_neutral	Neutral	0.22	0.216	Pearson	1
asym_eyeblinkleft_neutral	Asymmetry	-0.22	0.271	Pearson	1
delta_mean	Aggregation	-0.22	0.22	Pearson	1
asym_mouthpressleft_neutral	Asymmetry	0.22	0.222	Pearson	1
mouthstretchleft	Activated	-0.22	0.224	Pearson	1
mouthdimpleright	Activated	-0.22	0.234	Pearson	1
cheeksquintright_delta	Delta	-0.22	0.228	Pearson	1
asym_nosesneerleft	Asymmetry	-0.21	0.278	Pearson	1
browouterupleft_delta	Delta	-0.21	0.246	Pearson	1
eyelookdownleft_neutral	Neutral	-0.21	0.245	Pearson	1
eyelookdownright_neutral	Neutral	-0.21	0.246	Pearson	1
nosesneerleft_delta	Delta	-0.2	0.264	Spearman	1
tongueout	Activated	-0.2	0.304	Pearson	1
tongueout_delta	Delta	-0.2	0.306	Pearson	1
activated_range	Aggregation	-0.19	0.279	Pearson	1
mouthshruglower	Activated	0.19	0.28	Pearson	1
mouthrolllower × mouthshruglower	Interaction	0.19	0.282	Pearson	1
asym_browdownleft_delta	Asymmetry	0.19	0.286	Pearson	1

asym_browdownleft	Asymmetry	0.19	0.289	Pearson	1
eyelookoutleft_delta	Delta	-0.19	0.317	Pearson	1
eyelookoutleft	Activated	-0.19	0.317	Pearson	1
mouthsmileright_neutral	Neutral	0.18	0.342	Spearman	1
mouthfunnel_neutral	Neutral	-0.18	0.359	Pearson	1
mouthstretchright_neutral	Neutral	-0.18	0.325	Pearson	1
mouthleft_neutral	Neutral	-0.17	0.368	Spearman	1
mouthclose_delta	Delta	0.17	0.345	Pearson	1
asym_eyewideleft_neutral	Asymmetry	0.17	0.384	Pearson	1
mouthdimpleleft	Activated	-0.17	0.351	Pearson	1
mouthrolllower_neutral	Neutral	-0.17	0.359	Pearson	1
activated_min	Aggregation	0.16	0.361	Pearson	1
mouthshrugupper_neutral	Neutral	-0.16	0.375	Spearman	1
mouthlowerdownright	Activated	-0.16	0.405	Pearson	1
mouthdimpleleft_delta	Delta	-0.16	0.39	Pearson	1
mouthshruglower_neutral	Neutral	-0.16	0.39	Spearman	1
asym_nosesneerleft_delta	Asymmetry	-0.16	0.425	Pearson	1
asym_eyelookinleft	Asymmetry	0.16	0.389	Pearson	1
jawforward_delta	Delta	0.15	0.399	Pearson	1
delta_max	Aggregation	-0.15	0.408	Pearson	1
asym_eyelookinleft_neutral	Asymmetry	0.15	0.41	Pearson	1
mouthrollupper × mouthsmileleft	Interaction	-0.15	0.396	Pearson	1
mouthpressright_neutral	Neutral	-0.15	0.456	Pearson	1
jawright_neutral	Neutral	0.15	0.398	Spearman	1
mouthrollupper × mouthshrugupper	Interaction	-0.15	0.414	Spearman	1
mouthpucker × mouthshruglower	Interaction	0.15	0.41	Pearson	1
mouthshrugupper × mouthstretchleft	Interaction	-0.15	0.431	Spearman	1
mouthfrownright_neutral	Neutral	0.14	0.441	Pearson	1

asym_jawleft_neutral	Asymmetry	-0.14	0.435	Pearson	1
jawleft_neutral	Neutral	0.14	0.477	Spearman	1
mouthshruglower × mouthstretchright	Interaction	-0.14	0.437	Pearson	1
mouthrollupper × mouthshruglower	Interaction	0.14	0.447	Spearman	1
eyelookupright_delta	Delta	0.14	0.44	Pearson	1
mouthfrownleft_neutral	Neutral	0.14	0.467	Pearson	1
asym_browouterupleft_neutral	Asymmetry	-0.14	0.468	Pearson	1
mouthupperupleft	Activated	-0.13	0.47	Pearson	1
jawforward	Activated	0.13	0.475	Pearson	1
eyewideleft_neutral	Neutral	0.13	0.472	Spearman	1
eyewideright_neutral	Neutral	0.13	0.472	Spearman	1
browouterupright	Activated	-0.13	0.481	Pearson	1
mouthshruglower × mouthshrugupper	Interaction	0.13	0.479	Pearson	1
mouthfunnel_delta	Delta	0.13	0.494	Pearson	1
mouthpressleft	Activated	0.12	0.489	Pearson	1
mouthpressleft_delta	Delta	0.12	0.49	Pearson	1
browdownright_neutral	Neutral	0.12	0.502	Pearson	1
mouthrolllower_delta	Delta	0.12	0.497	Pearson	1
eyelookoutright_delta	Delta	-0.12	0.5	Pearson	1
browdownleft_neutral	Neutral	0.12	0.51	Pearson	1
asym_cheeksquintleft	Asymmetry	-0.12	0.519	Pearson	1
mouthleft × mouthshruglower	Interaction	-0.12	0.514	Spearman	1
asym_mouthstretchleft_delta	Asymmetry	0.12	0.523	Pearson	1
eyesquintright_delta	Delta	0.12	0.518	Pearson	1
mouthfunnel	Activated	0.11	0.553	Pearson	1
asym_eyeblinkleft_delta	Asymmetry	-0.11	0.572	Pearson	1
asym_eyeblinkleft	Asymmetry	-0.11	0.573	Pearson	1
cheeksquintleft_neutral	Neutral	-0.11	0.545	Pearson	1

mouthstretchleft_delta	Delta	-0.11	0.539	Pearson	1
mouthupperupleft_delta	Delta	-0.11	0.553	Pearson	1
mouthupperupleft_neutral	Neutral	-0.11	0.548	Pearson	1
mouthupperupright_neutral	Neutral	-0.11	0.543	Pearson	1
jawopen_neutral	Neutral	0.11	0.552	Pearson	1
asym_cheeksquintleft_delta	Asymmetry	-0.11	0.557	Pearson	1
eyelookupleft_neutral	Neutral	-0.11	0.601	Pearson	1
eyelookupright_neutral	Neutral	-0.11	0.602	Pearson	1
browouterupright_delta	Delta	-0.11	0.562	Pearson	1
asym_eyelookupleft_neutral	Asymmetry	-0.1	0.593	Pearson	1
asym_cheeksquintleft_neutral	Asymmetry	-0.1	0.588	Pearson	1
mouthshruglower × mouthstretchleft	Interaction	-0.1	0.587	Pearson	1
nosesneerright_neutral	Neutral	-0.1	0.614	Pearson	1
asym_eyewideleft	Asymmetry	0.09	0.649	Pearson	1
asym_mouthpressleft	Asymmetry	0.09	0.609	Pearson	1
browouterupleft_neutral	Neutral	0.09	0.634	Pearson	1
mouthrollupper × mouthsmileright	Interaction	-0.09	0.61	Pearson	1
browouterupright_neutral	Neutral	0.09	0.636	Pearson	1
asym_eyewideleft_delta	Asymmetry	0.09	0.657	Pearson	1
eyesquintright	Activated	0.09	0.614	Pearson	1
eyelookinright_neutral	Neutral	-0.09	0.631	Pearson	1
asym_mouthpressleft_delta	Asymmetry	0.09	0.626	Pearson	1
browinnerup_neutral	Neutral	-0.09	0.646	Pearson	1
mouthupperupright	Activated	-0.09	0.635	Pearson	1
asym_eyelookoutleft_delta	Asymmetry	0.09	0.633	Pearson	1
eyelookoutright	Activated	-0.09	0.636	Pearson	1
mouthshrugupper	Activated	-0.08	0.645	Pearson	1
jawopen_delta	Delta	-0.08	0.664	Pearson	1

eyelookoutleft_neutral	Neutral	0.08	0.656	Spearman	1
delta_min	Aggregation	0.08	0.658	Pearson	1
delta_range	Aggregation	-0.08	0.663	Pearson	1
eyelookinleft	Activated	0.08	0.665	Pearson	1
cheeksquintright_neutral	Neutral	-0.08	0.678	Pearson	1
activated_max	Aggregation	-0.07	0.702	Pearson	1
mouthpressright_delta	Delta	0.07	0.694	Pearson	1
jawopen	Activated	0.07	0.721	Spearman	1
neutral_range	Aggregation	0.07	0.699	Pearson	1
neutral_max	Aggregation	0.07	0.699	Pearson	1
mouthupperupright_delta	Delta	-0.07	0.704	Pearson	1
asym_mouthstretchleft	Asymmetry	0.07	0.712	Pearson	1
mouthpressright	Activated	0.07	0.716	Pearson	1
asym_mouthupperupleft_delta	Asymmetry	0.07	0.723	Pearson	1
eyelookdownleft_delta	Delta	-0.07	0.719	Pearson	1
mouthpucker × mouthshrugupper	Interaction	-0.07	0.719	Pearson	1
eyesquintleft_delta	Delta	-0.06	0.73	Pearson	1
browinnerup	Activated	-0.06	0.745	Pearson	1
activated_std	Aggregation	0.06	0.748	Pearson	1
asym_mouthupperupleft	Asymmetry	0.06	0.753	Pearson	1
cheekpuff_neutral	Neutral	0.06	0.754	Pearson	1
eyesquintright_neutral	Neutral	-0.06	0.757	Pearson	1
neutral_std	Aggregation	-0.06	0.759	Pearson	1
jawright_delta	Delta	-0.06	0.759	Pearson	1
eyesquintleft_neutral	Neutral	-0.06	0.759	Pearson	1
eyelookdownright_delta	Delta	0.06	0.761	Pearson	1
asym_mouthfrownleft_delta	Asymmetry	0.05	0.77	Pearson	1
asym_mouthsmileleft	Asymmetry	-0.05	0.777	Pearson	1
mouthrolllower × mouthrollupper	Interaction	0.05	0.788	Pearson	1

mouthpressleft_neutral	Neutral	-0.05	0.802	Pearson	1
eyelookupright	Activated	-0.05	0.802	Pearson	1
mouthright	Activated	0.04	0.807	Pearson	1
asym_eyelookdownleft_delta	Asymmetry	0.04	0.811	Pearson	1
asym_eyelookdownleft	Asymmetry	0.04	0.811	Pearson	1
tongueout_neutral	Neutral	-0.04	0.812	Pearson	1
cheekpuff	Activated	0.04	0.809	Spearman	1
asym_mouthfrownleft	Asymmetry	0.04	0.811	Pearson	1
nosesneerleft	Activated	-0.04	0.815	Pearson	1
mouthsmileleft_neutral	Neutral	0.04	0.823	Spearman	1
jawright	Activated	-0.04	0.818	Pearson	1
asym_eyelookinleft_delta	Asymmetry	0.04	0.818	Pearson	1
mouthpucker_delta	Delta	0.04	0.826	Pearson	1
asym_eyelookdownleft_neutral	Asymmetry	-0.04	0.827	Pearson	1
browinnerup_delta	Delta	0.04	0.827	Pearson	1
asym_eyelookoutleft	Asymmetry	0.04	0.839	Pearson	1
eyesquintleft	Activated	0.04	0.851	Pearson	1
mouthshruglower × mouthsmileright	Interaction	-0.03	0.854	Pearson	1
mouthrolllower × mouthshrugupper	Interaction	0.03	0.863	Pearson	1
eyeblinkright	Activated	0.03	0.877	Pearson	1
mouthright_delta	Delta	0.03	0.888	Pearson	1
mouthshrugupper × mouthupperupright	Interaction	-0.03	0.89	Pearson	1
mouthrollupper_delta	Delta	0.02	0.894	Pearson	1
jawforward_neutral	Neutral	-0.02	0.902	Spearman	1
mouthclose_neutral	Neutral	0.02	0.9	Pearson	1
nosesneerright	Activated	-0.02	0.902	Pearson	1
asym_mouthdimpleleft_neutral	Asymmetry	-0.02	0.91	Pearson	1
mouthshrugupper_delta	Delta	-0.02	0.91	Pearson	1

cheekpuff_delta	Delta	0.02	0.913	Spearman	1
mouthpucker	Activated	0.02	0.918	Pearson	1
eyewideleft_delta	Delta	-0.02	0.921	Pearson	1
eyelookupleft	Activated	-0.02	0.927	Pearson	1
nosesneerright_delta	Delta	0.02	0.93	Pearson	1
mouthpucker × mouthrollupper	Interaction	-0.02	0.932	Spearman	1
mouthpucker × mouthrolllower	Interaction	0.02	0.933	Pearson	1
asym_mouthsmileleft_neutral	Asymmetry	-0.01	0.94	Pearson	1
mouthdimpleright_neutral	Neutral	0.01	0.937	Pearson	1
asym_browdownleft_neutral	Asymmetry	-0.01	0.944	Pearson	1
asym_browouterupleft	Asymmetry	0.01	0.945	Pearson	1
asym_browouterupleft_delta	Asymmetry	0.01	0.946	Pearson	1
asym_mouthsmileleft_delta	Asymmetry	-0.01	0.946	Pearson	1
mouthrollupper	Activated	-0.01	0.947	Spearman	1
eyelookinleft_delta	Delta	0.01	0.972	Pearson	1
mouthdimpleleft_neutral	Neutral	-0.01	0.974	Pearson	1
mouthrollupper_neutral	Neutral	0.01	0.979	Pearson	1
mouthdimpleright_delta	Delta	0	0.979	Pearson	1
mouthrolllower	Activated	-0	0.983	Pearson	1
eyelookupleft_delta	Delta	-0	0.993	Pearson	1
delta_std	Aggregation	1.49e-04	0.999	Pearson	1
nosesneerleft_neutral	Neutral	-1.32e-04	0.999	Pearson	1
neutral_min	Aggregation	-	-	Pearson	-

Supplementary Table 8. Feature-level correlations between facial blendshape features and Hoehn and Yahr stage in people with Parkinson's disease.

Supplementary Table 9| Clinical correlations with levodopa equivalent dose (LED)

Feature	Feature group	r	p value	Method	p_adj
mouthfunnel	Activated	-0.56	9.99e-04	Spearman	0.039
mouthfunnel_delta	Delta	-0.49	0.00737	Pearson	0.276
asym_mouthupperupleft_neutral	Asymmetry	-0.42	0.019	Pearson	0.812
asym_eyewideleft_delta	Asymmetry	-0.42	0.04	Pearson	1
asym_eyewideleft	Asymmetry	-0.42	0.041	Pearson	1
asym_mouthlowerdownleft_neutral	Asymmetry	0.38	0.051	Pearson	1
mouthupperupleft_delta	Delta	-0.37	0.036	Pearson	1
asym_eyelookoutleft_delta	Asymmetry	-0.37	0.034	Pearson	1
mouthupperupleft	Activated	-0.37	0.037	Pearson	1
mouthupperupright	Activated	-0.37	0.035	Pearson	1
mouthupperupright_delta	Delta	-0.36	0.04	Pearson	1
mouthlowerdownright_neutral	Neutral	0.35	0.048	Spearman	1
asym_eyelookoutleft	Asymmetry	-0.34	0.052	Pearson	1
mouthdimpleright_neutral	Neutral	0.33	0.065	Pearson	1
mouthshruglower × mouthsmileleft	Interaction	-0.33	0.068	Pearson	1
mouthpucker × mouthshruglower	Interaction	-0.32	0.066	Pearson	1
mouthdimpleleft_neutral	Neutral	0.31	0.081	Pearson	1
mouthshruglower	Activated	-0.31	0.079	Pearson	1
jawopen_delta	Delta	-0.3	0.093	Pearson	1
mouthshruglower_delta	Delta	-0.3	0.091	Pearson	1
mouthlowerdownleft_neutral	Neutral	0.3	0.092	Spearman	1
mouthshrugupper × mouthupperupright	Interaction	-0.3	0.092	Pearson	1
eyelookoutleft	Activated	-0.29	0.098	Pearson	1
eyelookoutleft_delta	Delta	-0.29	0.098	Pearson	1
asym_mouthsmileleft	Asymmetry	-0.29	0.107	Pearson	1
eyesquintleft_delta	Delta	-0.29	0.112	Pearson	1

neutral_mean	Aggregation	0.29	0.107	Pearson	0.857
asym_mouthdimpleleft_neutral	Asymmetry	-0.28	0.121	Pearson	1
cheeksquintleft_neutral	Neutral	0.28	0.124	Pearson	1
eyesquintleft_neutral	Neutral	0.28	0.124	Pearson	1
eyesquintright_neutral	Neutral	0.28	0.124	Pearson	1
asym_mouthsmileleft_delta	Asymmetry	-0.27	0.128	Pearson	1
activated_mean	Aggregation	-0.27	0.131	Pearson	1
mouthshruglower × mouthshrugupper	Interaction	-0.27	0.126	Pearson	1
cheeksquintright_neutral	Neutral	0.26	0.154	Pearson	1
mouthleft × mouthrollupper	Interaction	0.26	0.148	Spearman	1
mouthleft × mouthshruglower	Interaction	-0.25	0.155	Pearson	1
mouthpucker_neutral	Neutral	0.25	0.177	Pearson	1
browouterupright_delta	Delta	-0.25	0.156	Spearman	1
mouthfrownright_neutral	Neutral	-0.25	0.186	Pearson	1
eyelookinright_delta	Delta	-0.25	0.175	Pearson	1
mouthstretchright	Activated	0.24	0.172	Pearson	1
asym_mouthsmileleft_neutral	Asymmetry	-0.24	0.2	Pearson	1
mouthrolllower × mouthsmileright	Interaction	0.24	0.195	Pearson	1
eyeblinkleft_delta	Delta	-0.23	0.222	Pearson	1
mouthrollupper × mouthsmileright	Interaction	0.23	0.191	Pearson	1
eyelookdownright_delta	Delta	-0.23	0.191	Pearson	1
eyelookinright	Activated	-0.23	0.207	Pearson	1
asym_eyelookoutleft_neutral	Asymmetry	0.23	0.236	Pearson	1
asym_browouterupleft_delta	Asymmetry	0.23	0.219	Pearson	1
asym_browouterupleft	Asymmetry	0.23	0.222	Pearson	1
jawright_neutral	Neutral	0.23	0.207	Spearman	1
mouthrollupper × mouthsmileleft	Interaction	0.23	0.207	Spearman	1
eyesquintleft	Activated	-0.22	0.22	Pearson	1

asym_eyelookupleft_delta	Asymmetry	0.22	0.221	Pearson	1
asym_eyelookupleft	Asymmetry	0.22	0.222	Pearson	1
browouterupright	Activated	-0.22	0.226	Spearman	1
mouthstretchright_delta	Delta	0.22	0.234	Pearson	1
mouthfrownleft_neutral	Neutral	-0.21	0.262	Pearson	1
mouthrollupper × mouthstretchright	Interaction	0.21	0.263	Spearman	1
asym_mouthlowerdownleft_delta	Asymmetry	0.2	0.265	Pearson	1
delta_mean	Aggregation	-0.2	0.273	Pearson	1
asym_mouthlowerdownleft	Asymmetry	0.19	0.288	Pearson	1
mouthleft	Activated	0.19	0.283	Spearman	1
mouthclose_delta	Delta	-0.19	0.291	Pearson	1
nosesneerleft_neutral	Neutral	0.19	0.309	Pearson	1
mouthlowerdownleft	Activated	0.19	0.295	Pearson	1
eyeblinkright_delta	Delta	-0.19	0.329	Pearson	1
asym_mouthpressleft_neutral	Asymmetry	-0.19	0.303	Pearson	1
mouthsmileright	Activated	0.18	0.304	Pearson	1
mouthleft_delta	Delta	0.18	0.323	Spearman	1
eyelookinleft	Activated	-0.18	0.323	Pearson	1
mouthfrownright	Activated	-0.18	0.327	Pearson	1
mouthright_neutral	Neutral	0.18	0.344	Spearman	1
eyelookdownright	Activated	-0.17	0.344	Pearson	1
mouthupperupleft_neutral	Neutral	0.17	0.348	Pearson	1
cheeksquintleft	Activated	0.16	0.364	Pearson	1
mouthrolllower × mouthshruglower	Interaction	-0.16	0.366	Pearson	1
asym_eyeblinkleft_neutral	Asymmetry	0.16	0.415	Pearson	1
asym_jawleft	Asymmetry	-0.16	0.417	Pearson	1
mouthleft × mouthsmileright	Interaction	0.16	0.378	Pearson	1
mouthleft_neutral	Neutral	-0.16	0.413	Pearson	1

mouthfrownleft_delta	Delta	0.16	0.414	Pearson	1
mouthrolllower_neutral	Neutral	0.16	0.39	Spearman	1
nosesneerright_neutral	Neutral	0.16	0.411	Pearson	1
mouthsmileright_delta	Delta	0.15	0.405	Pearson	1
asym_mouthfrownleft_neutral	Asymmetry	-0.15	0.427	Spearman	1
eyewideright_delta	Delta	0.15	0.429	Pearson	1
eyelookinleft_delta	Delta	-0.15	0.416	Pearson	1
eyesquintright_delta	Delta	-0.15	0.45	Pearson	1
mouthsmileright_neutral	Neutral	0.15	0.428	Spearman	1
mouthpucker × mouthshrugupper	Interaction	-0.14	0.421	Pearson	1
mouthlowerdownright	Activated	0.14	0.489	Pearson	1
browinnerup	Activated	-0.14	0.437	Spearman	1
mouthpressleft	Activated	0.14	0.444	Pearson	1
browdownleft	Activated	0.14	0.444	Pearson	1
asym_browdownleft_delta	Asymmetry	0.14	0.445	Pearson	1
asym_browdownleft	Asymmetry	0.14	0.445	Pearson	1
mouthfrownright_delta	Delta	-0.14	0.448	Pearson	1
mouthrolllower × mouthshrugupper	Interaction	-0.14	0.449	Pearson	1
browdownright_neutral	Neutral	0.14	0.45	Pearson	1
browdownleft_neutral	Neutral	0.14	0.45	Pearson	1
mouthshrugupper_delta	Delta	-0.13	0.457	Pearson	1
asym_mouthpressleft_delta	Asymmetry	0.13	0.471	Pearson	1
jawright	Activated	0.13	0.469	Pearson	1
asym_eyelookdownleft	Asymmetry	0.13	0.49	Spearman	1
asym_mouthupperupleft	Asymmetry	-0.13	0.484	Pearson	1
mouthshrugupper	Activated	-0.13	0.478	Pearson	1
asym_mouthpressleft	Asymmetry	0.13	0.487	Pearson	1
nosesneerleft_delta	Delta	0.13	0.484	Spearman	1
activated_min	Aggregation	-0.13	0.487	Pearson	1

mouthpressleft_delta	Delta	0.12	0.489	Pearson	1
mouthclose	Activated	-0.12	0.49	Pearson	1
asym_eyelookdownleft_delta	Asymmetry	0.12	0.507	Spearman	1
asym_mouthupperupleft_delta	Asymmetry	-0.12	0.502	Pearson	1
eyewideright	Activated	0.12	0.521	Pearson	1
neutral_std	Aggregation	0.12	0.512	Pearson	1
eyelookinright_neutral	Neutral	0.12	0.52	Spearman	1
activated_max	Aggregation	-0.12	0.529	Pearson	1
mouthshrugupper × mouthstretchright	Interaction	0.12	0.524	Spearman	1
mouthrollupper	Activated	0.12	0.52	Spearman	1
mouthdimpleright	Activated	0.12	0.543	Pearson	1
mouthstretchright_neutral	Neutral	0.12	0.53	Pearson	1
browinnerup_delta	Delta	-0.11	0.529	Pearson	1
eyewideright_neutral	Neutral	-0.11	0.536	Spearman	1
eyewideleft_neutral	Neutral	-0.11	0.536	Spearman	1
mouthdimpleright_delta	Delta	0.11	0.566	Pearson	1
jawright_delta	Delta	0.11	0.549	Pearson	1
cheeksquintright	Activated	0.11	0.55	Pearson	1
mouthsmileleft	Activated	-0.11	0.574	Pearson	1
mouthpressright_neutral	Neutral	0.11	0.583	Pearson	1
eyelookdownleft_neutral	Neutral	0.11	0.565	Pearson	1
eyelookdownright_neutral	Neutral	0.11	0.566	Pearson	1
nosesneerright_delta	Delta	0.1	0.564	Pearson	1
browinnerup_neutral	Neutral	0.1	0.59	Spearman	1
cheekpuff	Activated	-0.1	0.571	Spearman	1
asym_cheeksquintleft_delta	Asymmetry	0.1	0.572	Pearson	1
cheekpuff_delta	Delta	-0.1	0.575	Spearman	1
mouthstretchleft	Activated	0.1	0.579	Pearson	1

asym_mouthdimpleleft	Asymmetry	-0.1	0.59	Pearson	1
mouthshruglower × mouthstretchleft	Interaction	-0.1	0.585	Pearson	1
eyelookinleft_neutral	Neutral	-0.1	0.587	Pearson	1
mouthlowerdownleft_delta	Delta	0.1	0.593	Pearson	1
asym_mouthleft_delta	Asymmetry	0.1	0.607	Pearson	1
mouthrolllower_delta	Delta	-0.09	0.602	Pearson	1
asym_nosesneerleft_neutral	Asymmetry	0.09	0.612	Pearson	1
asym_mouthleft	Asymmetry	0.09	0.62	Pearson	1
tongueout_neutral	Neutral	0.09	0.615	Spearman	1
mouthpucker_delta	Delta	0.09	0.624	Pearson	1
eyelookoutright_delta	Delta	0.09	0.613	Pearson	1
asym_jawleft_neutral	Asymmetry	-0.09	0.618	Pearson	1
mouthpucker × mouthrollupper	Interaction	0.09	0.62	Spearman	1
asym_mouthdimpleleft_delta	Asymmetry	-0.09	0.628	Pearson	1
mouthshruglower × mouthsmileright	Interaction	-0.09	0.638	Pearson	1
asym_nosesneerleft	Asymmetry	0.09	0.657	Pearson	1
browdownright_delta	Delta	-0.09	0.641	Pearson	1
mouthshrugupper_neutral	Neutral	-0.09	0.644	Spearman	1
mouthrollupper_delta	Delta	0.08	0.646	Pearson	1
asym_eyelookdownleft_neutral	Asymmetry	0.08	0.646	Pearson	1
jawopen	Activated	-0.08	0.659	Pearson	1
cheeksquintleft_delta	Delta	0.08	0.654	Pearson	1
tongueout_delta	Delta	-0.08	0.661	Spearman	1
mouthupperupright_neutral	Neutral	0.08	0.659	Spearman	1
mouthsmileleft_delta	Delta	0.08	0.661	Pearson	1
tongueout	Activated	-0.08	0.668	Spearman	1
browouterupleft_neutral	Neutral	-0.08	0.689	Spearman	1
mouthshrugupper ×	Interaction	-0.08	0.673	Pearson	1

mouthsmileright					
mouthsmileleft_neutral	Neutral	0.08	0.684	Spearman	1
delta_max	Aggregation	-0.08	0.685	Pearson	1
asym_jawleft_delta	Asymmetry	-0.08	0.699	Pearson	1
asym_nosesneerleft_delta	Asymmetry	0.08	0.704	Pearson	1
mouthpucker	Activated	0.07	0.695	Pearson	1
mouthrollupper_neutral	Neutral	0.07	0.696	Pearson	1
nosesneerright	Activated	0.07	0.683	Pearson	1
mouthfrownleft	Activated	0.07	0.686	Pearson	1
asym_mouthstretchleft_delta	Asymmetry	0.07	0.708	Pearson	1
browouterupright_neutral	Neutral	-0.07	0.706	Spearman	1
eyelookoutleft_neutral	Neutral	-0.07	0.696	Spearman	1
asym_mouthstretchleft	Asymmetry	0.07	0.716	Pearson	1
mouthleft × mouthshrugupper	Interaction	-0.07	0.701	Pearson	1
activated_range	Aggregation	0.07	0.706	Pearson	1
mouthright	Activated	0.07	0.706	Pearson	1
eyeblinkleft	Activated	-0.07	0.722	Pearson	1
mouthright_delta	Delta	0.07	0.715	Pearson	1
mouthshrugupper × mouthstretchleft	Interaction	-0.07	0.717	Spearman	1
asym_eyelookinleft_neutral	Asymmetry	-0.07	0.719	Pearson	1
asym_eyelookinleft	Asymmetry	-0.06	0.743	Pearson	1
eyelookoutright	Activated	0.06	0.74	Pearson	1
asym_eyesquintleft_neutral	Asymmetry	-0.06	0.759	Spearman	1
delta_min	Aggregation	-0.06	0.746	Pearson	1
asym_eyesquintleft	Asymmetry	-0.06	0.764	Pearson	1
asym_eyesquintleft_delta	Asymmetry	-0.06	0.765	Pearson	1
mouthshruglower × mouthstretchright	Interaction	0.06	0.75	Pearson	1
mouthshruglower_neutral	Neutral	-0.06	0.752	Spearman	1

mouthpressleft_neutral	Neutral	-0.06	0.761	Spearman	1
activated_std	Aggregation	0.06	0.76	Pearson	1
cheekpuff_neutral	Neutral	-0.05	0.765	Pearson	1
asym_mouthleft_neutral	Asymmetry	-0.05	0.771	Pearson	1
eyesquintright	Activated	-0.05	0.778	Pearson	1
mouthrollupper × mouthshruglower	Interaction	-0.05	0.777	Spearman	1
asym_eyelookupleft_neutral	Asymmetry	-0.05	0.793	Pearson	1
asym_mouthstretchleft_neutral	Asymmetry	-0.05	0.787	Pearson	1
jawopen_neutral	Neutral	-0.05	0.797	Pearson	1
mouthpressright_delta	Delta	-0.05	0.79	Pearson	1
asym_cheeksquintleft	Asymmetry	0.05	0.793	Pearson	1
eyelookupleft	Activated	0.05	0.792	Pearson	1
browouterupleft	Activated	0.05	0.792	Spearman	1
mouthrolllower × mouthrollupper	Interaction	0.05	0.795	Pearson	1
neutral_range	Aggregation	-0.05	0.8	Pearson	1
neutral_max	Aggregation	-0.05	0.8	Pearson	1
eyelookupright_delta	Delta	-0.04	0.816	Pearson	1
eyelookdownleft_delta	Delta	-0.04	0.817	Pearson	1
mouthshrugupper × mouthsmileleft	Interaction	-0.04	0.828	Pearson	1
mouthpressright	Activated	-0.04	0.833	Pearson	1
mouthdimpleleft_delta	Delta	-0.04	0.837	Pearson	1
eyewideleft_delta	Delta	-0.04	0.839	Pearson	1
asym_mouthfrownleft_delta	Asymmetry	0.04	0.853	Pearson	1
jawforward_delta	Delta	0.03	0.85	Pearson	1
mouthclose_neutral	Neutral	-0.03	0.853	Spearman	1
mouthdimpleleft	Activated	0.03	0.851	Pearson	1
browdownleft_delta	Delta	0.03	0.86	Pearson	1
mouthpucker × mouthrolllower	Interaction	-0.03	0.864	Pearson	1
mouthstretchleft_delta	Delta	-0.03	0.866	Pearson	1

eyelookupleft_delta	Delta	0.03	0.867	Pearson	1
asym_eyewideleft_neutral	Asymmetry	-0.03	0.885	Spearman	1
mouthrolllower × mouthsmileleft	Interaction	-0.03	0.875	Pearson	1
eyelookupright	Activated	-0.03	0.88	Pearson	1
nosesneerleft	Activated	-0.03	0.884	Spearman	1
delta_std	Aggregation	0.03	0.885	Pearson	1
asym_eyeblinkleft_delta	Asymmetry	-0.03	0.901	Spearman	1
asym_eyeblinkleft	Asymmetry	-0.03	0.901	Spearman	1
browdownright	Activated	0.03	0.892	Pearson	1
mouthleft × mouthrolllower	Interaction	0.02	0.892	Pearson	1
jawleft_neutral	Neutral	-0.02	0.899	Spearman	1
eyewideleft	Activated	-0.02	0.902	Spearman	1
mouthrollupper × mouthshrugupper	Interaction	0.02	0.9	Spearman	1
jawleft	Activated	0.02	0.902	Pearson	1
jawforward_neutral	Neutral	-0.02	0.907	Spearman	1
eyeblinkright	Activated	-0.02	0.918	Pearson	1
eyelookupleft_neutral	Neutral	-0.02	0.924	Pearson	1
asym_mouthfrownleft	Asymmetry	0.02	0.923	Pearson	1
asym_browdownleft_neutral	Asymmetry	-0.02	0.923	Pearson	1
eyelookupright_neutral	Neutral	-0.02	0.932	Pearson	1
mouthstretchleft_neutral	Neutral	-0.01	0.939	Pearson	1
jawleft_delta	Delta	0.01	0.942	Pearson	1
mouthpucker × mouthleft	Interaction	0.01	0.943	Pearson	1
mouthlowerdownright_delta	Delta	-0.01	0.95	Pearson	1
mouthrolllower	Activated	0.01	0.946	Pearson	1
jawforward	Activated	-0.01	0.953	Pearson	1
eyeblinkright_neutral	Neutral	-0.01	0.962	Spearman	1
eyeblinkleft_neutral	Neutral	-0.01	0.962	Spearman	1

cheeksquintright_delta	Delta	0.01	0.964	Pearson	1
mouthfunnel_neutral	Neutral	-0.01	0.968	Pearson	1
browouterupleft_delta	Delta	-0.01	0.969	Pearson	1
asym_cheeksquintleft_neutral	Asymmetry	-0.01	0.972	Pearson	1
delta_range	Aggregation	-0	0.978	Pearson	1
asym_eyelookinleft_delta	Asymmetry	-0	0.98	Pearson	1
eyelookoutright_neutral	Neutral	0	0.983	Pearson	1
asym_browouterupleft_neutral	Asymmetry	-8.06e-04	0.997	Spearman	1
eyelookdownleft	Activated	-7.10e-04	0.997	Pearson	1
neutral_min	Aggregation	-	-	Pearson	-

Supplementary Table 9. Feature-level correlations between facial blendshape features and levodopa equivalent dose (LED) in people with Parkinson's disease. Correlations were residualised for age, sex, and Hoehn and Yahr stage. Correlation methods and *Meff*-based multiple-testing correction otherwise follow **Supplementary Table 5**.

References

1. Ekman P, Friesen WV. Facial Action Coding System: A Technique for the Measurement of Facial Movement. Palo Alto: Consulting Psychologists Press; 1978.
